# Mechanistic Studies on Regioselective Dephosphorylation of Phosphate Prodrugs during a Facile Synthesis of Antitumor Phosphorylated 2-Phenyl-6,7-methylenedioxy-1*H*-quinolin-4-one

**DOI:** 10.3390/molecules18078028

**Published:** 2013-07-08

**Authors:** Yung-Yi Cheng, Chin-Yu Liu, Li-Jiau Huang, Chi-Hung Huang, Kuo-Hsiung Lee, Cheng-Tung Lin, Sheng-Chu Kuo

**Affiliations:** 1 Graduate Institute of Pharmaceutical Chemistry, China Medical University, No.91 Hsueh-Shih Road, Taichung, 40402, Taiwan; 2 Graduate School of Biotechnology, Hung Kuang University, Taichung, No. 1018, Sec. 6, Taiwan Boulevard, Shalu District, Taichung, 43302, Taiwan; 3 Natural Products Research Laboratories, UNC Eshelman School of Pharmacy, University of North Carolina, Chapel Hill, NC 27599, USA; 4 Chinese Medicine Research and Development Center, China Medical University and Hospital, 2 Yuh-Der Road, Taichung, 40447, Taiwan; 5 Department of Chemistry, Science College, Tunghai University, No.1727, Sec.4, Taiwan Boulevard, Xitun District, Taichung, 40704, Taiwan

**Keywords:** MCF-7, prodrugs, dephosphorylation, antitumor activity

## Abstract

Phosphorylation of 2-(3-hydroxy-5-methoxyphenyl)-6,7-methylenedioxy-1*H*-quinolin-4-one (**1**) afforded diphosphate **2**. We found that, upon treatment with methanol under mild conditions, **2** can undergo facile and highly regioselective dephosphorylation to give the monophosphate **3**, with a phosphate group remaining on the phenyl ring. The details of the dephosphorylation process were postulated and then probed by LC-MS and HPLC analyses. Furthermore, as a preliminary study, the water soluble monophosphate prodrug **4** was tested for antitumor activity against a MCF-7 xenograft nude mice model.

## 1. Introduction

Numerous phosphoric esters have been developed as potential water-soluble prodrugs [1,2,3,4,5,6,7,8,9,10,11,12]. Many of them are marketed as injectable dosage forms, while only a few are used as oral dosage forms [11,12,13]. The phosphoric moiety is introduced into a parent drug molecule, which contains at least one hydroxyl functional group, in order to improve water solubility of the parent drug [9,10,11,12,13]. After administration, this phosphoric substituent will be cleaved enzymatically by endogenous phosphatases relatively easily [7,8,9,10,11,12,13,14]. In general, a parent drug incorporating a single phosphoric moiety has sufficient water solubility for use in an injectable dosage. Incorporation of more than one phosphoric moiety has no advantage, since the extra phosphoric moieties must also be removed by phosphatases. Furthermore, any parent drug still containing a residual phosphoric moiety will be highly polar and easily excreted prematurely through the GI or urinary system.

**Figure 1:**
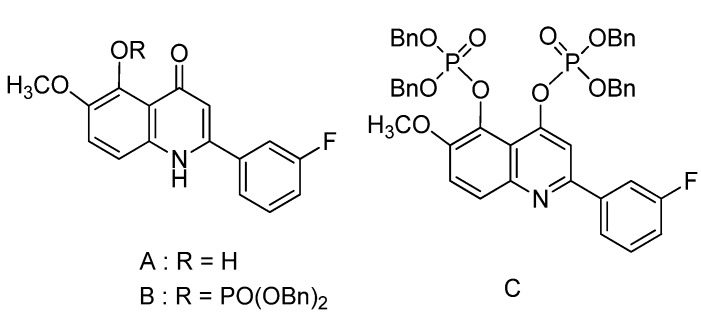
Structures of compounds **A**–**C**.

We recently reported a series of substituted 2-phenyl-1*H*-quinolin-4-ones and identified them as a new class of anticancer drugs [15,16,17,18,19]. In the course of synthesis, we found that phosphorylation of 2-(3-fluorophenyl)-1*H*-quinolin-4-one (**A**) with tetrabenzylpyrophosphate and NaH or K_2_CO_3_ in tetrahydrofuran solution afforded diphosphate **C**. The monophosphate **B** was obtained from the regioselective dephosphorylation of diphosphate **C** in methanol solution (Figure 1). Consequently, we were prompted to investigate the importance of methanol in the cleavage process and further evaluate the scope and mechanistic aspects of the reaction.

Previously, we reported that **1** acts as an antitumor agent by inducing both extrinsic and intrinsic apoptotic pathways via ROS-mediated activation of p38 MAPK signaling in HL-60 human leukemia cells *in vitro* [20]. In the present study, we selected the hydrophilic monophosphate of **1** as a target compound. This prodrug should be converted readily to the parent molecule in the bloodstream or GI tract by reaction with phosphatases. A similar strategy has successfully improved the clinical usage of etoposide, estramustine, and combretastin A-4 [21,22,23,24,25].

## 2. Results and Discussion

### 2.1. Chemistry

**Figure 2:**
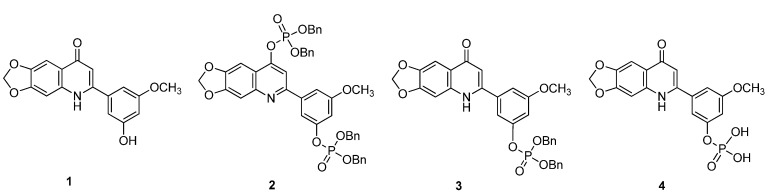
Structures of compounds **1**−**4**.

As part of our ongoing research on phosphate prodrugs, we wanted to prepare 3-(6,7-methylenedioxy-4-oxo-1,4-dihydroquinolin-2-yl)-5-methoxyphenyl dihydrogen phosphate (**4**), which contains a phosphate substituent on the 2-phenyl ring. We expected that this kind of analogue would have similar antitumor activity to related 2-phenyl-1*H*-quinolin-4-one anticancer drugs. In principle, phosphate **4** can be prepared easily from the appropriately substituted benzoic ester by a similar synthetic method as previously reported for 2-phenylquinolin-4-one derivatives [15,16,17,18,19]. Herein, we wish to detail our observations dealing with the dephosphorylation mechanism of diphosphate **2** in methanol solution, including the time period for the release of the phosphoric substituent from the dibenzyl 3-(4-((bis(benzoxy)phosphoryl)oxy)-6,7-methylenedioxyquinolin-4-yl)-5-methoxyphenyl phosphate (**2**) (Figure 2). 

**Scheme 1:**
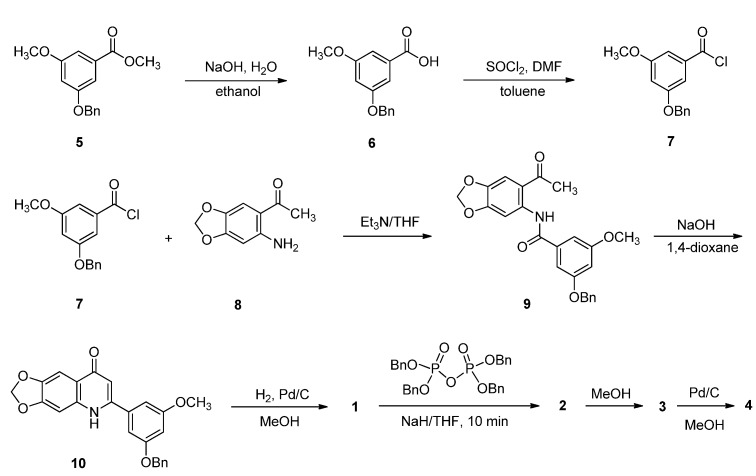
Synthesis of compounds **1**–**4**.

The syntheses of **1**–**4** are illustrated in Scheme 1. Initially, benzoic ester **5** was synthesized according to reported methods [26,27,28]. Saponification of **5** followed by acidification gave the corresponding benzoic acid **6**. Subsequent chlorination with thionyl chloride afforded 3-benzoxy-5-methoxybenzoyl chloride (**7**). Without purification, the resulting compound **7** was reacted with 2-amino-4,5-methlenedioxyacetophenone (**8**) to give the desired amide **9**, which was then cyclized in the presence of sodium hydroxide in refluxing dioxane solution to yield 2-(3-benzoxy-5-methoxyphenyl)-6,7-methylenedioxy-1*H*-quinolin-4-one (**10**). Catalytic hydrogenolysis of the resulting **10** with palladium on activated charcoal gave the key intermediate 2-(3-hydroxy-5-methoxyphenyl)-6,7-methylenedioxy-1*H*-quinolin-4-one (**1**). As expected, phosphorylation of **1** with two equivalents of tetrabenzyl pyrophosphate and sodium hydride in tetrahydrofuran solution provided the crystalline diphosphate **2** in 74% yield.

**Scheme 2:**
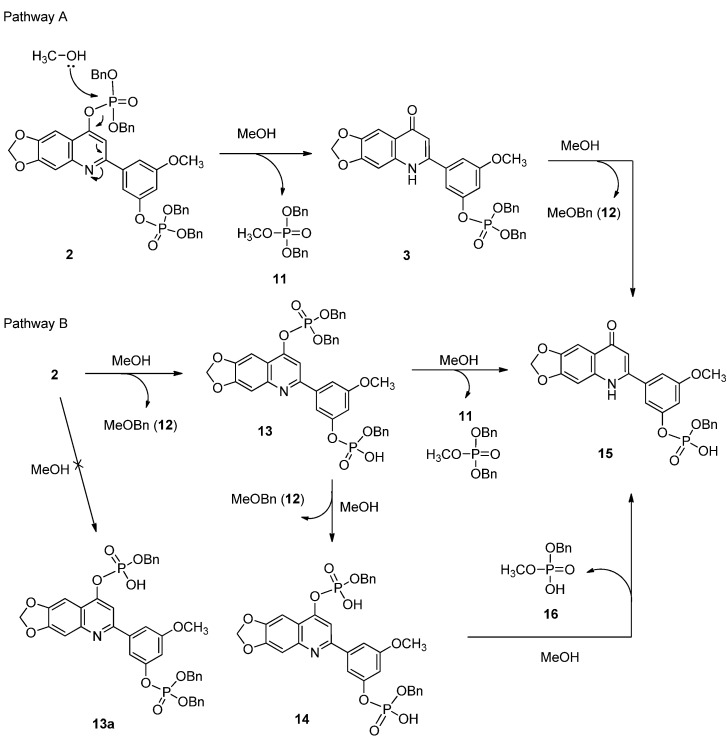
The pathways for dephosphorylation of diphosphate **2**.

Bis(dibenzyl phosphate) **2** was stable in acetonitrile/acetone solution at 25 ± 2 °C. However, it decomposed slowly in anhydrous methanol to form dibenzyl phosphate **3**, bis-phosphate derivatives **13** and **14**, and benzyl phosphate **15**, together with dibenzyl methyl phosphate (**11**) and benzyl methyl ether (**12**), as shown in Scheme 2. 

**Figure 3:**
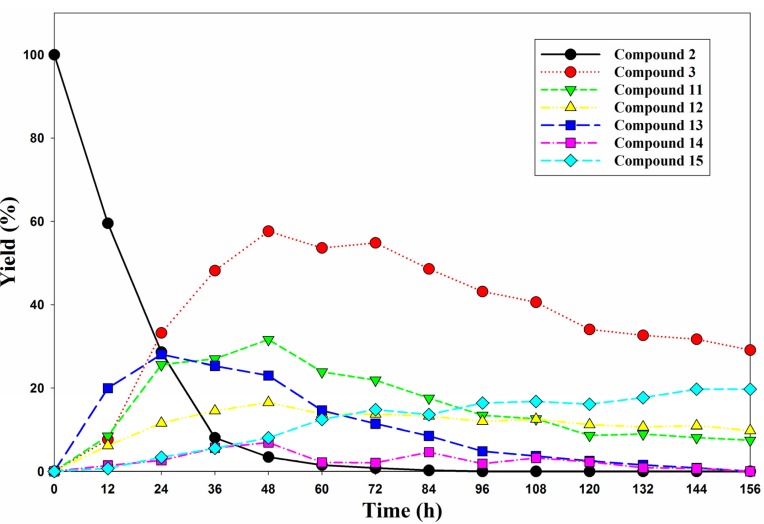
Distribution of products in the decomposition of **2** at various reaction times.

To investigate the reaction mechanism, all products were isolated in sufficient purity from the reaction mixture by using semi-preparative reversed phase column chromatography. LC-MS analyses of the reaction products are shown in Table 1. A close examination of molecular ion differences from the LC-ESIMS spectra over time revealed that benzyl groups are removed by methanol by the conversion of **3**→**15** in pathway A and of **2**→**13** plus **13**→**14** in pathway B. Additionally, a dibenzylphosphoryl group is removed in the transformation of **2**→**3**. To further establish the dephosphorylation pathways of diphosphate **2**, the product distribution was then monitored at different time intervals using HPLC analysis. The results are shown in Figure 3. The methanolysis of **2** was complete after 96 h and gave dibenzyl phosphate **3** and benzyl hydrogen phosphate **15** as the major detectable products. Dibenzyl phosphate **3** reached its highest detected distribution of 60% after 48 h. However, the isolated yield of **3** using flash column chromatography was 49% after 48 h. The formation of intermediate **13** increased at the beginning of the reaction and then decreased after 24 h. Both **13** and **14** disappeared completely after 156 h. Compound **15** is not as soluble as **2** and **3** in methanol, and after a few days, **15** precipitates slowly and influences the yields detected by HPLC. Therefore, after the reaction has proceeded for 96 h, a reduction of **3** does not cause an increase of **15**. 

**Table 1:** LC-MS analyses of the reaction products.

Compounds	*t_R_* (min)	HRESIMS ^a^ (*m/z*)	ESIMS ^a^ (*m/z*)	Formula
**2**	63.4	832.1916	832	C_45_H_40_NO_11_P_2_
**3**	46.9	572.1449	572	C_31_H_27_NO_8_P
**11**	46.5	ND ^b^	293	C_15_H_17_O_4_P
**12**	49.8	ND ^b^	123	C_8_H_10_O
**13**	39.8	742.1568	742	C_38_H_34_NO_11_P_2_
**14**	36.3	652.1158	652	C_31_H_28_NO_11_P_2_
**15**	38.3	482.0988	482	C_24_H_21_NO_8_P

^a^ Electrospray ionization mass spectrometer (ESIMS) and micrOTOF (HRESIMS) were operated in the positive mode [M+H]^+^ with full scan. ^b^ Not detected.

When compound **3** was isolated and re-subjected to the same reaction conditions, the decomposition products **12** and **15** were detected after 3 days, confirming that **15** was sufficiently stable in methanol solution. In an independent experiment, decomposition of **13** with methanol gave dephosphorylated product **15** regioselectively and phosphate **11** in 85% yield, accompanied with a small amount of **14**. Therefore, the removal of benzyl methyl hydrogen phosphate **16** via the decomposition of **14** was not detected successfully.

The structures of the isolated products **3**, **13**, **14**, and **15**, which were derived from decomposition of **2**, were established by mass and NMR spectroscopy analyses. Since the chemical shifts of the benzylic hydrogens in structure **2** are very close (absorptions at δ 5.28 and 5.22), we are unable to distinguish the dibenzylphosphoryl group attached on the 1*H*-quinolin-4-one skeleton from that on the phenyl ring by proton NMR spectroscopy. This same phenomenon happens in the carbon NMR absorptions; the benzylic carbons appear at δ 70.1 and 70.6. These results limited our ability to determine the position of the dibenzylphosphoryl groups in structure **13**. Cleavage of a benzyl group from either one of the dibenzylphosphoryl groups of **2** to form a benzyl hydrogen phosphoryl group will shift the benzylic hydrogens’ absorption from δ 5.28 or δ 5.22 downfield to δ 5.07. Similarly, the benzylic carbon will shift from δ 70.1 or δ 70.6 downfield to δ 67.6. With benzylic absorptions at δ 67.6 and 67.7, the structure of **14** is confirmed to have two benzyl hydrogen phosphoryl groups attached on the 2-phenylquinolin-4-one skeleton.

The uncertainty of determining whether a dibenzylphosphoryl group is attached on the quinoline moiety, as in **13**, or on the phenyl ring, as in **13a**, prompted us to use ^31^P-NMR spectroscopy. Comparison of the ^31^P-NMR proton decoupled spectra of **3** and **15** showed that, if a benzyl group is cleaved from the dibenzylphosphoryl group attached on the 2-phenyl ring to leave a benzyl hydrogen phosphoryl group, the phosphorus-31 signal will shift from δ −6.61 to δ −4.73. The ^31^P-NMR spectrum obtained for **14** exhibited two phosphorus signals at δ −4.82 and δ −5.44, suggesting that the phosphorus atom of the benzyl hydrogen phosphoryl group attached on the quinolin-4-one ring absorbs at δ −5.44. On the basis of these data, we were able to confirm that a signal at δ −5.14 in the ^31^P-NMR spectrum of **13** belongs to the phosphorus atom in the benzyl hydrogen phosphoryl group attached on 2-phenyl ring, while a second absorption at δ −6.87 must be due to the dibenzylphosphoryl group attached on the quinolin-4-one ring. Therefore, the structure of **13** was identified as benzyl 3-(4-((bis(benzyloxy)phosphoryl)oxy)-6,7-methylenedioxyquinolin-2-yl)-5-methoxyphenyl) hydrogen phosphate.

With this information in hand, a possible mechanism is shown in Scheme 2. Two pathways leading to the formation of **15** are suggested. Due to the electronic and resonance effects, a dibenzylphosphoryl group attached on the quinolin-4-one ring has been reported to undergo decomposition much faster than one on the phenyl ring in methanol solution [6]. Thus, the decomposition of diphosphate **2** in methanol solution is expected to proceed by an addition-elimination reaction on the dibenzylphosphoryl group of the quinolin-4-one skeleton and give monophosphate **3** as the only product. One of the benzyl groups in monophosphate **3** is then slowly cleaved by methanol to give **15**, which is highly stable in solution at ambient temperature. The attack of methanol on the benzylic carbon atom of a phosphate ester is unusual and has never been reported to our knowledge. The proposed mechanism of nucleophilic substitution was supported by the detection of benzyl methyl ether in the reaction mixture.

In the second pathway leading to **15**, one of the two benzyl groups in the dibenzylphosphoryl group attached on the phenyl ring of diphosphate **2** undergoes nucleophilic substitution by methanol to form compound **13**. Because benzyl methyl hydrogen phosphate **16** was not detected in the HPLC data and the yield of **14** was low (Figure 3), compound **15** should be derived predominately from **13** by dephosphorylation. Finally, hydrogenolysis of **3** or **15**, as well as diphosphate **2**, in the presence of palladium on charcoal produces 3-(6,7-methylenedioxy-4-oxo-1,4-dihydroquinolin-2-yl)-5-methoxyphenyl dihydrogen phosphate (**4**) in high yield as a stable and water soluble prodrug of the antitumor agent **1**.

### 2.2. *In Vitro* Biological Evaluation

**Figure 4:**
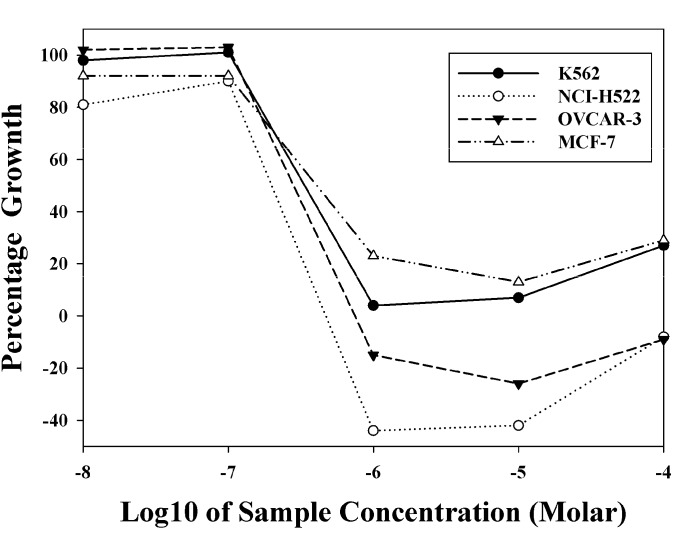
Dose-response curves of compound **1** against the sensitive cell lines.

The synthesized 2-(3-hydroxy-5-methoxyphenyl)-6,7-methylenedioxy-1*H*-quinolin-4-one (**1**) was evaluated for cell antiproliferative activity against human Hep3B hepatoma, Colo205 colon carcinoma, A498 renal carcinoma, NCI-H460 lung cancer, and Detroit 551 embryonic fibroblast cell lines. Compound **1** exhibited no significant cytotoxic activity against these five cell lines (IC_50_ > 50 μM). Compound **1** was submitted to US-NCI for evaluation of growth inhibitory activity against the NCI human cancer cell line panel. The results are shown in Table 2. The mean of logGI_50_ value of compound **1** was −4.73, indicating weak inhibitory activity against most cancer cell lines. In preliminary screening against 60 human cell lines, **1** demonstrated high selective inhibitory activity against NCI-H522 (non-small cell lung cancer), OVCAR-3 (ovarian cancer), K562 (leukemia) and MCF-7 (breast cancer) cell lines, showing logGI_50_ values of −6.70, −6.55, −6.47, and −6.39 (Figure 4, Supplementary Data).

**Table 2:** Inhibition of *in vitro* tumor cell growth by compound **1**.

Cell lines	logGI_50_	logTGI	logLC_50_
K562	−6.47	>−4.00	>−4.00
NCI-H522	−6.70	−6.33	>−4.00
OVCAR-3	−6.55	−6.13	>−4.00
MCF-7	−6.39	>−4.00	>−4.00

K562, leukemia cell line; NCI-H522, non-small cell lung cancer cell line; OVCAR-3, ovarian cancer cell line; MCF-7, breast cancer cell line. 2. GI_50_ = the concentration that causes 50% growth inhibition; TGI = the concentration that causes total growth inhibition; LC_50_ = the dosage of a given drug required to kill 50% of a test population.

### 2.3. *In Vivo* Antitumor Activity

**Figure 5:**
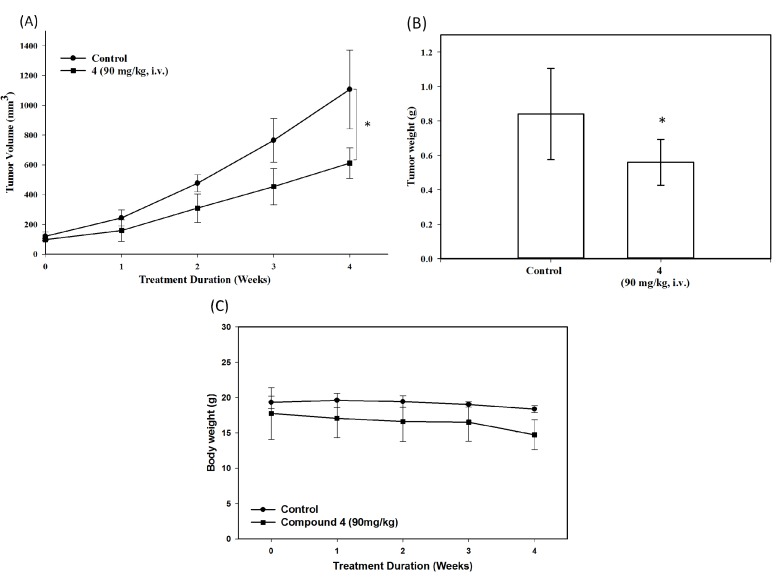
(**A**) Mean tumor volume-time profiles (**B**) Mean tumor weight-time profiles (**C**) Mean body weight-time profiles in MCF-7 xenograft nude mice (*n* = 4) following i.v. dosing of **4** at 90 mg/kg 5 days per week for 4 consecutive weeks. * *p* < 0.05 *vs.* control.

Based on the NCI-60 cell line screening results of **1**, we selected the MCF-7 xenograft model using dosing at 90 mg/kg (i.v.) to evaluate the *in vivo* antitumor activity of monophosphate prodrug **4** (Figure 5A−C). According to the results shown in Figure 5A, prodrug **4** induced time-dependent inhibition of MCF-7 tumor growth. During the course of antitumor evaluation, no significant body weight changes were detected in either the tested or the control mice (Figure 5C).

## 3. Experimental

### 3.1. Synthesis

#### 3.1.1. General

Commercial reagents were used without purification. TLC was performed on precoated silica gel 60 F_254_ (Merck) and spots were visualized by UV light at 254 nm. Silica gel 60 (Merck 70–230 mesh) was used for flash column chromatography. Melting points were determined on a Yanaco MP-500D apparatus and uncorrected. IR spectra were recorded on a Shimadzu IR Prestige-21 FTIR-8400S spectrophotometer. The one- and two-dimensional NMR spectra were recorded on either Bruker 500 AV II or Avance DPX-200 FT-NMR spectrometers at room temperature on solutions in CDCl_3_, D_2_O, CD_3_OD, or DMSO-*d_6_*. TMS was used as the internal standard for the ^1^H and ^13^C-NMR, and phosphoric acid was used as the external standard for ^31^P-NMR. HPLC was performed on a Shimadzu LC-10AT apparatus equipped with Shimadzu SPD-M10AVP diode-array detector and Shimadzu and Shimadzu SIL-10A auto-injector. Phenomenex Prodigy ODS3 100A, (5 μm, 250 × 4.6 mm i.d), Nucleodur^®^ C18 HTec (5 µm, 250 × 4.6 mm i.d), semi-preparative Nucleodur^®^ C18 HTec (5 µm, 250 × 10 mm i.d) and Thermo Hypersil ODS (5 µm,150 × 4.6 mm i.d) columns were used for analytical and preparative purposes, respectively. EIMS spectra were measured with HP 5995 GC–MS. The ESIMS spectra were obtained using the LC-ESIMS was performed on Agilent 1100 apparatus equipped with ESI mass spectrometer and with ultraviolet detection. The LC-ESI-HRMS spectra were obtained using LC-ESI-HRMS performed on an Agilent 1100 apparatus equipped with a Bruker micrOTOF orthogonal ESI-TOF mass spectrometer. 

#### 3.1.2. Synthesis of Compounds **5**−**7** and **9**

Methyl 3-(benzoxy)-5-methoxybenzoate (**5**, 4.45 g, 16.34 mmol) was dissolved in a solution of 95% ethanol (120 mL) and water (5 mL). Sodium hydroxide (2.00 g, 50.00 mmol) was added, and the reaction mixture was heated under reflux for one hour. After the reaction mixture was evaporated under vacuum, the residue was quenched with water (150 mL). The solution was neutralized with dilute HCl, and the precipitate was collected and washed with water and acetone to give **6** (3.79 g, 90%). Compound **6** (2.57 g, 9.95 mmol), thionyl chloride (4.80 g, 28.07 mmol) and *N*,*N*-dimethyl formamide (3 drops) were added in dry toluene (200 mL) and stirred at room temperature. The reaction mixture was stirred for 24 h and then evaporated to dryness. The crude product was used directly in the next step without further purification. Compounds **7** (2.77 g, 10.01 mmol) and **8** (1.79 g, 9.96 mmol) were suspended in dry THF (200 mL) and triethylamine (10 mL). The mixture was stirred at room temperature for 24 h and then evaporated. The residue was purified by silica gel column chromatography with CH_2_Cl_2_/EtOAc = 3:1 to obtain **9**.

*N-(6-Acetylbenzo[d][1,3]dioxol-5-yl)-3'-(benzoxy)-5'-methoxy-benzamide* (**9**)*.* Light yellow solid (3.15g, 75%). Mp: 151–152 °C. ^1^H-NMR (500 MHz, DMSO-*d*_6_): δ 2.64 (s, –COC*H*_3_,3H), 3.84 (s, –OC*H*_3_, 3H), 5.20 (s, –OC*H*_2_Ph, 2H), 6.19 (s, –OC*H*_2_O–, 2H), 6.88 (s, H-4, 1H), 7.09 (s, H-6, 1H), 7.16 (s, H-2, 1H), 7.36 (d, *J* = 7.43 Hz, ArH, 1H), 7.43 (t, *J* = 7.43 Hz, ArH, 2H), 7.49 (d, *J* = 7.43 Hz, ArH, 2H), 7.68 (s, H-5′, 1H), 8.34 (s, H-2′, 1H), 12.85 (s, -N*H*, 1H); ^13^C-NMR (125 MHz, DMSO-*d*_6_): δ 29.3 (–CO*C*H_3_), 56.0 (–O*C*H_3_), 70.1 (–O*C*H_2_Ph), 100.8 (C-2′), 102.9 (–O*C*H_2_O–), 105.0 (C-4), 105.7 (C-6), 106.5 (C-2), 111.3 (C-5′), 116.5 (C-6′), 128.5 (–OCH_2_*Ph*), 128.6 (–OCH_2_*Ph*), 129.0 (–OCH_2_*Ph*), 136.9 (C-1, and –OCH_2_*Ph*), 138.3 (C-1′), 143.1 (C-4′), 152.7 (C-3′), 160.3 (C-3), 161.2 (C-5), 165.0 (–NH*C*O), 201.8 (–*C*OCH_3_). IR: ν 3471, 2939, 2883, 2640, 1683, 1593, 1456, 1431, 1350, 1301, 1269, 1203, 1159, 1060, 1033, 952, 846, 732 cm^−1^. EIMS (70eV) *m/z*: 419.1 [M]^+^; LC-ESI-HRMS (Positive mode) *m/z*: [M+H]^+^ calcd for C_24_H_22_NO_6_, 420.1442; found 420.1438. 

*Methyl* *3-(benzoxy)-5-methoxybenzoate* (**5**). Colorless oil. ^1^H-NMR (200 MHz, CDCl_3_): δ 3.79 (s, 3H), 3.88 (s, 3H), 5.05 (s, 2H), 6.70 (t, *J* = 2.5 Hz, 1H), 7.18 (dd, *J* = 2.5, 1.2 Hz, 1H), 7.26 (dd, *J* = 2.5, 1.2 Hz, 1H), 7.29-7.46 (m, 5H); ^13^C-NMR (50 MHz, CDCl_3_): δ 52.2, 55.5, 70.2, 106.5, 107.5, 108.0, 127.5, 128.1, 128.6, 133.4, 136.5, 159.6, 160.6, 166.8. EIMS (70eV) *m/z*: 272.1 [M]^+^. 

*3-(Benzyloxy)-5-methoxybenzoic acid* (**6**). Colorless oil. ^1^H-NMR (200 MHz, CDCl_3_): δ 3.81 (s, 3H), 5.07 (s, 2H), 6.76 (t, *J* = 2.5 Hz, 1H), 7.25 (dd, *J* = 2.5, 1.2 Hz, 1H), 7.30–7.50 (m, 5H); ^13^C-NMR (50 MHz, CDCl_3_): δ 55.9, 69.9, 106.2, 107.6, 108.2, 128.1, 128.3, 128.9, 133.3, 137.2, 159.9, 160.8, 167.3. EIMS (70eV) *m/z*: 258.1 [M]^+^.

#### 3.1.3. Synthesis of Compound **10**

A mixture of **9** (3.33 g, 7.95 mmol) and NaOH (2.50 g, 62.50 mmol) was suspended in 1,4-dioxane (200 mL). The reaction mixture was refluxed for 12 h. After cooling to room temperature, the mixture was evaporated and then the residue was added to 10% NH_4_Cl solution (100 mL). The precipitate was collected and washed with water and acetone. The residue was purified by silica gel column chromatography with EtOAc to obtain **10**.

*2-(3-Benzoxy-5-methoxyphenyl)-6,7-methylenedioxy-1H-quinolin-4-one* (**10**)*.* Gray-white solid (5.21 g, 75%). Mp: 238–239 °C.^1^H-NMR (500 MHz, DMSO-*d*_6_): δ 3.85 (s, –OC*H*_3_, 3H), 5.22 (s, –OC*H*_2_Ph, 2H), 6.16 (s, –OC*H*_2_O–, 2H), 6.31 (s, br, H-3, 1H), 6.79 (s, H-4', 1H), 6.95 (s, H-6', 1H), 7.04 (s, H-2', 1H), 7.21 (s, H-8, 1H), 7.36 (m, ArH, 1H), 7.40–7.44 (m, ArH and H-5, 2H), 7.49–7.50 (m, 2H, ArH), 11.50 (s, 1H, –N*H*); ^13^C-NMR (125 MHz, DMSO-*d*_6_,): δ 56.0 (–O*C*H_3_), 70.1 (–O*C*H_2_Ph), 97.7 (C-8), 101.8 (C-5), 102.4 (–O*C*H_2_O–), 103.2 (C-4'), 106.0 (C-6'), 106.6 (C-2'), 107.2 (C-3), 120.5 (C-4a), 128.3 (–OCH_2_*Ph*), 128.4 (–OCH_2_*Ph*), 129.0 (–OCH_2_*Ph*), 137.3 (C-8a, C-1', and -OCH_2_*Ph*), 140.7 (C-2), 145.7 (C-6), 151.6 (C-7), 160.3 (C-3'), 161.2 (C-5'), 176.6 (C-4). IR: ν 3242, 3132, 3057, 2966, 1604, 1589, 1521, 1471, 1321, 1265, 1195, 1163, 1045, 941, 855, 842 cm^−1^. EIMS (70 eV) *m/z*: 401.1 [M]^+^; LC-ESI-HRMS (Positive mode) *m/z*: [M+H]^+^ calcd for C_24_H_20_NO_5_, 402.1336; found 420.1329.

#### 3.1.4. Synthesis of Compound **1**

A suspension of 0.50 mg (1.25 mmol) of **10** and 0.25 mg of palladium (10 wt% on activated carbon) in methanol (60 mL) was stirred at room temperature under hydrogen gas atmosphere for 24 h. The precipitate was collected and dissolved in 10% NaOH solution and then filtered. The filtrate was acidified with dil HCl and the precipitate was then collected and washed with acetone and water to obtain **1**.

*2-(3-Hydroxy-5-methoxyphenyl)-6,7-methylenedioxyquinolin-1H-4-one* (**1**). White solid (0.30 mg, 77%). Mp: >300 °C. ^1^H-NMR (500 MHz, DMSO-*d*_6_): δ 3.81 (s, –OC*H*_3_, 3H), 6.16 (s, –OC*H*_2_O–, 2H), 6.24 (s, br, H-3, 1H), 6.52 (s, H-4′, 1H), 6.77 (s, H-6′, 1H), 6.78 (s, H-2′, 1H), 7.22 (s, H-8, 1H), 7.40 (s, H-5, 1H), 9.91 (s, –O*H*, 1H), 11.56 (s, br, –N*H*, 1H); ^13^C-NMR (125 MHz, DMSO-*d*_6_): δ 55.8 (–O*C*H_3_), 97.8 (C-8), 101.5 (C-5), 102.4 (–O*C*H_2_O–), 103.3 (C-4′), 104.3 (C-6′), 106.7 (C-2′), 107.2 (C-3), 120.8 (C-4a), 136.8 (C-1′), 137.8 (C-8a), 145.7 (C-6), 149.3 (C-2), 151.6 (C-7), 159.4 (C-3′), 161.2 (C-5′), 176.1 (C-4). IR: ν 3383, 3250, 3153, 3010, 2974, 2839, 2704, 1635, 1616, 1591, 1531, 1496, 1489, 1471, 1429, 1338, 1315, 1265, 1199, 1165, 1056, 1035, 931, 854 cm^−1^. LC-ESI-MS (Positive mode) *m/z*: 312 [M+H]^+^; LC-ESI-HRMS (Positive mode) *m/z*: [M+H]^+^ calcd for C_17_H_13_NO_5_, 311.0794; found, 311.0800. The purity analysis was detected by reversed-phase HPLC on Thermo Hypersil ODS column (150 × 4.6 mm i.d) using an acetonitrile/0.02 M NaHCO_3_ (70:30) mixture as eluent. Flow rate was 0.3 mL/min and UV detector was set at 254 nm. The retention time of **1** was 5.4 min. The purity of **1** was 97.0%.

#### 3.1.5. Synthesis of Compound **2**

To a stirred solution of **1** (0.41 g, 1.32 mmol) in dry THF (40 mL) was added NaH (60% in mineral oil, 0.36 g) at 0 °C. After the mixture was stirred for 5 min, tetrabenzyl pyrophosphate (1.44 g, 2.68 mmol) was added and stirring was continued for 10 min. The reaction mixture was filtered and washed with THF. The filtrate was concentrated under vacuum at a temperature below 30 °C. The residue was purified by silica gel column chromatography with an eluent of *n*-hexane-EtOAc = 1:1 to obtain **2**.

*Dibenzyl 3-(4-((bis(benzoxy)phosphoryl)oxy)-6,7-methylenedioxyquinolin-4-yl)-5-methoxyphenyl phosphate* (**2**). White solid (0.82 g, 74%). Mp: 80–81 °C. ^1^H-NMR (500 MHz, CD_3_OD): δ 3.84 (s, –OC*H*_3_, 3H), 5.21 (d, ^3^*J*_HP_ = 9.4 Hz, –OPO(OC*H*_2_Ph)_2_, 2H), 5.22 (d, ^3^*J*_HP_ = 9.0 Hz,–OPO(OC*H*_2_Ph)_2_, 2H), 5.27 (d, ^3^*J*_HP_ = 10.7 Hz, –OPO(OC*H*_2_Ph)_2_, 2H), 5.28 (d, ^3^*J*_HP_ = 9.9 Hz, –OPO(OC*H*_2_Ph)_2_, 2H), 6.20 (s, –OC*H*_2_O–, 2H), 6.81 (s, H-6, 1H), 7.07 (s, H-3′, 1H), 7.28–7.40 (m, ArH, H-8′, H-2 and H-4, 22H), 7.46 (s, H-5′, 1H). ^1^H-NMR (200 MHz, CDCl_3_): δ 3.77 (s, –OC*H*_3_, 3H), 5.13 (d, ^3^*J*_HP_ = 8.3 Hz, –OPO(OC*H*_2_Ph)_2_, 4H), 5.16 (d, ^3^*J*_HP_ = 9.5 Hz,–OPO(OC*H*_2_Ph)_2_, 4H), 6.09 (s, –OC*H*_2_O–, 2H), 6.78 (m, H-6, 1H), 7.10 (s, H-3′, 1H), 7.23–7.40 (m, ArH, H-8′, H-2, H-4, and H-5′, 22H); ^13^C-NMR (50 MHz, CDCl_3_): δ 55.6 (–O*C*H_3_), 70.1 (–OPO(O*C*H_2_Ph)_2_), 70.6 (d, ^2^*J*_C-P_ = 5.0 Hz, –OPO(O*C*H_2_Ph)_2_), 97.2 (C-5′), 101.9 (–O*C*H_2_O–), 106.1 (C-8′), 106.6 (C-3′), 106.7 (d, ^3^*J*_C-P_ = 5.0 Hz, C-6), 110.0 (C-4), 111.5 (d, ^3^*J*_C-P_ = 5.0 Hz, C-2), 117.45 (d, ^3^*J*_C-P_ = 5.0 Hz, C-4a′), 128.1 (–OCH_2_*Ph*), 128.1 (–OCH_2_*Ph*), 128.6 (–OCH_2_*Ph*), 128.7 (–OCH_2_*Ph*), 128.9 (–OCH_2_*Ph*), 135.0 (d, ^3^*J*_C-P_ = 5.0 Hz, –OCH_2_*Ph*), 135.5 (d, ^3^*J*_C-P_ = 5.0 Hz, –OCH_2_*Ph*), 141.5 (C-3), 148.2 (C-6′, and C-8a′), 151.5 (C-7′), 151.7 (d, ^3^*J*_C-P_ = 5.0 Hz, C-1), 153.8 (d, ^2^*J*_C-P_ = 5.0 Hz, C-4′), 154.9 (C-2′), 160.8 (C-5); ^31^P-NMR (202.4947 MHz, CD_3_OD): δ −6.61, −6.77. IR: ν 3089, 3064, 3032, 2953, 2927, 2891, 2360, 1614, 1581, 1490, 1456, 1352, 1292, 1265, 1199, 1020, 962, 896, 746, 696 cm^−1^. LC-ESI-MS (Positive mode) *m/z*: 832 [M+H]^+^; LC-ESI-HRMS (Positive mode) *m/z*: [M+H]^+^ calcd for C_45_H_40_NO_11_P_2_, 832.2071; found, 832.1916. The purity analysis was detected by reversed-phase HPLC on Nucleodur^®^ C18 HTec (5 µm, 250 × 4.6 mm i.d) using a MeOH/0.02 M NaHCO_3_ (93:7) mixture as eluent. The flow rate was 0.5 mL/min and UV detector was set at 254 nm. The retention time of **2** was 11.2 min; the purity of **2** was 99.6%.

#### 3.1.6. Synthesis of Compounds **3** and **11**

A suspension of **2** (0.92 g, 1.11 mmol) in 100 mL of methanol was stirred at 25 °C for 48 h. The precipitate was collected and purified by silica gel column chromatography (EtOAc) to give **2** (0.05 g, 5%), **3** (0.31 g, 49%) and dibenzyl methyl phosphate **11** (0.52 g, 40%). 

*Dibenzyl 3-(6,7-methylenedioxyquinolin-2-yl)-5-methoxyphenyl phosphate* (**3**). Compound **3** was obtained as a white solid. Mp: 108–109 °C. ^1^H-NMR (500 MHz, CDCl_3_): δ 3.66 (s, –OC*H*_3_, 3H), 5.10 (d, ^3^*J*_HP_ = 9.1 Hz, –OPO(OC*H*_2_Ph)_2_, 4H), 6.01 (s, –OC*H*_2_O–, 2H), 6.37 (s, H-3′, 1H), 6.62 (s, H-6, 1H), 6.98 (s, H-8′, 1H), 7.16 (s, H-2, 1H), 7.28–7.40 (m, ArH and H-4, 11H), 7.61 (s, H-5′, 1H); ^13^C-NMR (125 MHz, CDCl_3_): δ 55.6 (–O*C*H_3_), 70.4 (d, ^2^*J*_C–P_ = 6.0 Hz, –OPO(O*C*H_2_Ph)_2_), 97.3 (C-5′), 101.8 (–O*C*H_2_O–), 102.3 (C-8′), 107.6 (C-3′), 107.8 (d, ^3^*J*_C–P_ = 4.2 Hz, C-2), 109.9 (C-6), 110.9 (d, ^3^*J*_C–P_ = 4.2 Hz, C-4), 121.0 (C-4a′), 128.1 (–OCH_2_*Ph*), 128.6 (–OCH_2_*Ph*), 128.9 (–OCH_2_*Ph*), 135.0 (d, ^3^*J*_C–P_ = 6.0 Hz, –OCH_2_*Ph*), 136.8 (C-8a′), 145.9 (C-2′), 148.1 (C-6′), 151.4 (d, ^3^*J*_C–P_ = 6.7 Hz, C-1), 151.9 (C-7′), 160.9 (C-5), 177.4 (C-4′); ^31^P-NMR (202.4947 MHz, CD_3_OD): δ −6.61. IR: ν 3234, 3095, 3066, 3008, 2960, 2927, 2846, 1635, 1602, 1589, 1521, 1471, 1259, 1155, 1035, 1012, 966, 854, 734, 696 cm^−1^. LC-ESI-MS (Positive mode) *m/z*: 572 [M+H]^+^; LC-ESI-HRMS (Positive mode) *m/z*: [M+H]^+^ calcd for C_31_H_27_NO_8_P, 572.1469; found, 572.1449. The purity analysis was detected by reversed-phase HPLC on Thermo Hypersil ODS column (150 × 4.6 mm i.d.) using a MeOH/0.01 M NaHCO_3_ (95:5) mixture as eluent. The flow rate was 0.5 mL/min. UV detector was set at 254 nm. The retention time of **3** was 3.9 min; the purity of **3** was 93.1%. 

*Dibenzyl methyl phosphate* (**11**). Compound **11** was obtained as colorless oil.^1^H NMR (200 MHz, CDCl_3_): δ 3.67 (d,^3^*J*_HP_ = 11.2 Hz, –OC*H*_3_, 3H), 5.02 (d, ^3^*J*_HP_ = 8.1 Hz, –OC*H*_2_Ph, 4H), 7.33 (s, br, ArH, 10H). ^13^C-NMR (50 MHz, CDCl_3_,): δ54.3 (d, ^2^*J*_C–P_ = 5.0 Hz, –O*C*H_3_), 69.3 (d, ^2^*J*_C–P_ = 5.0 Hz, –O*C*H_2_Ph), 127.9 (–OCH_2_*Ph*), 128.6 (–OCH_2_*Ph*), 135.8 (d, ^3^*J*_C–P_ = 6.6 Hz, –OCH_2_*Ph*). ^31^P-NMR (202.4947MHz, CD_3_OD): δ −0.16. LC-ESI-MS *m/z*: 293.0 [M+H]^+^.

#### 3.1.7. Synthesis of Compound **15**

A suspension of **2** (0.20 g, 0.24 mmol) in methanol (100 mL) was stirred at 25°C for 96 h. The precipitate was collected to give benzyl hydrogen phosphate **15** (0.04 mg, 33%). 

*Benzyl 3-(6,7-methylenedioxyquinolin-2-yl)-5-methoxyphenylhydrogen phosphate* (**15**). Light yellow solid. Mp: 60–61 °C; ^1^H-NMR (500 MHz D_2_O + NaOH): δ 3.70 (s, –OC*H*_3_, 3H), 4.90 (d, ^3^*J*_HP_ = 7.9 Hz, -OPO(OC*H*_2_Ph)OH, 2H), 5.89 (s, –OC*H*_2_O–, 2H), 6.59 (s, H-3′, 1H), 6.64 (s, H-2, 1H), 6.98 (s, H-4, 1H), 7.00 (s, H-6, 1H), 7.05 (s, H-8′, 1H), 7.12 (d, *J* = 7.2 Hz, ArH, 1H), 7.16 (d, *J* = 7.2 Hz, ArH, 2H), 7.23 (d, *J* = 7.4 Hz, ArH, 2H), 7.34 (s, H-5′, 1H); ^13^C-NMR (125 MHz, D_2_O + NaOH): δ 55.7 (–O*C*H_3_), 68.4 (d, ^2^*J*_C-__P_ = 5.4 Hz, –OPO(O*C*H_2_Ph)OH), 99.3 (C-5′), 101.4 (–O*C*H_2_O–), 103.6 (C-8′), 105.4 (C-3′), 106.3 (d, ^2^*J*_C-__P_ = 3.6 Hz, C-2), 108.9 (C-6), 112.2 (d, ^3^*J*_C-__P_ = 4.5 Hz, C-4), 120.9 (C-4a′), 127.9 (–OCH_2_*Ph*), 128.3 (–OCH_2_*Ph*), 128.7 (–OCH_2_*Ph*), 136.7 (d, ^3^*J*_C-__P_ = 7.3 Hz, –OCH_2_*Ph*), 142.8 (C-3), 145.2 (C-6′), 147.0 (C-8a′), 150.1 (C-7′), 152.7 (d, ^2^*J*_C-__P_ = 7.3 Hz, C-5), 156.9 (C-2′), 159.7 (C-1), 172.4 (C-4′); ^31^P-NMR (202.4947 MHz, CD_3_OD): δ −4.73. IR: ν 3396, 3070, 2922, 2835, 1869, 1645, 1608, 1479, 1421, 1273, 1157, 1085, 1035, 1012, 871, 851 cm^−1^. LC-ESI-MS (Positive mode) *m/z*: 482 [M+H]^+^; LC-ESI-HRMS (Positive mode) *m/z*: [M+H]^+^ calcd for C_24_H_21_O_8_NP, 482.0999; found, 482.0988.

#### 3.1.8. Synthesis of Compound **4**

A suspension of dibenzyl phosphate **3** (0.04 g, 0.07 mmol) in anhydrous methanol (20 mL) was hydrogenated in the presence of 10% Pd/C (0.02 g) at 25 °C for 15 min. The precipitate was collected and dissolved in 10% NaHCO_3_ solution and then filtered. The filtrate was acidified with dilute HCl and the precipitate was then collected and washed with acetone to obtain **4**.

*3-(6,7-Methylenedioxy-4-oxo-1,4-dihydroquinolin-2-yl)-5-methoxyphenyl dihydrogen phosphate* (**4**). White solid (0.02 g, 90%). Mp: > 300 °C. ^1^H-NMR (500 MHz, D_2_O + NaOH): δ3.87 (s, –OC*H*_3_, 3H), 6.02 (s, –OC*H*_2_O–, 2H), 6.77 (s, H-3′, 1H), 6.91 (s, H-6, 1H), 7.12 (s, H-2, 1H), 7.16 (s, H-8′, 1H), 7.23 (s, H-4, 1H), 7.44 (s, H-5′, 1H); ^13^C-NMR (125 MHz, D_2_O + NaOH): δ 55.7 (–O*C*H_3_), 99.4 (C-5′), 101.5 (–O*C*H_2_O–), 103.6 (C-8′), 105.4 (C-3′), 106.7 (C-6), 107.1 (C-2), 112.4 (C-4), 120.9 (C-4a′), 142.3 (C-3), 145.4 (C-6′), 147.1 (C-8a′), 150.3 (C-7′), 155.3 (C-5), 157.8 (C-2′), 159.8 (C-1), 172.6 (C-4′); ^31^P-NMR (202.4947 MHz, CD_3_OD): δ −3.86. IR: ν 3365, 3107, 2918, 2411, 1845, 1645, 1606, 1577, 1498, 1475, 1421, 1390, 1317, 1271, 1197, 1163, 1091, 1058, 1035, 935, 867, 839, 802, 528 cm^−1^. LC-ESI-HRMS (Negative mode) * m/z*: [M−H]^−^ calcd for C_17_H_13_O_8_NP, 390.0373; found, 390.0389. The purity analysis was detected by reversed-phase HPLC on a Thermo Hypersil ODS column (5 µm, 150 × 4.6 mm i.d) using a MeOH/9% NaHCO_3_ (70:30) mixture as eluent. The flow rate was 0.5 mL/min and UV detector was set at 254 nm. The retention time of **4** was 2.71 min; the purity of **4** was 99.4%.

### 3.2. Isolation Reaction Products from Reaction Mixture

After stirring for 24 h, the starting material **2** was dissolved in MeOH. The reaction mixture was separated by semi-preparative HPLC on Rp-18 column (Nucleodur^®^, C18 HTec, 5 μm, 250 × 10 mm i.d.) using a MeOH/0.02 M NaHCO_3_ (90:10) mixture solution as the mobile phase with flow rate 0.5 mL/min to obtain compounds **2**, **3**, **13**, **14**, and **15**.

*Benzyl* *3-(4-((bis(benzoxy)phosphoryl)oxy)-6,7-methylenedioxyquinolin-2-yl)-5-methoxyphenyl* *hydrogen phosphate* (**13**). Colorless oil. ^1^H-NMR (500 MHz, CD_3_OD): δ 3.83 (s, –OC*H*_3_, 3H), 5.07 (d, ^3^*J*_HP_ = 8.0 Hz, –OPO(OC*H*_2_Ph)OH, 2H), 5.20 (d, ^3^*J*_HP_ = 8.8 Hz, –OPO(OC*H*_2_Ph)_2_, 4H), 6.16 (s, –OC*H*_2_O–, 2H), 6.80 (s, H-4′, 1H), 7.30–7.40 (m, ArH, 19H), 7.45 (s, H-5, 1H), 7.80 (s, H-3, 1H); ^13^C-NMR (125 MHz, CD_3_OD): δ 54.9, 67.6, 70.3, 97.5, 102.0, 104.1, 106.0, 106.3, 109.7, 111.4, 117.3, 127.2, 127.4, 127.9, 128.0, 128.3, 128.5, 134.7, 136.5, 141.3, 146.5, 147.0, 150.3, 150.8, 154.5, 155.8, 160.0; ^31^P-NMR (202.4947MHz, CD_3_OD): δ −5.14, −6.87. IR: ν 3441, 3034, 2955, 2895, 1962, 1892, 1815, 1750, 1636, 1603, 1468, 1456, 1269, 1250, 1150, 1092, 1038, 1015, 966, 864, 736, 696 cm^−1^. LC-ESI-MS (Positive mode) *m/z*: 742 [M+H]^+^; LC-ESI-HRMS (Positive mode) *m/z*: [M+H]^+^ calcd for C_38_H_34_NO_11_P_2_, 742.1602; found, 742.1568.

*Benzyl* *3-((4-((benzoxy)phosphoryl)oxy)-6,7-methylenedioxyquinolin-2-yl)-5-methoxyphenyl* *hydrogen phosphate* (**14**). Colorless oil.^1^H-NMR (500 MHz, CD_3_OD): δ 3.83 (s, 3H, –OC*H*_3_), 5.05 (d, ^3^*J*_HP_ = 8.8 Hz, –OPO(OC*H*_2_Ph)OH, 2H), 5.06 (d, ^3^*J*_HP_ = 7.4 Hz, –OPO(OC*H*_2_Ph)OH, 2H), 6.15 (s, –OC*H*_2_O–, 2H), 7.04 (s, H-4′, 1H), 7.16–7.23 (m, 3H), 7.26–7.37 (m, 4H), 7.37–7.43 (m, 2H), 7.45 (s, H-5, 1H), 7.78 (s, H-3, 1H); ^13^C-NMR (125 MHz, CD_3_OD): δ 54.5, 67.6, 67.8, 97.4, 101.8, 104.1, 106.3, 106.4, 107.9, 111.7, 117.7, 126.7, 127.1, 127.4, 127.8, 137.3, 137.5, 141.6, 147.4, 147.7, 151.3, 154.1, 156.2, 156.5, 160.7; ^31^P-NMR (202.4947 MHz, CD_3_OD): δ -4.82, -5.44. IR: ν 3441, 3065, 3034, 2955, 2895, 2852, 1603, 1558, 1522, 1470, 1456, 1250, 1150, 1092, 1036, 1022, 968, 864, 736, 698 cm^−1^. LC-ESI-MS (Positive mode) *m/z*: 652 [M+H]^+^; LC-ESI-HRMS (Positive mode): *m/z* [M+H]^+^ calcd for C_31_H_28_NO_11_P_2_, 652.1132; found, 652.1158.

### 3.3. Reaction Products Analysis by HPLC-ESI-MS

On-line coupling of HPLC with ESI mass spectrometry has been used to analyze reaction mechanisms [29,30]. After stirring starting material **2** (4 mg/mL) in MeOH for 72 h, the, separation was achieved on ODS analytical column (Phenomenex Prodigy ODS3 100A, 5 μm, 250 × 4.6 mm i.d) using a gradient elution of acetonitrile and 0.04 mM NH_4_OH/H_2_O (pH = 9) in the following ratios 95% of water at 0 min, 88% of water at 5 min, 80% of water at 25 min, 54% of water at 30 min, 34% of water at 35−50 min, 1% of water at 55−65 min, 95% of water at 66−76 min. The flow rate was 0.5 mL/min and the column temperature was maintained at 25–28 °C. The reaction was then monitored by LC-MS. An electrospray ionization (ESI) mass spectrometer and micrOTOF were operated in the positive mode with full scan.

### 3.4. Bioassay

#### 3.4.1. Cell Culture

Human renal carcinoma A498, colon cancer (Colo205) and non-small-cell-lung cancer (NCI-H460) cells were maintained in RPMI-1640 medium supplemented with 10% fetal bovine serum (GIBCO/BRL), penicillin (100 U/mL)/streptomycin (100 μg/mL)(GIBCO/BRL) and 1% L-glutamine (GIBCO/BRL) at 37 °C in a humidified atmosphere containing 5% CO_2_. Human hepatocellular carcinoma cell line Hep3B was obtained from America Type Culture Collection (Manassas, VA, USA). Hep3B cells were cultured in DMEM/F12 medium supplemented with 10% FBS, and penicillin (100 U/mL)/streptomycin (100 μg/mL) and maintained in a humidified incubator containing 5% CO_2_. Normal skin Detroit 551 cells were maintained in DMEM medium supplemented with 10% fetal bovine serum (GIBCO/BRL), penicillin (100 U/mL)/streptomycin (100 μg/mL) (GIBCO/BRL) and 1% L-glutamine (GIBCO/BRL) at 37 °C in a humidified atmosphere containing 5% CO_2_. Logarithmically growing cancer cells were used for all experiments.

#### 3.4.2. *In vitro* Cell Viability Assay

Cell viability was detected by 3(4,5-dimethythiazol-2-yl)-2,5-diphenyltetrazolium bromide (MTT) assay. Cells were cultured in 96-well plates at 37 °C and incubated with complete medium containing the vehicle (DMSO) or compounds for indicated times and concentrations. After treatment, cells were incubated with MTT solution (1 mg/mL in 1× PBS) at 37 °C for 2 h. The absorbance of the samples was read at wavelength of 570 nm and corrected for inference at 630 nm.

#### 3.4.3. *In Vivo* Antitumor Activity Assay

The animals used in this experiment had access to food and water *ad libitum*. Experimental procedures using animals were approved by the Institutional Animal Care and Use Committees of The National Health Research Institutes. Nude female BALB/c mice (18–20 g; 6–8 weeks of age) were purchased from The National Laboratory Animal Center, Taipei, Taiwan, and maintained in pressurized ventilated cages according to institutional regulations. Human breast cancer MCF-7 (ATCC HTB-22) cells were cultured in DMEM with 10% heat-inactivated FBS and incubated at 37 °C in a humidified atmosphere containing 5% CO_2_. Each nude mouse was subcutaneously inoculated with 3 × 10^6^ MCF-7 cells in 0.2 mL PBS via a 24-gauge needle. After the injection of tumor cells, the animals were injected with beta-estradiol every 2 days. After the appearance of a 100 mm^3^ tumor nodule, the tumor-bearing mice were randomly divided into 2 groups. Compound **4** was administered by i.v. injection at 90 mg/kg on 5 days every week for 4 consecutive weeks. The animals were weighed and the tumors were measured using calipers twice a week before, during, and after drug treatment. The tumor volume was calculated with the following formula: 1/2 (L×W^2^), where L is the length and W is the width of the tumor [31]. At the end of the experiments, the animals were euthanized with carbon dioxide followed by cervical dislocation. 

## 4. Conclusions

Our present studies have demonstrated that dephosphorylation of an *O*-dibenzylphosphate moiety on quinolin-4-one rings can be carried out in a methanol solution at ambient temperature resulting in the formation of various phenyl dihydrogen phosphate systems. The reaction mechanism for the decomposition of **2** is readily explained by a sequence of addition-elimination reactions. The results reported herein indicate that dephosphorylation of an *O*-dibenzylphosphate or an *O*-benzyl hydrogen phosphate moiety on the quinolin-4-one ring is much faster than that on the phenyl ring. Finally, hydrogenolysis of **3** produces monophosphate **4** in high yield as a stable and water soluble prodrug of the antitumor agent **1**. Based on the NCI-60 cell line screening data of **1**, we selected the MCF-7 xenograft model to demonstrate that the monophosphate prodrug **4** inhibited the growth of MCF-7 tumor by about 44%, at 90 mg/kg/day dosage, without causing significant toxicity. 

## Supplementary Materials

Supplementary materials can be accessed at: http://www.mdpi.com/1420-3049/18/7/8028/s1.
