# Supplementary material for: Mechanistic Studies on Regioselective Dephosphorylation of Phosphate Prodrugs during a Facile Synthesis of Antitumor Phosphorylated 2-Phenyl-6,7-methylenedioxy-1H-quinolin-4-one
Source: Molecules. 2013 Jul 8;18(7):8028–45. doi: 10.3390/molecules18078028 (PMC6270573; doi:10.3390/molecules18078028)

Supporting Information

Table of Contents

Experimental Section

1. LC-ESI-MS and LC-ESI-HRMS Data……………………………………………………….......... S2–3
2. Method of Monitoring the Change in Reaction Products by HPLC………………………………..S4
3. NMR Spectra of Compounds **1**–**4**, and **9**–**15**……………………………………………..…S5–43
4. The Purity of Compounds **1**–**4**…………………………………………………………...S44–45
5. Mean Graph Presentation of Differential Data for Compound **1**…………………..........................S46

Experimental Section

1. LC-ESI-MS data

The on-line coupling of HPLC with ESI mass spectrometry has been used to analyze reaction mechanism. After stirring for 72 h, the starting material **2** (4 mg/mL in MeOH) separation was achieved on ODS analytical column (Phenomenex Prodigy ODS3 100A, 5 µm, 250 × 4.6 mm i.d) with using a gradient elution of acetonitrile and 0.04 mM NH_4OH/H_2O (Ph = 9) in the ratio 95% of water at 0 min, 88% of water at 5 min, 80% of water at 25 min, 54% of water at 30 min, 34% of water at 35–50 min, 1% of water at 55–65 min, 95% of water at 66–76 min. The flow rate was 0.5 mL/min and the temperature of column was maintained at 25–28 °C. An Electrospray ionization (ESI) mass spectrometer and micrOTOF were operated in the positive mode with full scan.

Figure S1. UV chromatographs of the reaction mixture.

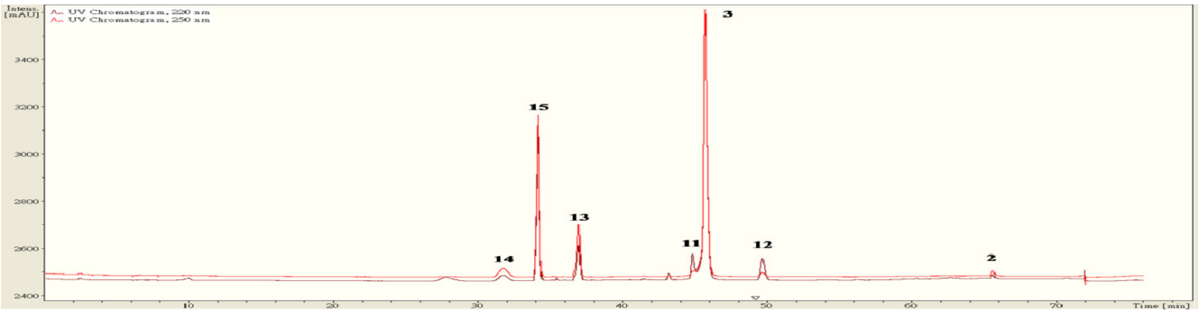

Figure S2. EIC chromatographs of compounds **2**, **3** and **11–15**.

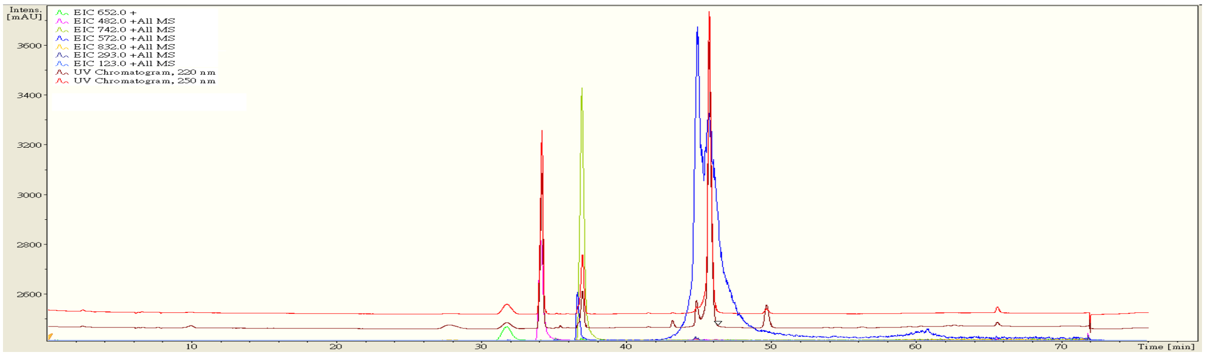

LC-ESI-HRMS data

Table S1. LC-ESI-HRMS Data of Compounds **2**, **3**, and **13–15**.

Rt. (min)	Compd	Meas. *m/z*	Formula	m/z	err [ppm]	Mean err [ppm]	rdb	N-Rule	e^- Conf	mSigma	Std I	Std Mean m/z	Std I VarNorm	Std m/z Diff	Std Comb Dev
36.3	**14**	652.1158	$C_{31}H_{28}NO_{11}P_2$	652.1132	-4.1	-3.4	19.5	ok	even	12.25	0.0197	0.0026	0.0091	0.0037	0.7925
38.3	**15**	482.0988	$C_{24}H_{21}NO_8P$	482.0999	2.4	2.8	15.5	ok	even	2.79	0.0044	0.0015	0.0018	0.0021	0.5626
39.8	**13**	742.1568	$C_{38}H_{34}NO_{11}P_2$	742.1602	4.5	5	23.5	ok	even	16.48	0.0278	0.0038	0.0115	0.0023	0.8223
46.9	**3**	572.1449	$C_{31}H_{27}NO_8P$	572.1469	3.5	3.8	19.5	ok	even	5.91	0.0096	0.0022	0.003	0.0011	0.8427
63.4	**2**	832.1916	$C_{45}H_{40}NO_{11}P_2$	832.2071	18.6	18.2	27.5	ok	even	95.81	0.1272	0.0152	0.0559	0.0055	0.8095

Figure S3. EIC chromatographs of the reaction mixture (Positive mode $[M+H]^+$).

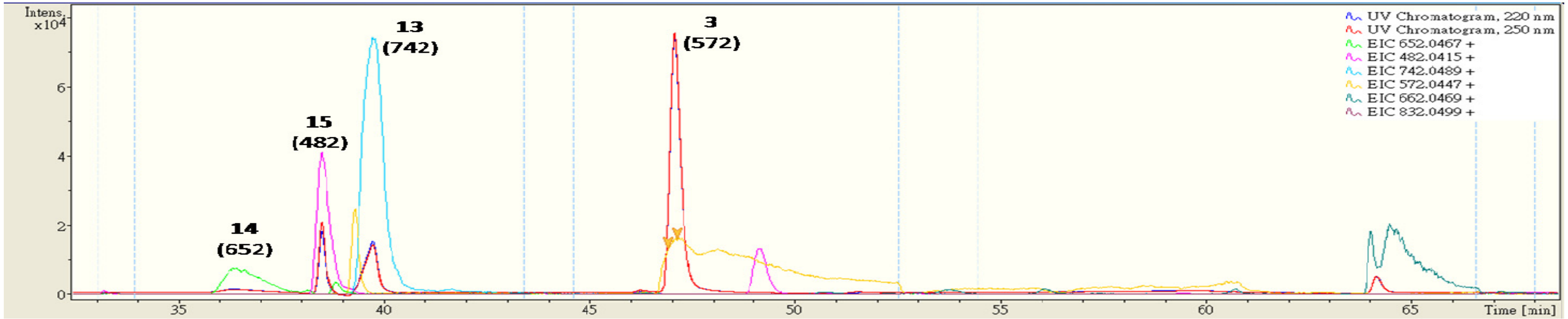

2. Using HPLC to Monitor the Changes in Reaction Products

The starting material **2** (4 mg) was dissolved in methanol (4 mL). Compound **2** was monitored every 12 h. The yields of reaction components were monitored by HPLC on C18 column (Nucleodur® C18 HTec, 5 µm, 250 × 4.6 mm i.d.) using a MeOH/0.02M $NaHCO_3$ (93/7 v/v) solvent mixture as eluent with UV monitoring by detection at 220 nm. The flow rate was 0.5 mL/min. All results were representative of 3 independent experiments.

	**Yield %**						
**Compounds**	**2**	**3**	**11**	**12**	**13**	**14**	**15**
0 h	100.0	0.0	0.0	0.0	0.0	0.0	0.0
12 h	59.5	7.6	8.5	6.2	19.9	1.4	0.7
24 h	28.6	33.2	25.6	11.5	28.0	2.6	3.4
36 h	8.1	48.1	26.9	14.5	25.3	5.6	5.5
48 h	3.4	57.6	31.6	16.5	23.0	6.9	8.0
60 h	1.5	53.6	23.9	13.7	14.6	2.1	12.4
72 h	0.8	54.9	21.9	13.7	11.4	2.0	14.8
84 h	0.2	48.6	17.5	13.2	8.5	4.6	13.6
96 h	0.0	43.1	13.4	12.0	4.8	1.8	16.4
108 h	0.0	40.6	12.6	12.5	3.6	3.2	16.7
120 h	0.0	34.0	8.6	11.2	2.5	2.3	16.1
132 h	0.0	32.6	9.0	10.6	1.5	0.8	17.7
144 h	0.0	31.7	8.1	10.9	0.8	0.7	19.7
156 h	0.0	29.1	7.5	9.8	0.0	0.0	19.7

3. NMR Spectra of Compounds 1–4, and 9–15

➢ *NMR Spectra of compound **1***

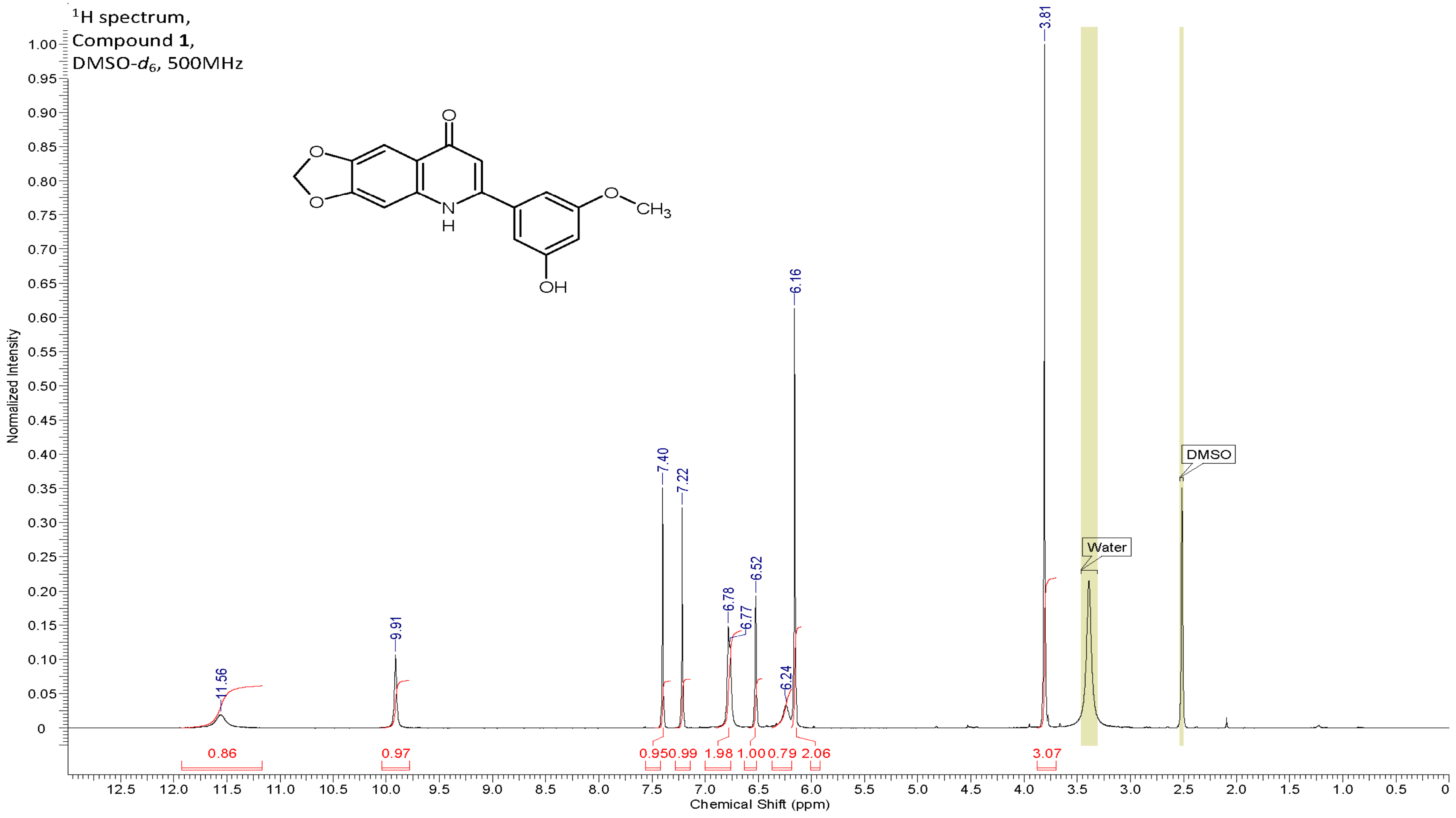

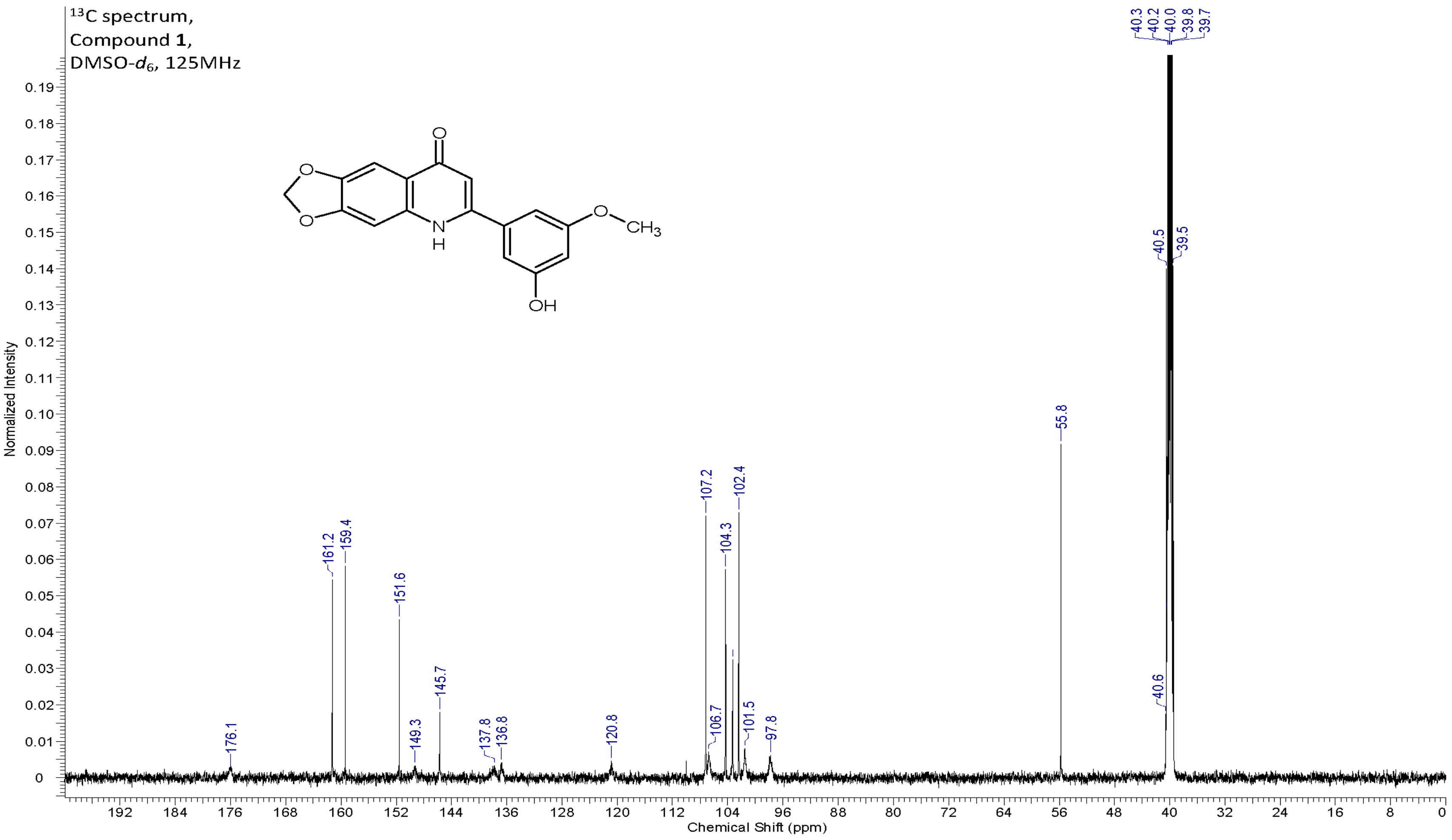

13C spectrum,
Compound 1,
DMSO-d6, 125MHz
Normalized Intensity
Chemical Shift (ppm)
O
O
O
N
H
O
CH3
OH
176.1
161.2
159.4
151.6
149.3
145.7
137.8
136.8
120.8
107.2
106.7
104.3
102.4
101.5
97.8
55.8
40.6
40.5
40.3
40.2
40.0
39.8
39.7
39.5

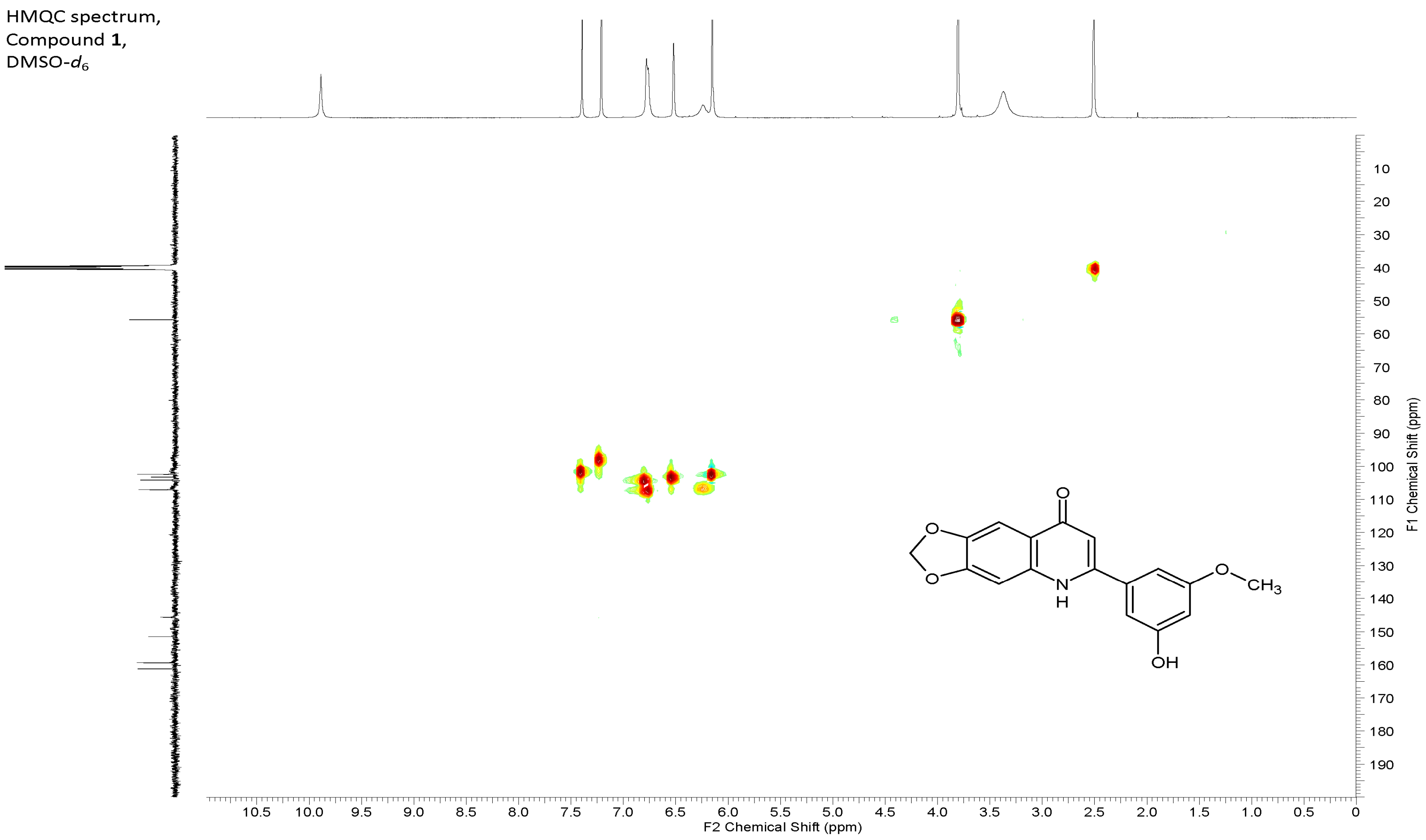

HMQC spectrum,
Compound **1**,
DMSO-d_6
10.5 10.0 9.5 9.0 8.5 8.0 7.5 7.0 6.5 6.0 5.5 5.0 4.5 4.0 3.5 3.0 2.5 2.0 1.5 1.0 0.5 0
F2 Chemical Shift (ppm)
10 20 30 40 50 60 70 80 90 100 110 120 130 140 150 160 170 180 190
F1 Chemical Shift (ppm)
O
N
H
O
O
O
CH3
OH

HMBC spectrum, Compound **1**, DMSO-d_6

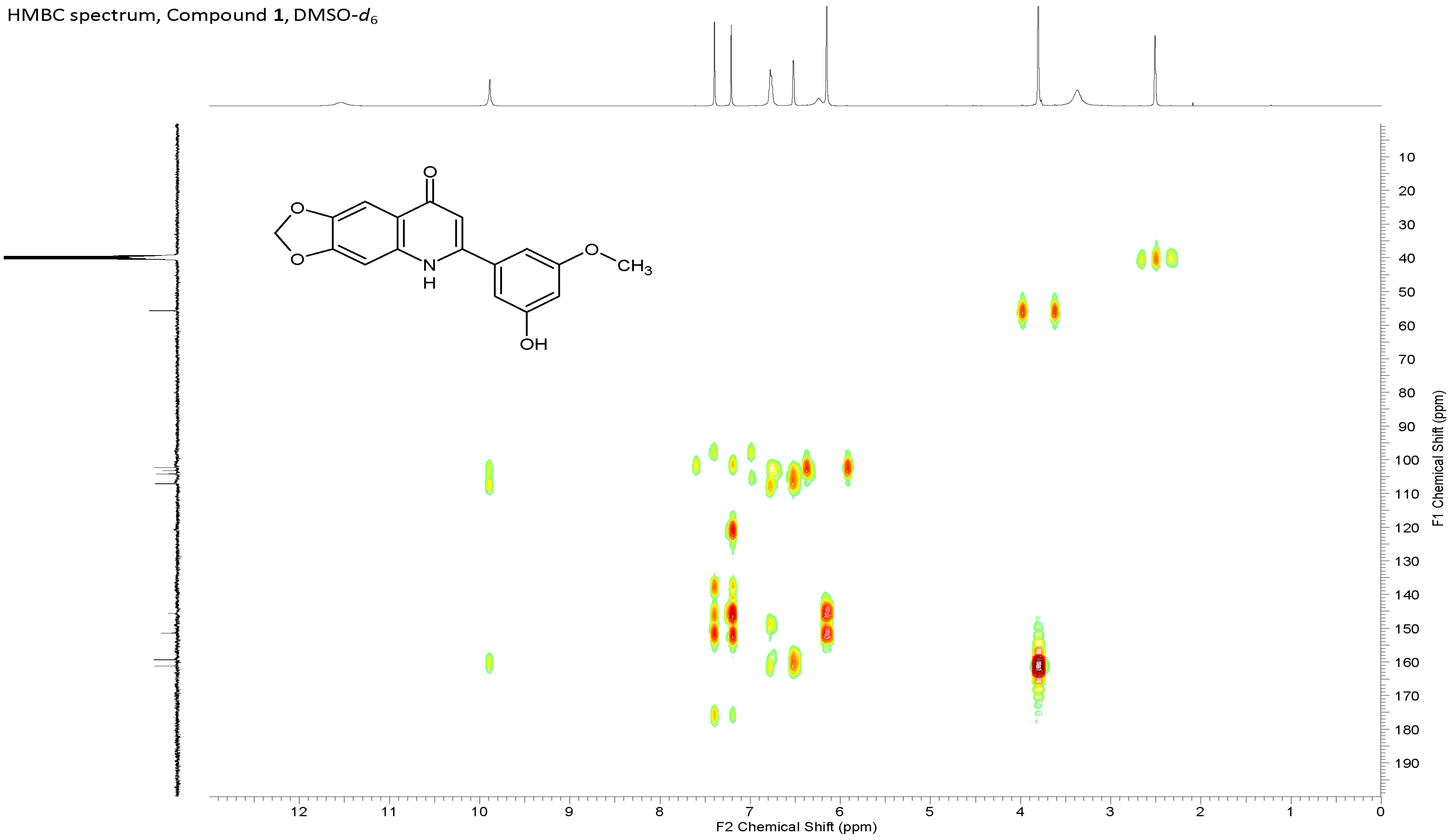

➢ *NMR Spectra of Compound **2***

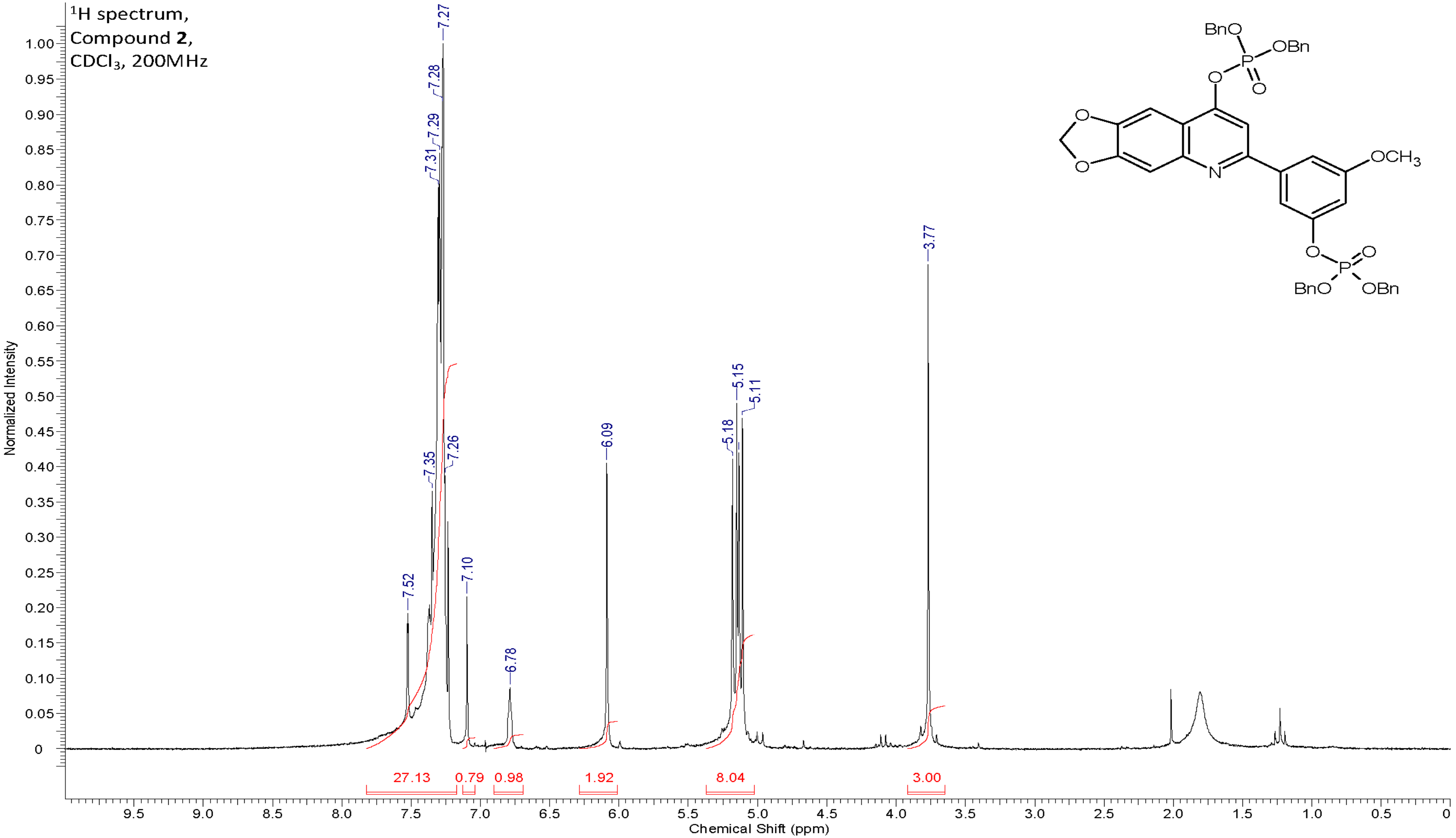

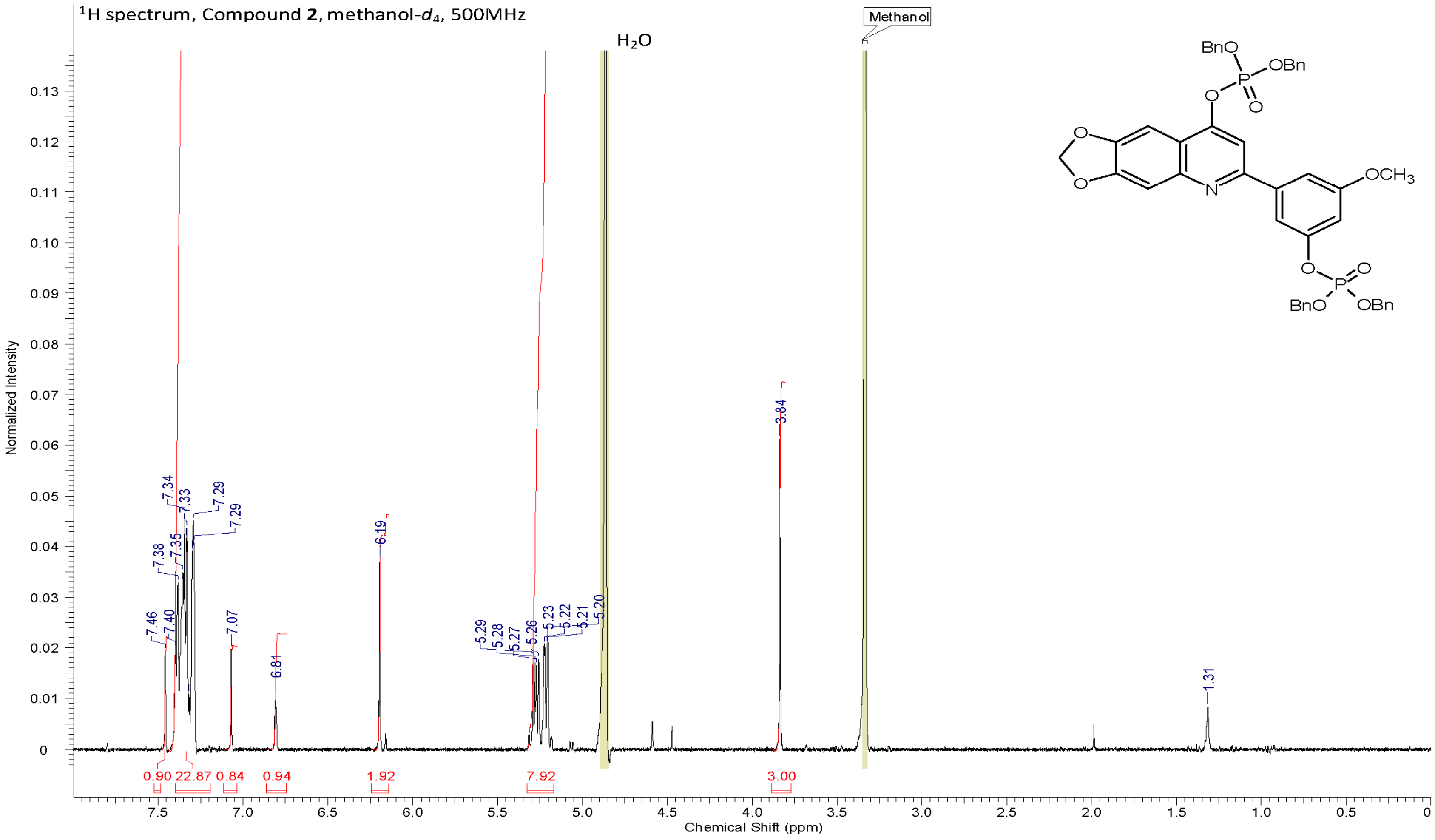

1H spectrum, Compound 2, methanol-d4, 500MHz
H2O
Methanol
Normalized Intensity
Chemical Shift (ppm)
7.46
7.40
7.38
7.35
7.34
7.33
7.29
7.29
7.07
6.81
6.19
5.29
5.28
5.27
5.26
5.23
5.22
5.21
5.20
3.84
1.31
0.90
22.87
0.84
0.94
1.92
7.92
3.00
BnO
OBn
OCH3
N

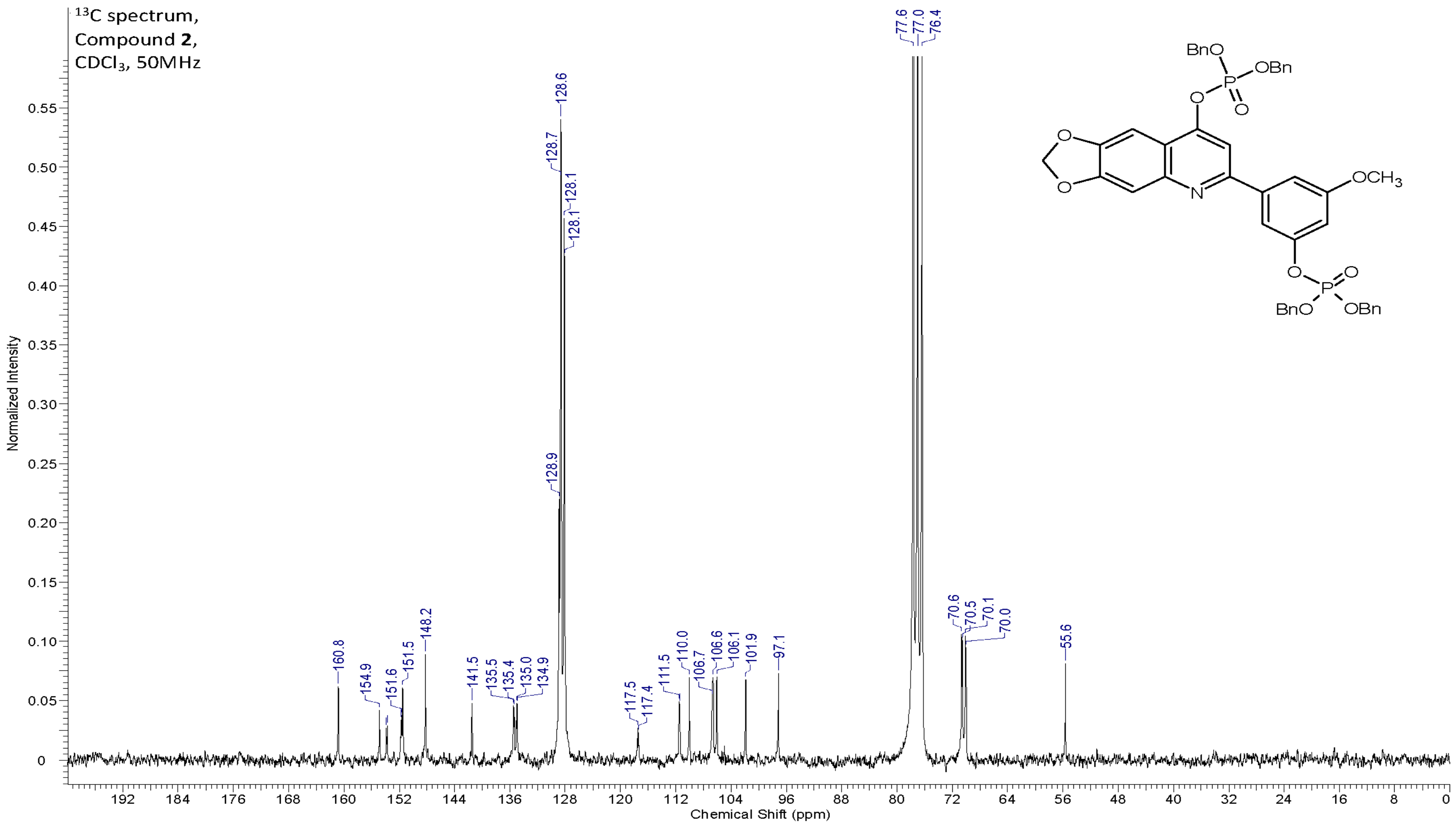

^{13}C spectrum,
Compound **2**,
$CDCl_3$, 50MHz
Normalized Intensity
Chemical Shift (ppm)
160.8
154.9
151.6
151.5
148.2
141.5
135.5
135.4
135.0
134.9
128.9
128.7
128.6
128.1
128.1
117.5
117.4
111.5
110.0
106.7
106.6
106.1
101.9
97.1
77.6
77.0
76.4
70.6
70.5
70.1
70.0
55.6
BnO
OBn
OCH3
N

HMQC spectrum,
Compound **2**, $CDCl_3$

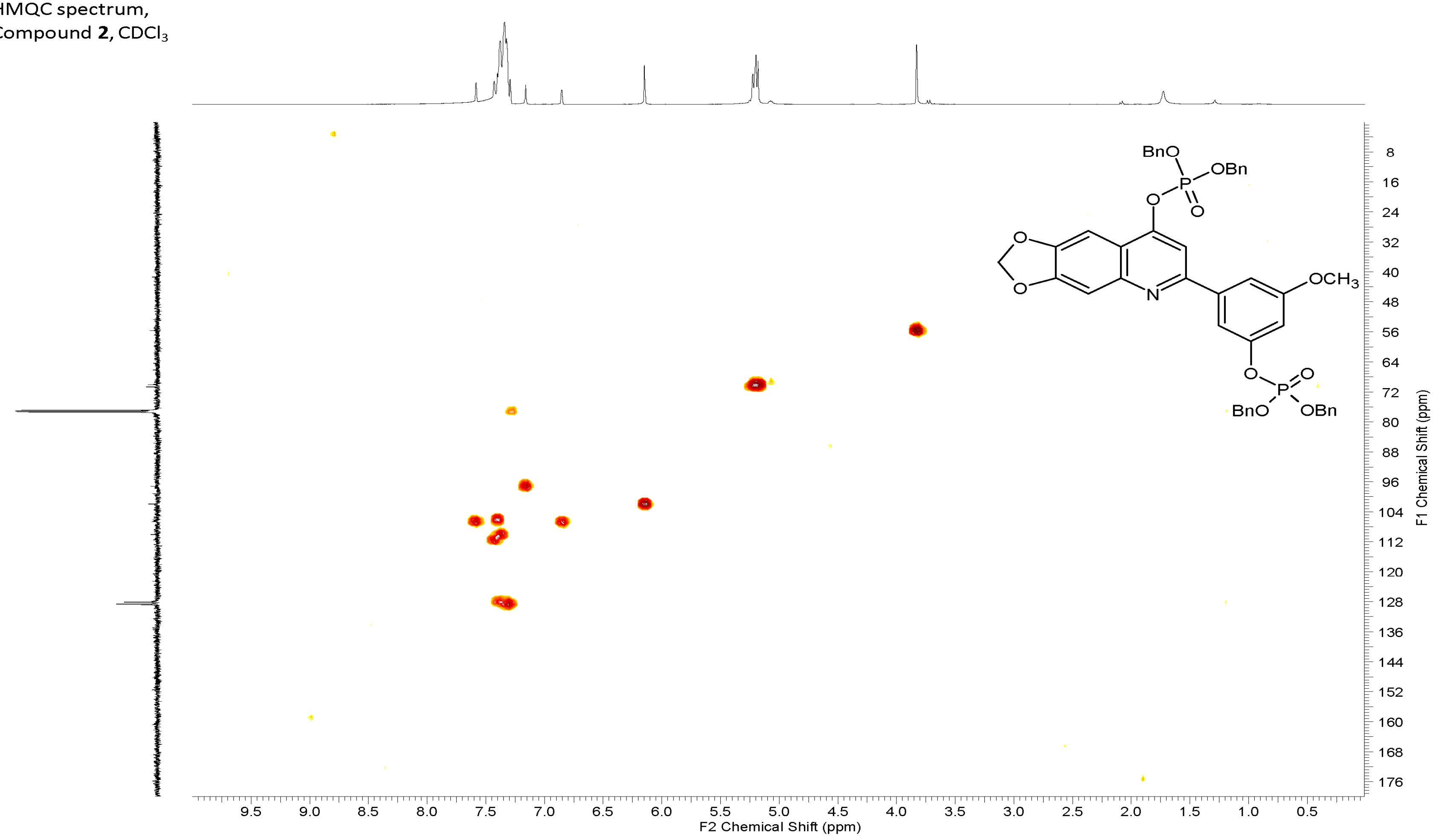

HMBC spectrum,
Compound **2**, $CDCl_3$

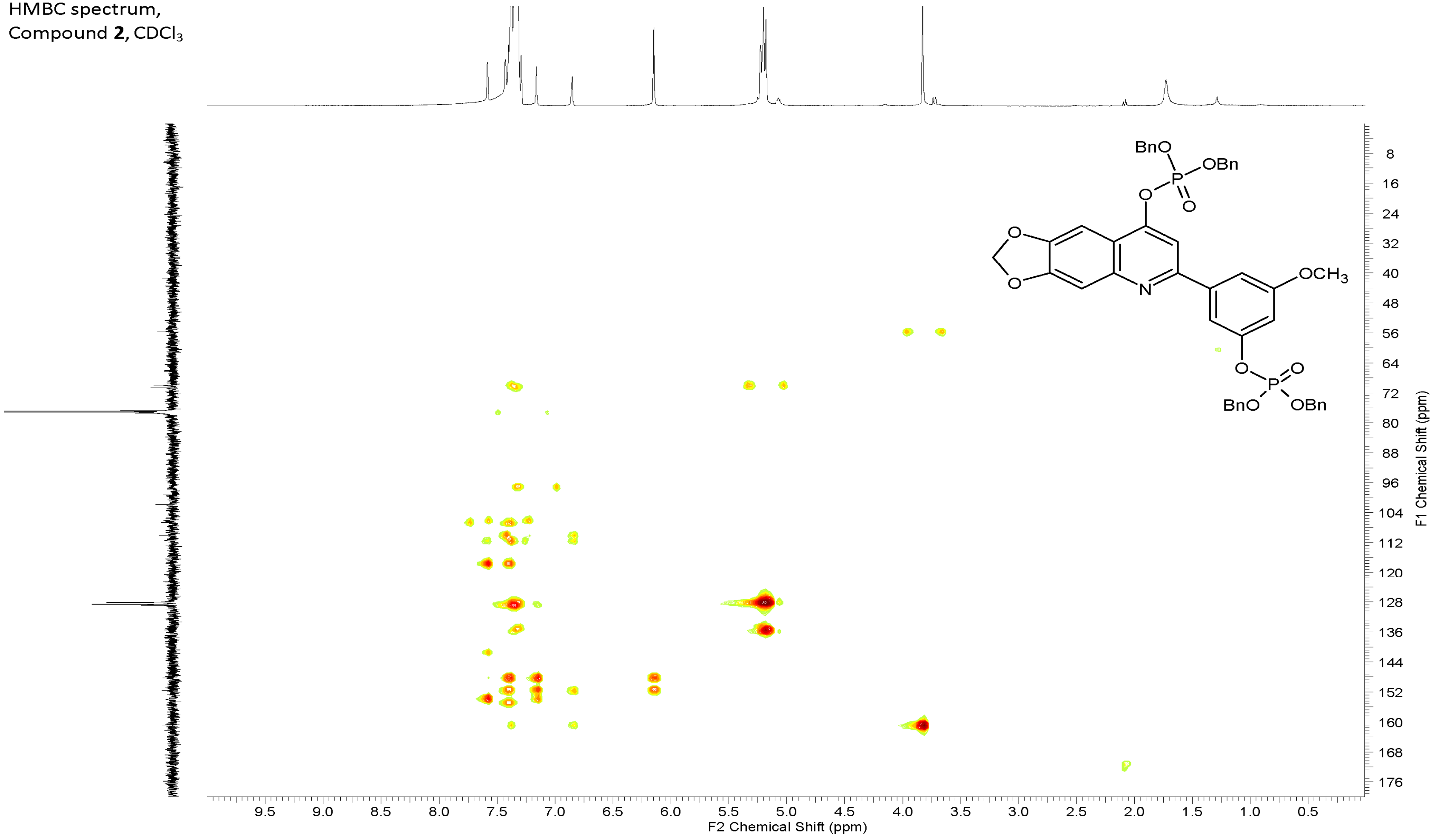

- *NMR Spectra of compound **3***

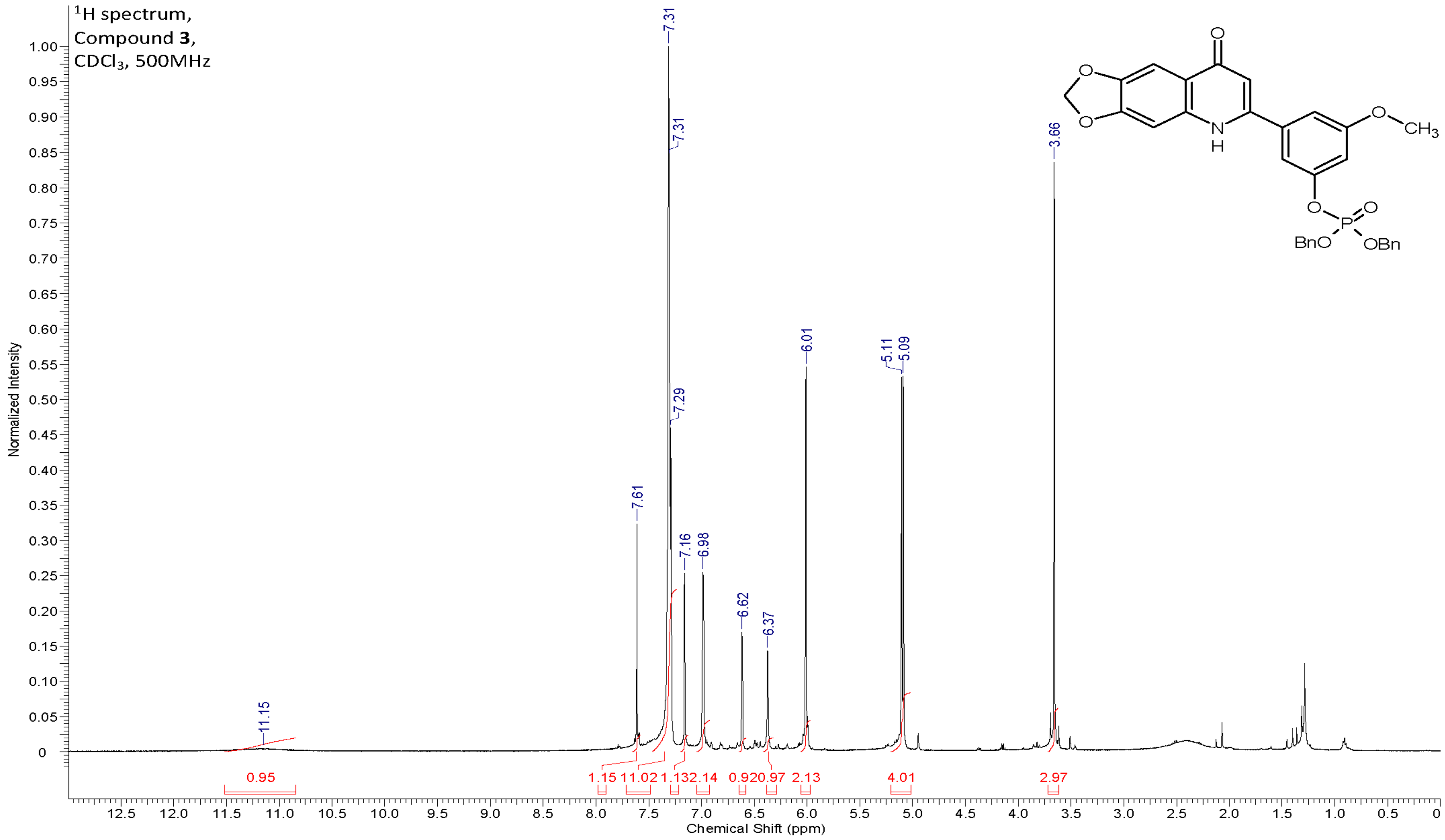

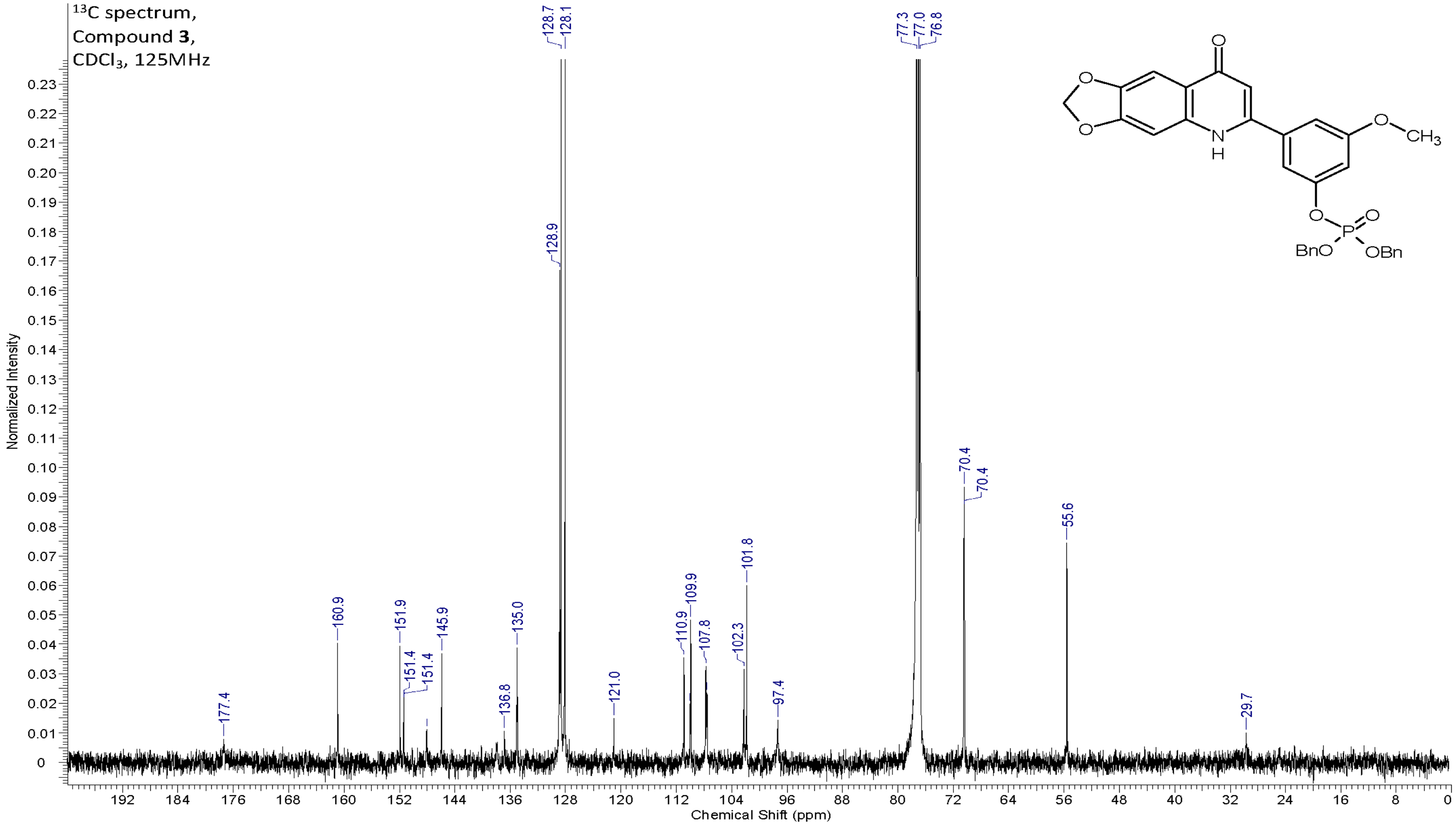

13C spectrum,
Compound 3,
CDCl3, 125MHz
Normalized Intensity
Chemical Shift (ppm)
177.4
160.9
151.9
151.4
151.4
145.9
136.8
135.0
128.9
128.7
128.1
121.0
110.9
109.9
107.8
102.3
101.8
97.4
77.3
77.0
76.8
70.4
70.4
55.6
29.7
O
N
H
O
O
O
CH3
O
P
O
BnO
OBn

HMQC spectrum,
Compound **3**, $CDCl_3$

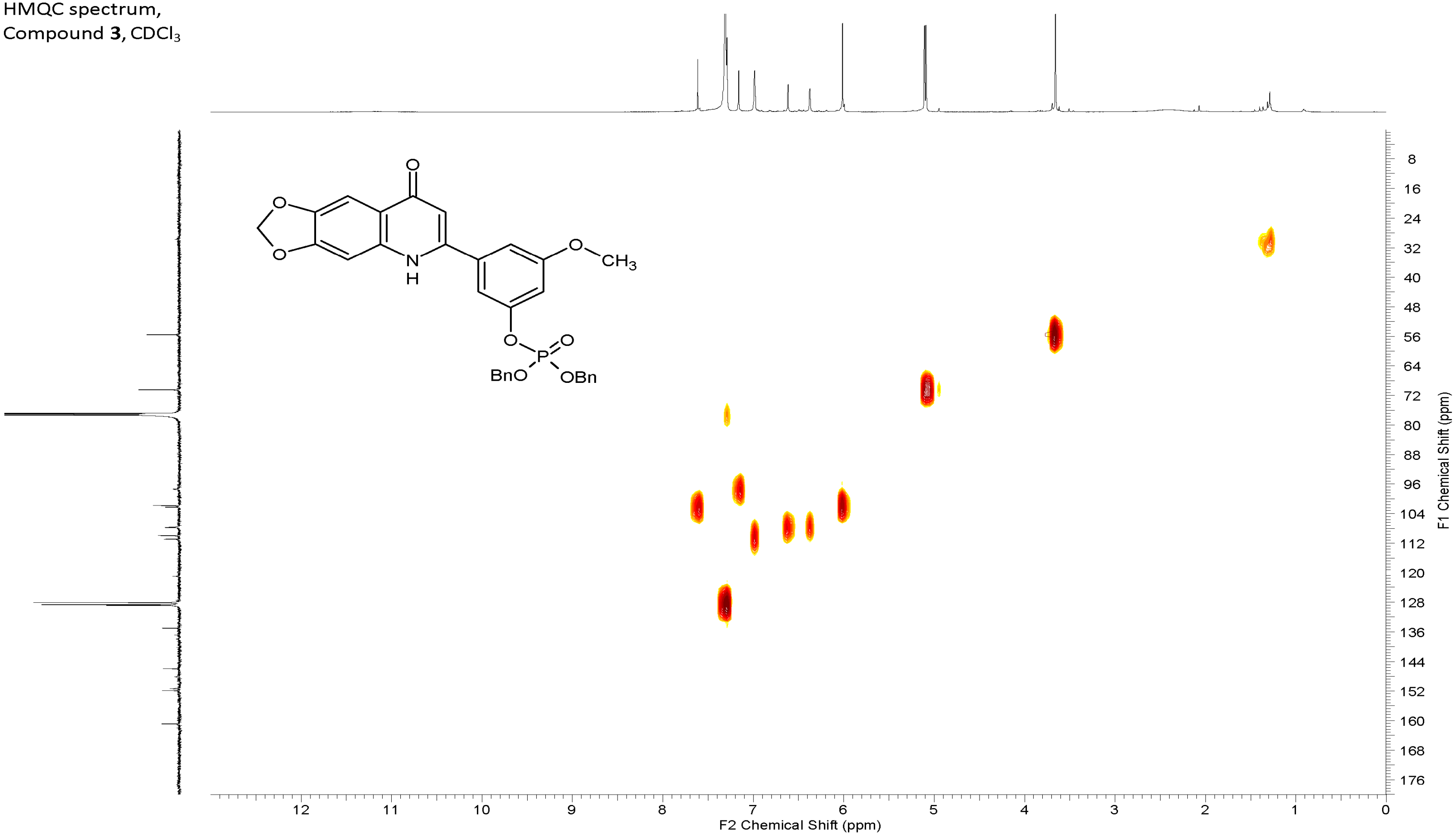

HMBC spectrum, Compound **3**, $CDCl_3$

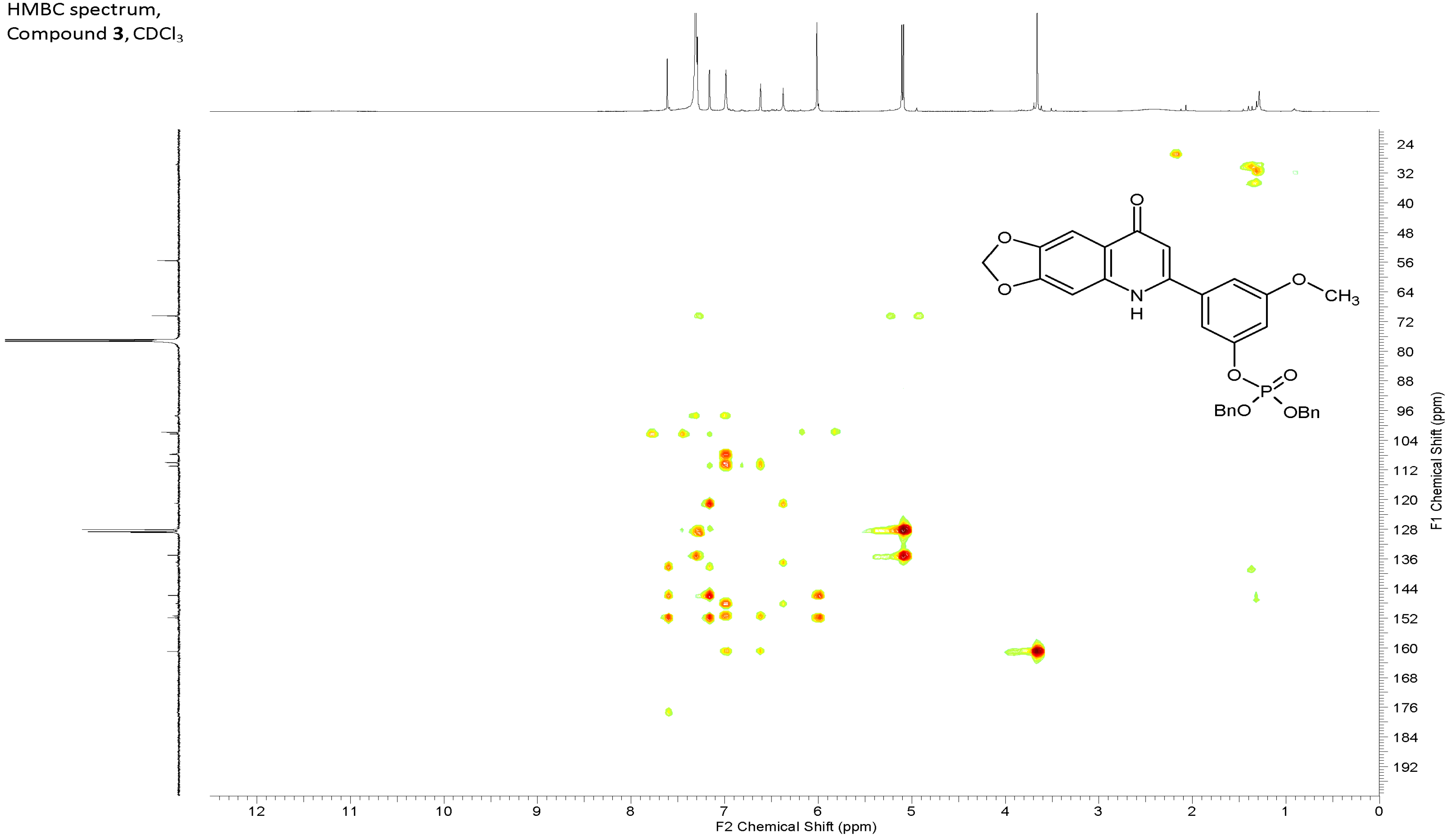

➢ *NMR Spectra of Compound **4***

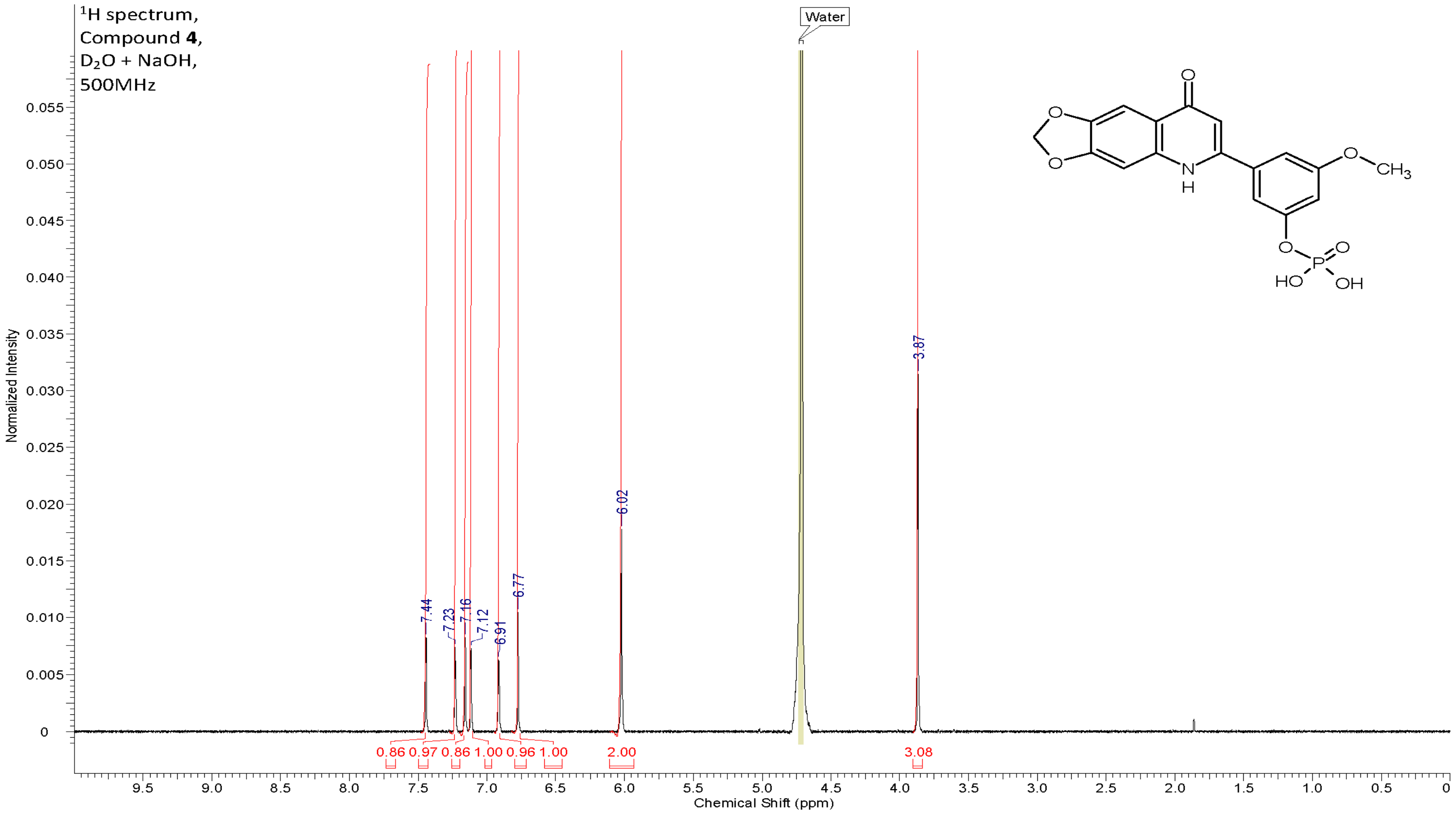

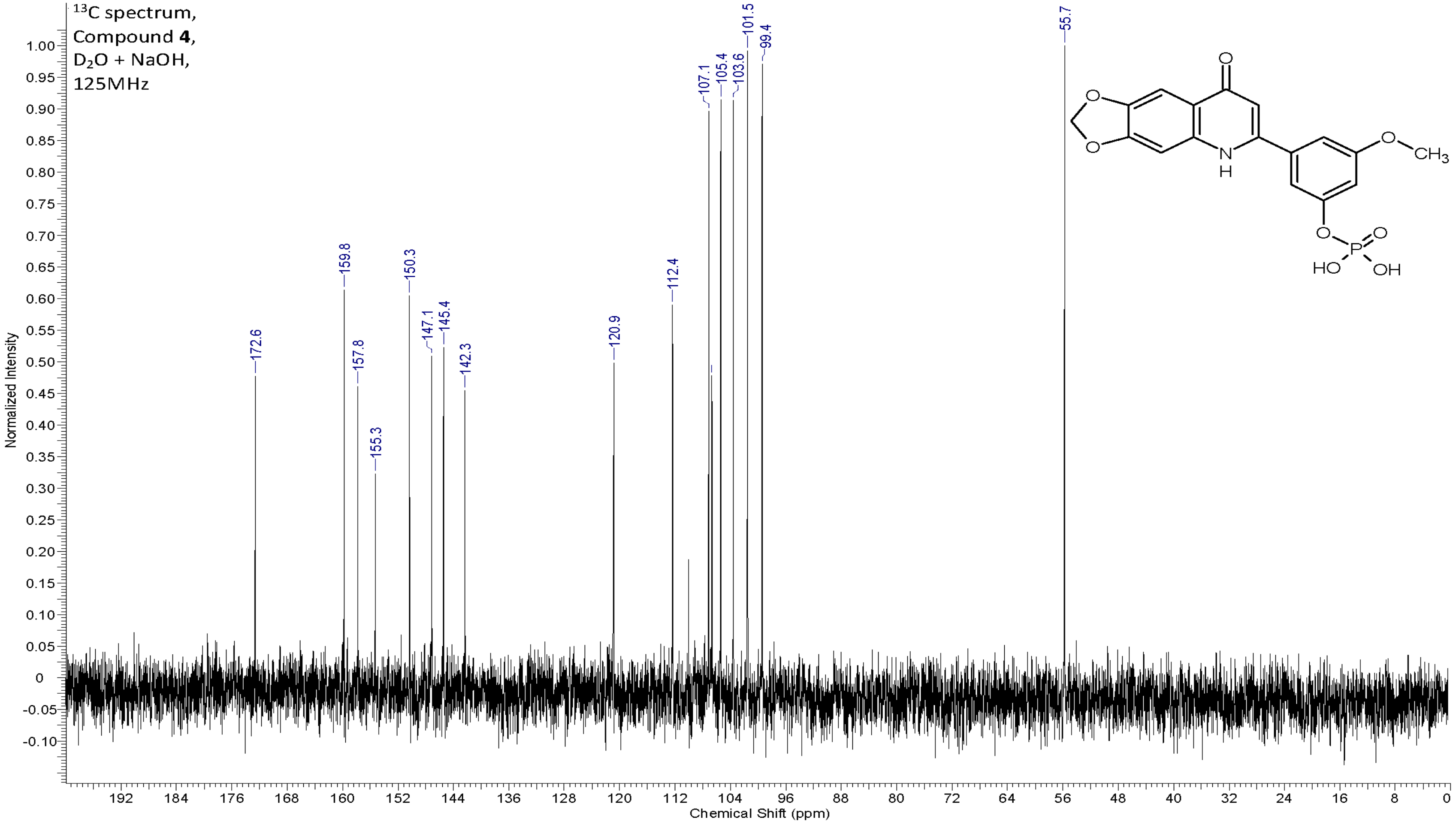

^{13}C spectrum,
Compound **4**,
D_2O + NaOH,
125MHz
172.6
159.8
157.8
155.3
150.3
147.1
145.4
142.3
120.9
112.4
107.1
105.4
103.6
101.5
99.4
55.7
Normalized Intensity
Chemical Shift (ppm)

HMQC spectrum,
Compound **4**,
D_2O + NaOH

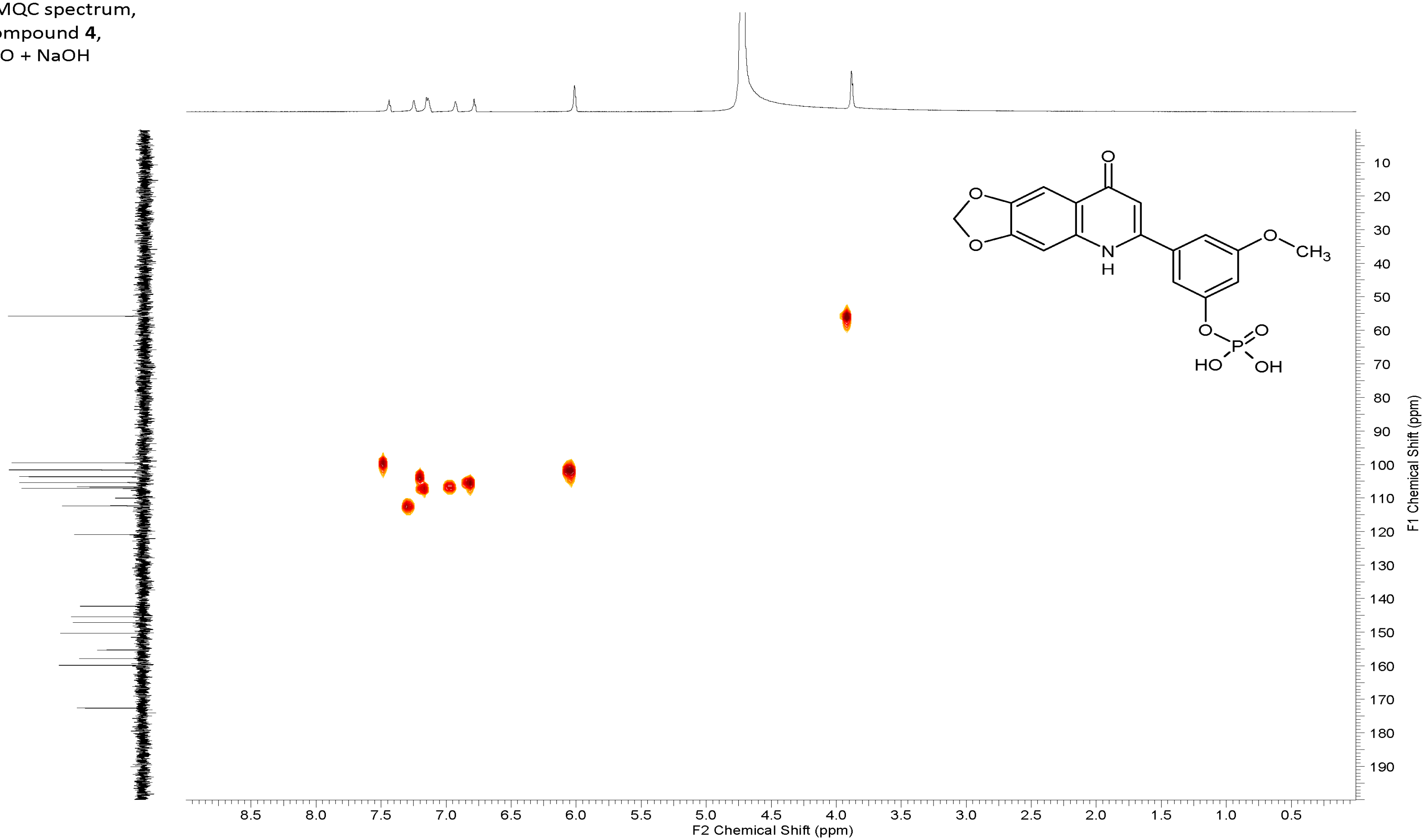

HMBC spectrum,
Compound **4**,
D_2O + NaOH

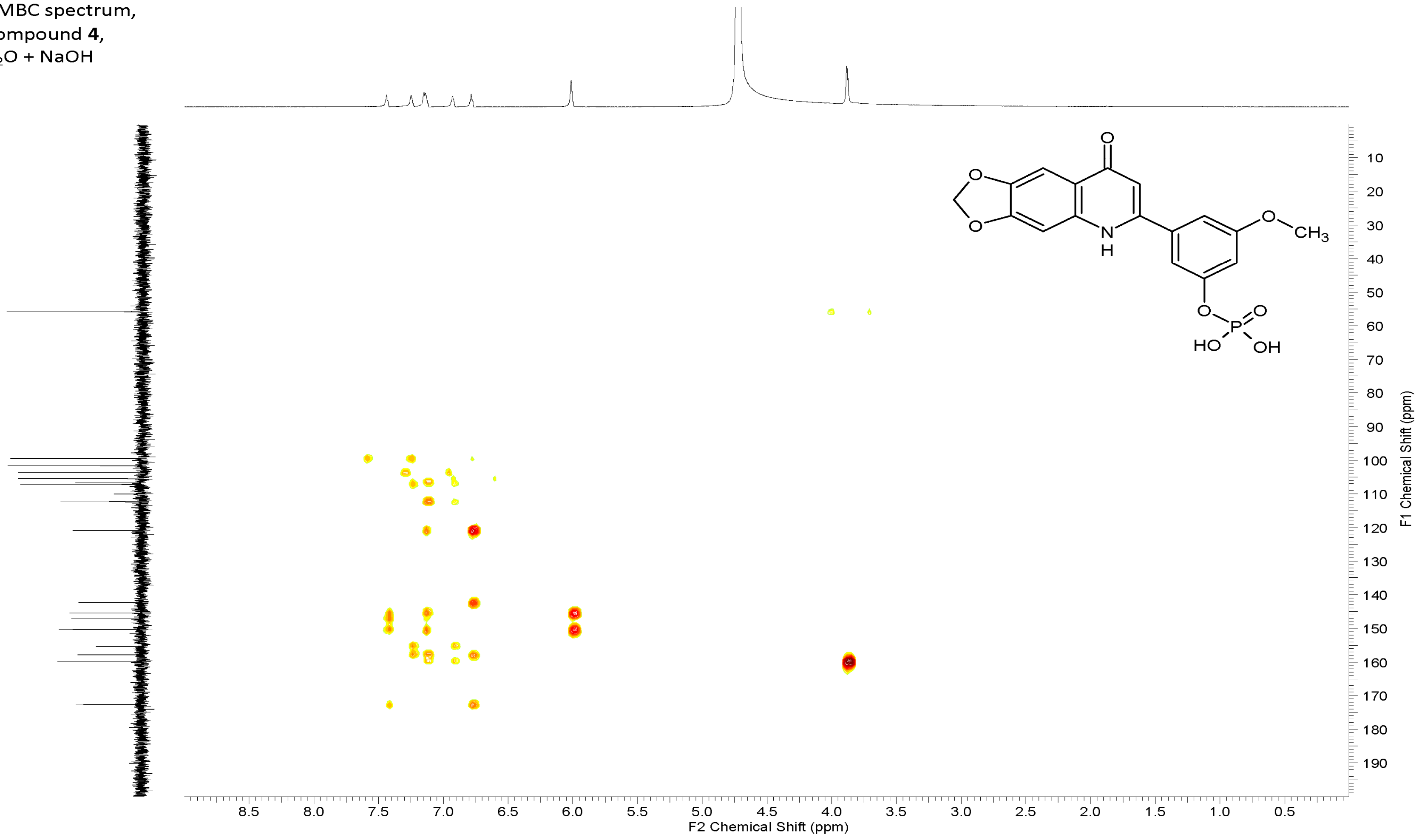

➢ *NMR Spectra of compound **9***

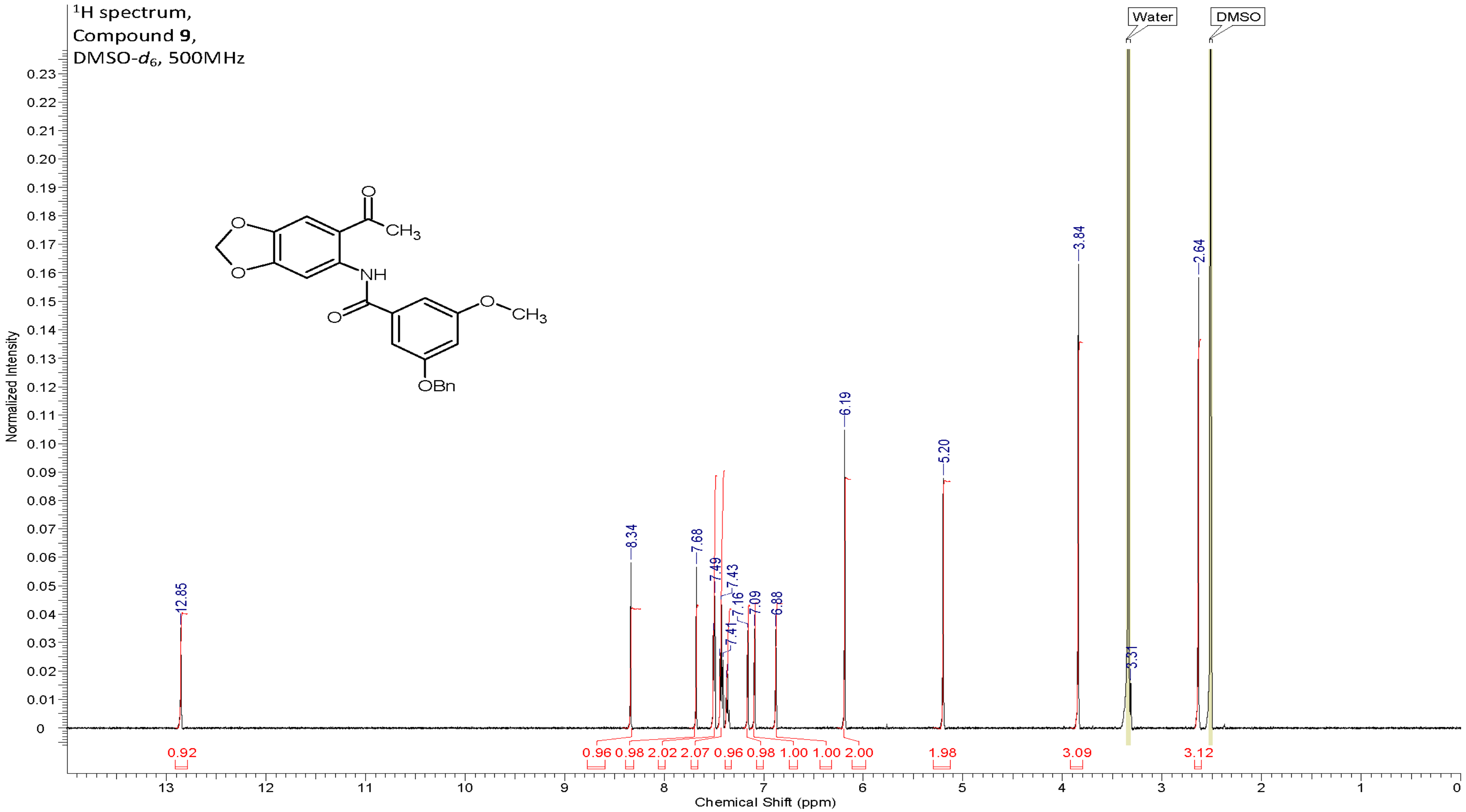

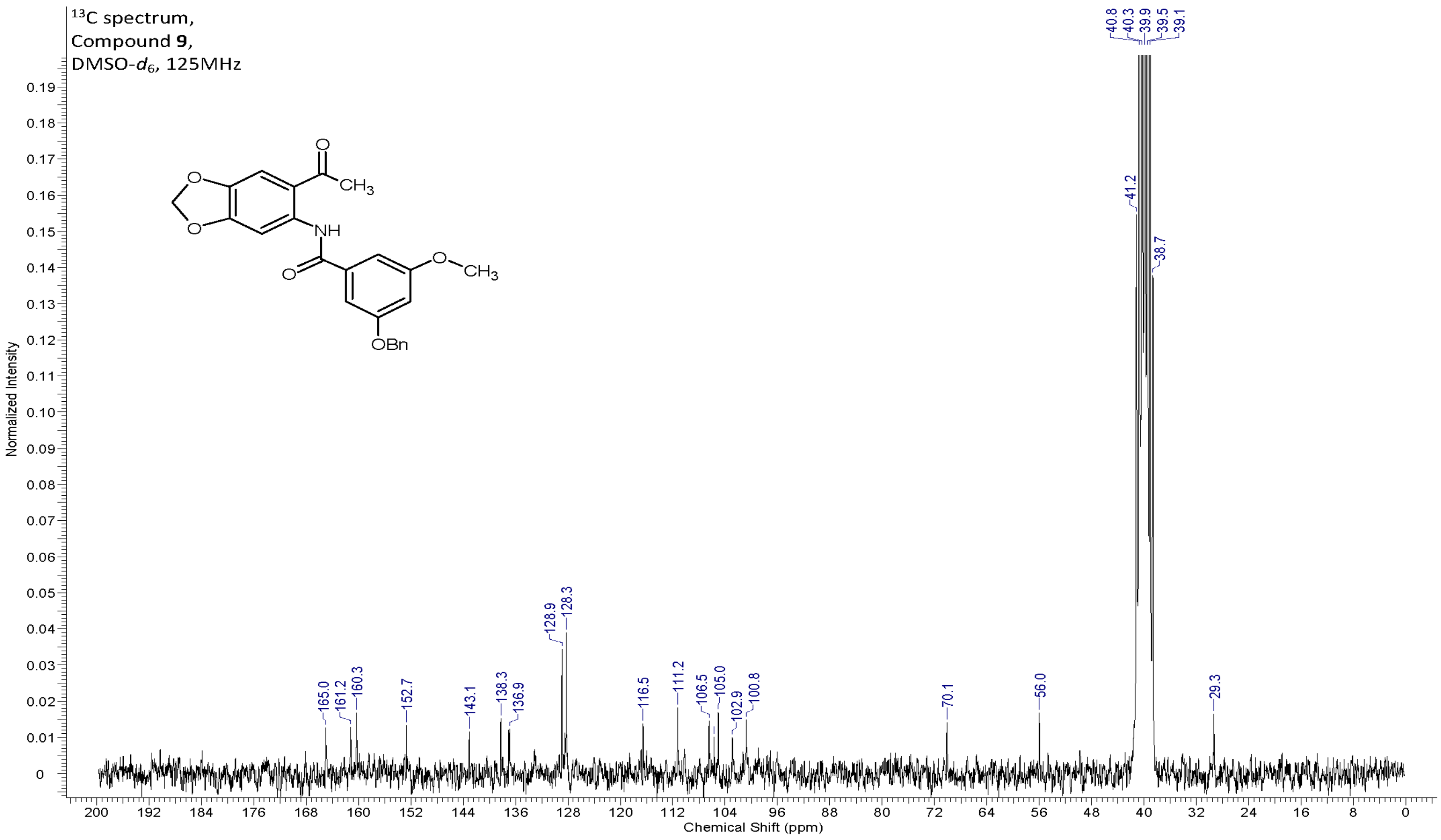

^{13}C spectrum,
Compound **9**,
DMSO-d_6, 125MHz
CH3
NH
O
CH3
OBn
Normalized Intensity
Chemical Shift (ppm)
165.0
161.2
160.3
152.7
143.1
138.3
136.9
128.9
128.3
116.5
111.2
106.5
105.0
102.9
100.8
70.1
56.0
41.2
40.8
40.3
39.9
39.5
39.1
38.7
29.3

HMQC spectrum,
Compound **9**,
DMSO-d_6

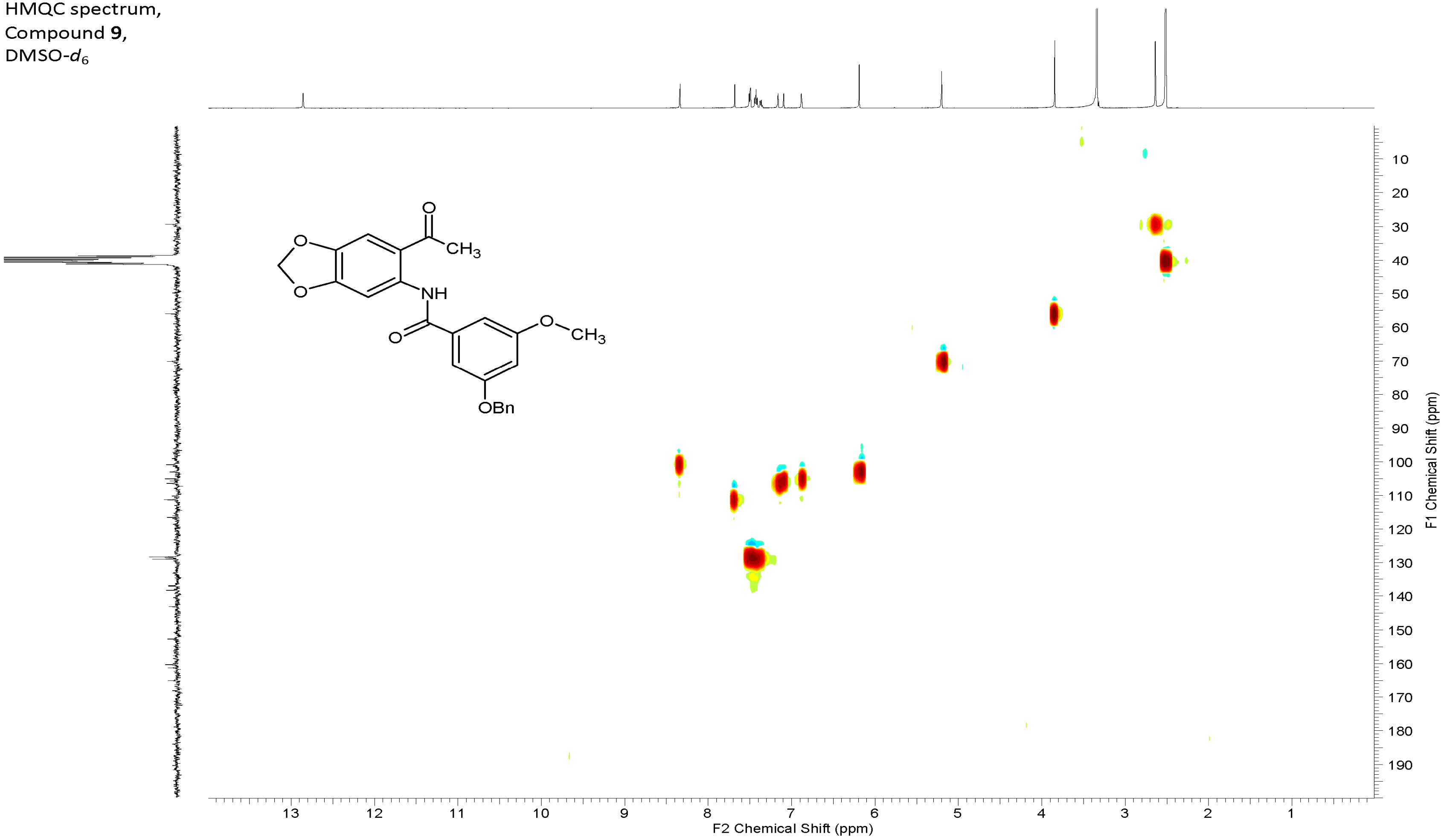

HMBC spectrum,
Compound **9**,
DMSO-d_6

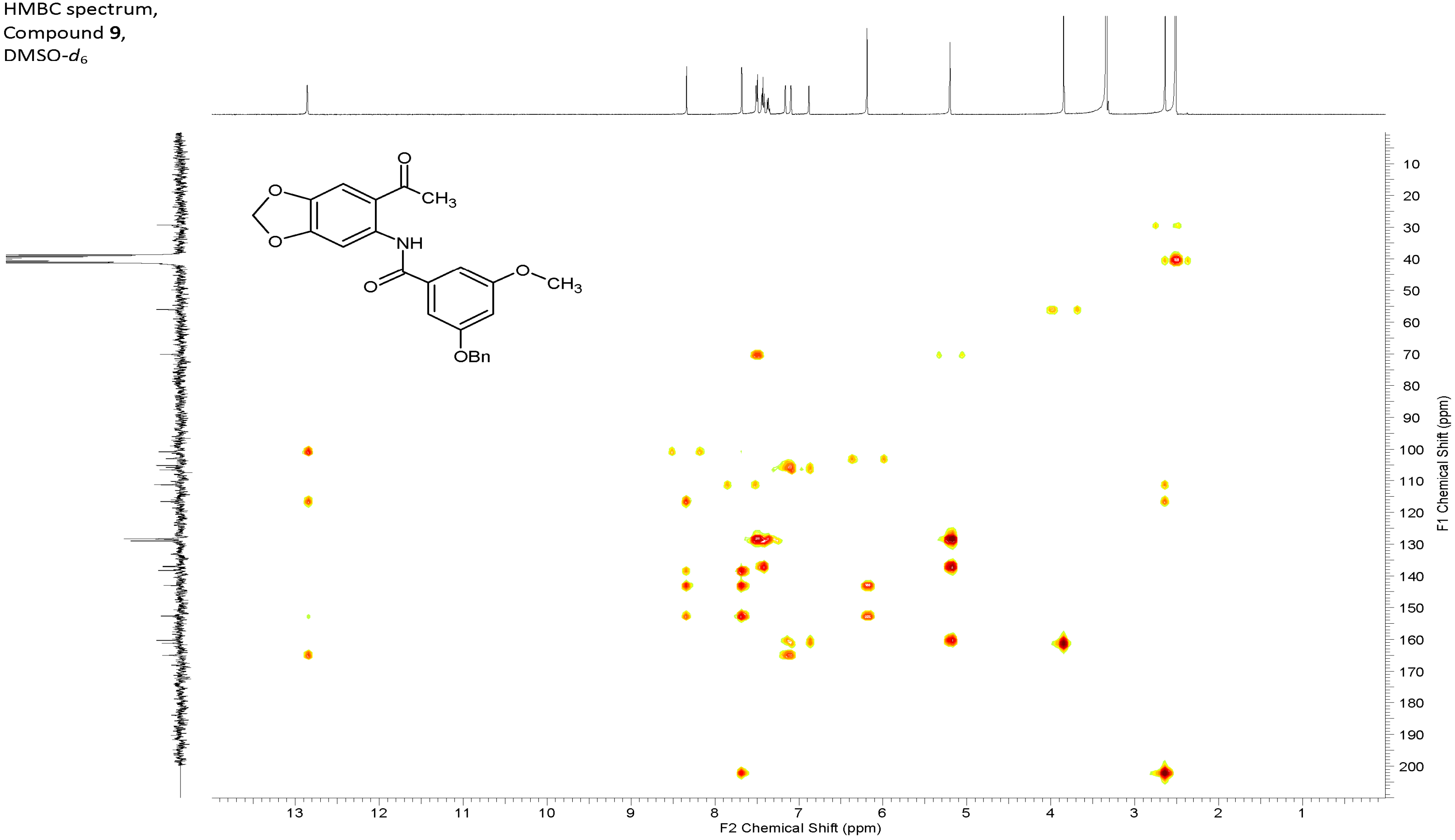

➢ *NMR Spectra of compound **10***

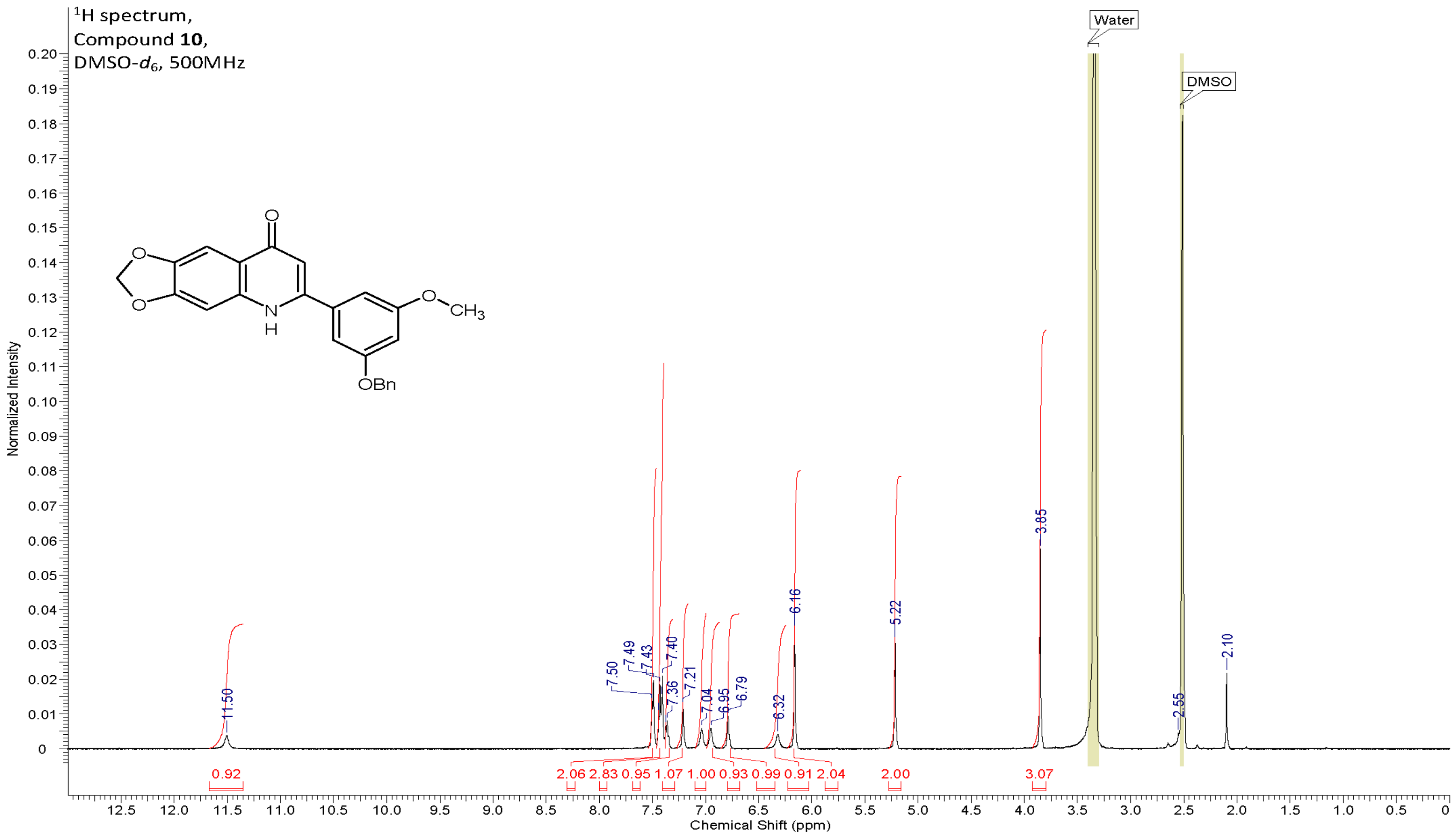

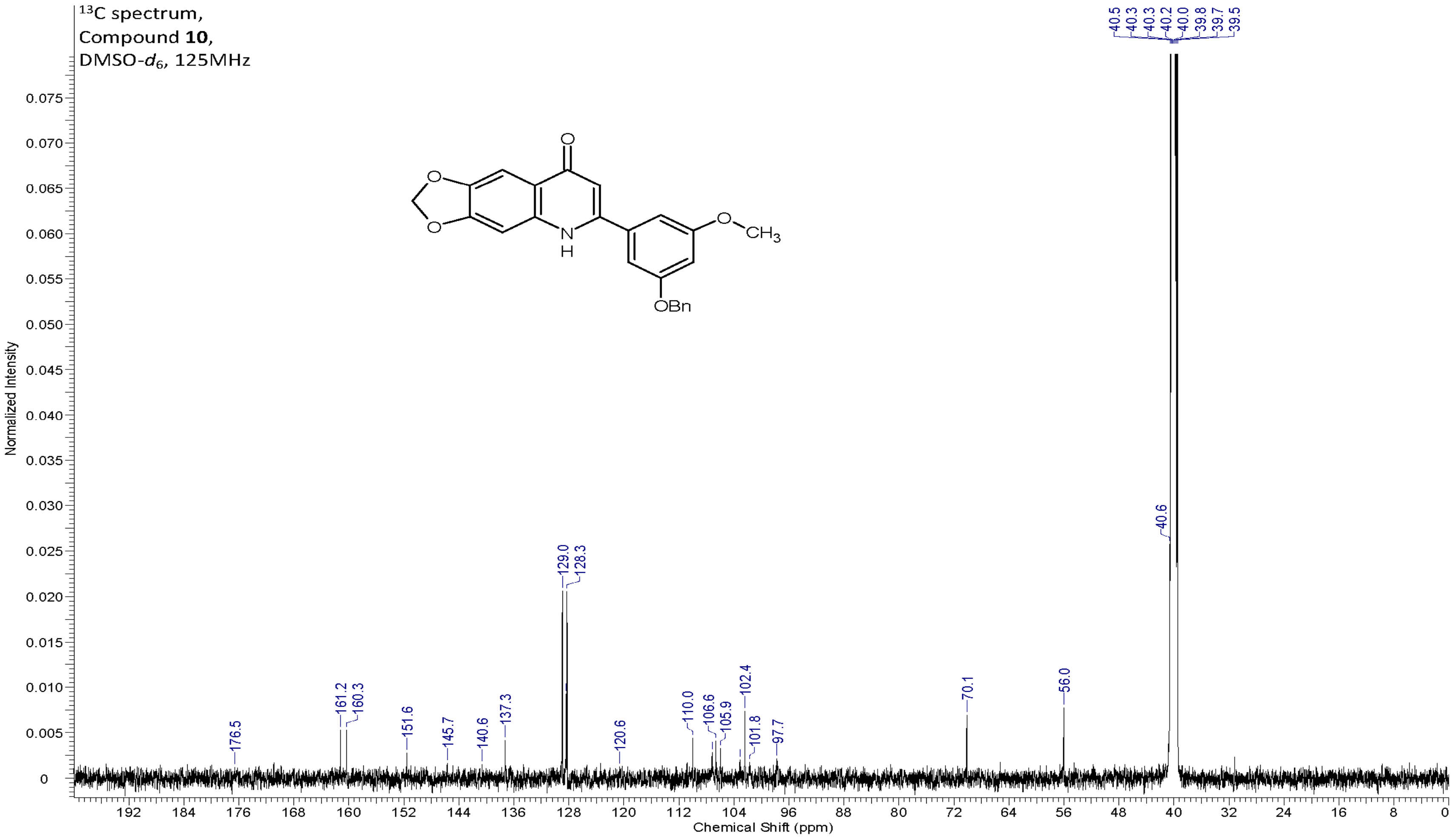

^{13}C spectrum,
Compound **10**,
DMSO-d_6, 125MHz
Normalized Intensity
Chemical Shift (ppm)
176.5
161.2
160.3
151.6
145.7
140.6
137.3
129.0
128.3
120.6
110.0
106.6
105.9
102.4
101.8
97.7
70.1
56.0
40.6
40.5
40.3
40.3
40.2
40.0
39.8
39.7
39.5
O
N
H
O
O
O
CH3
OBn

HMQC spectrum,
Compound **10**,
DMSO-d_6

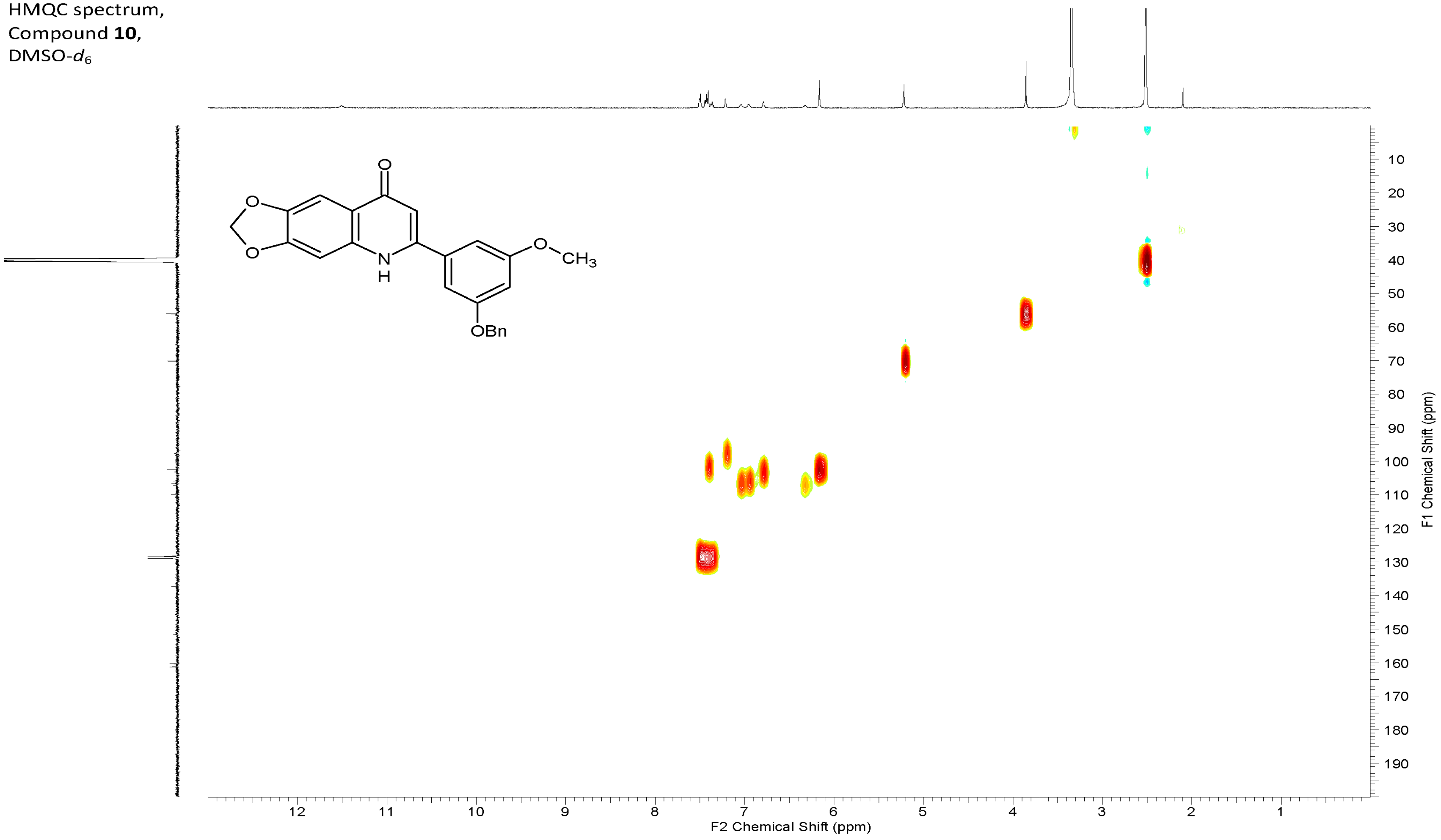

HMBC spectrum,
Compound **10**,
DMSO-d_6

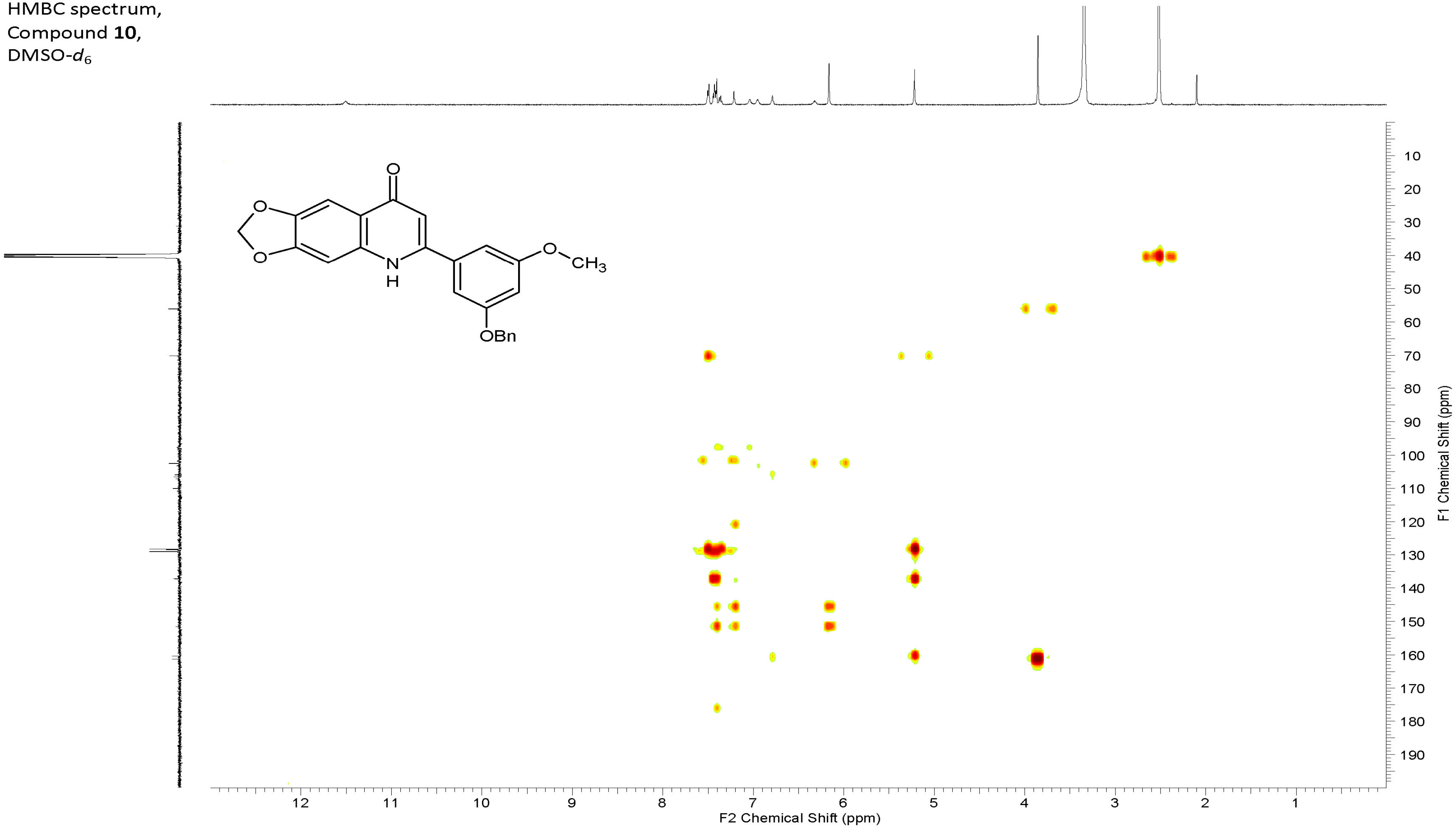

➢ *NMR Spectra of compound **11***

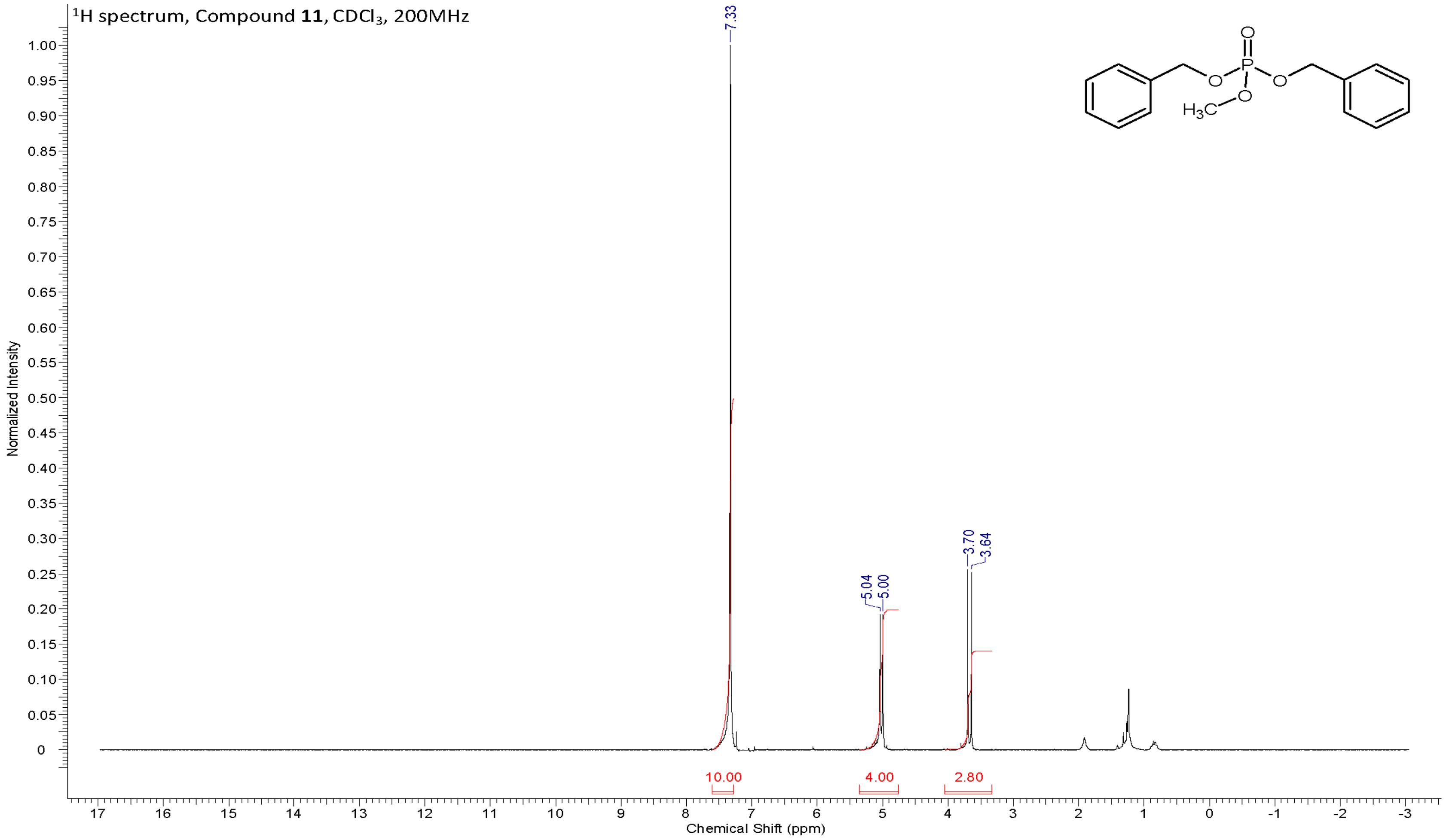

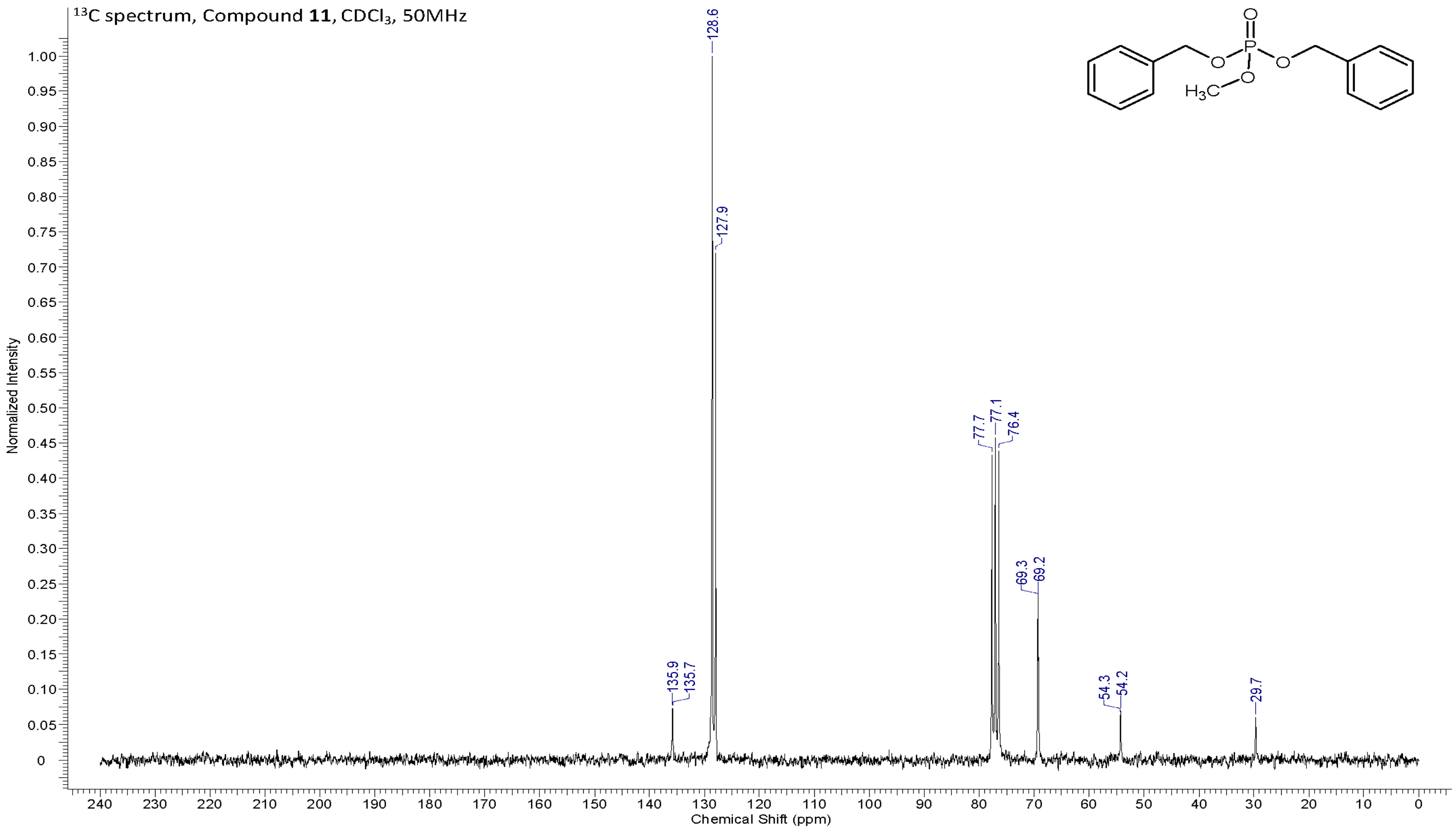

^{13}C spectrum, Compound **11**, CDCl$_3$, 50MHz
Normalized Intensity
Chemical Shift (ppm)
128.6
127.9
135.9
135.7
77.7
77.1
76.4
69.3
69.2
54.3
54.2
29.7
H3C

➢ *NMR Spectra of compound **12***

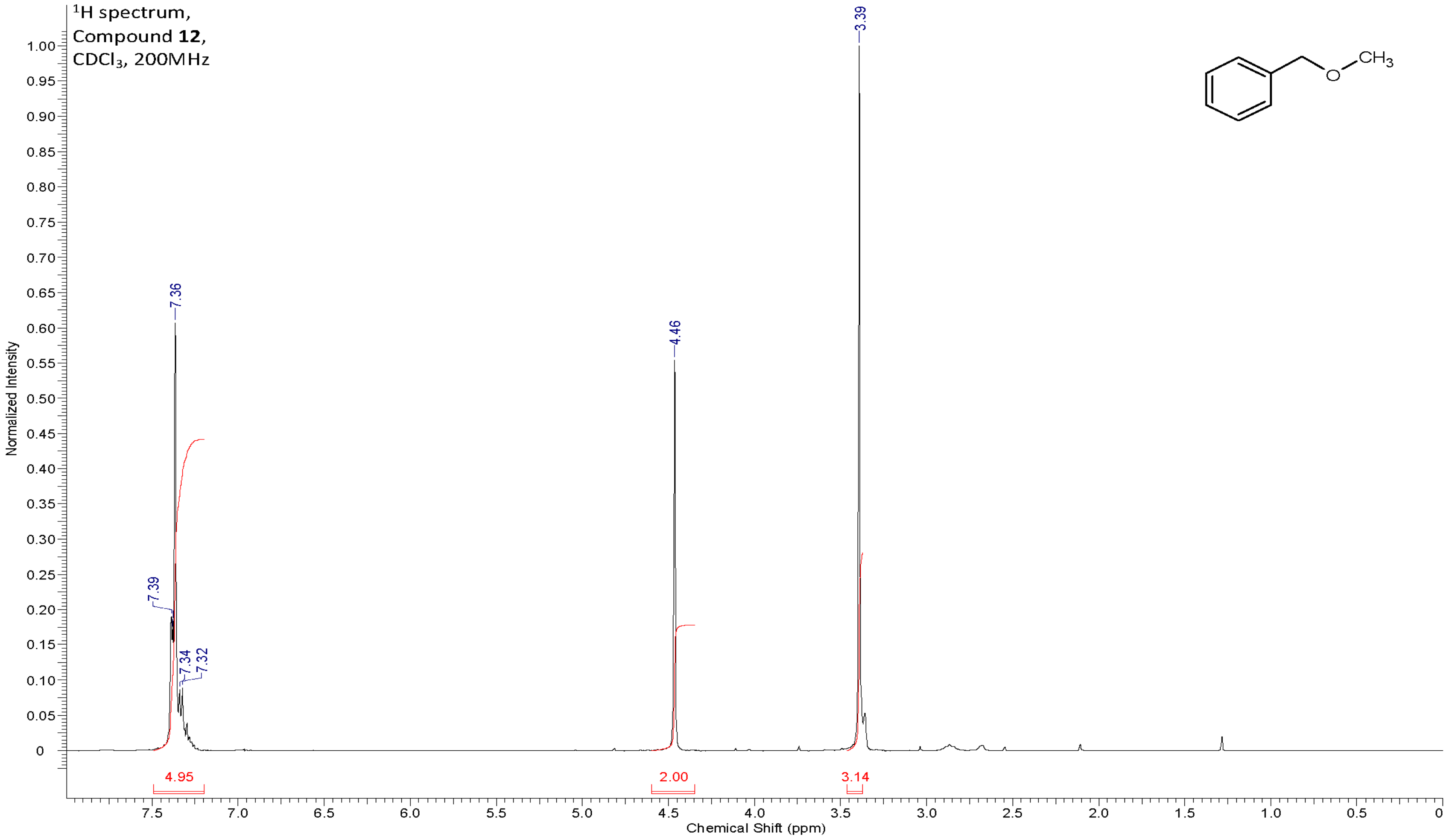

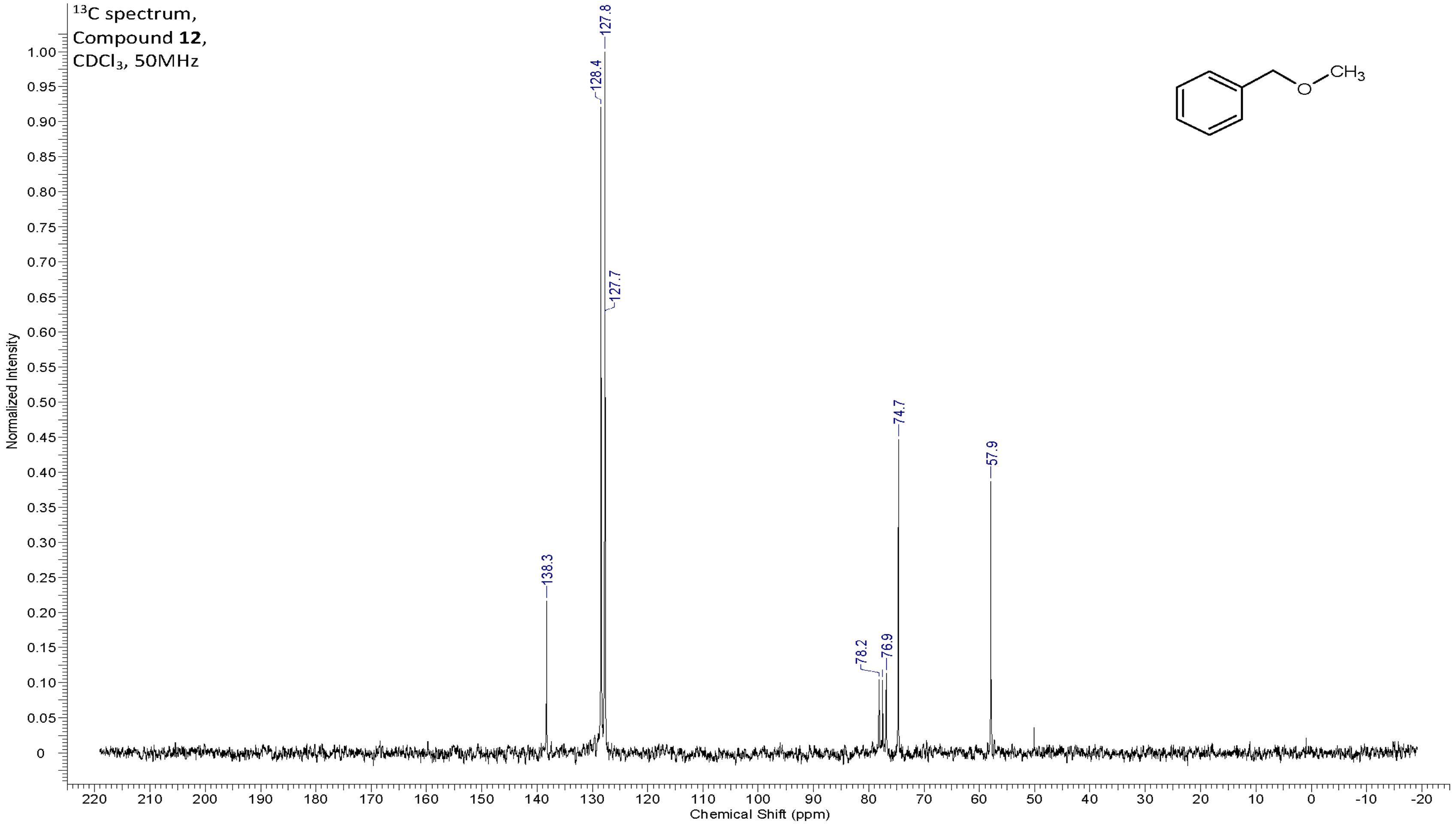

^{13}C spectrum,
Compound **12**,
$CDCl_3$, 50MHz
138.3
128.4
127.8
127.7
78.2
76.9
74.7
57.9
Normalized Intensity
Chemical Shift (ppm)
CH_3
O

- *NMR Spectra of compound* ***13***

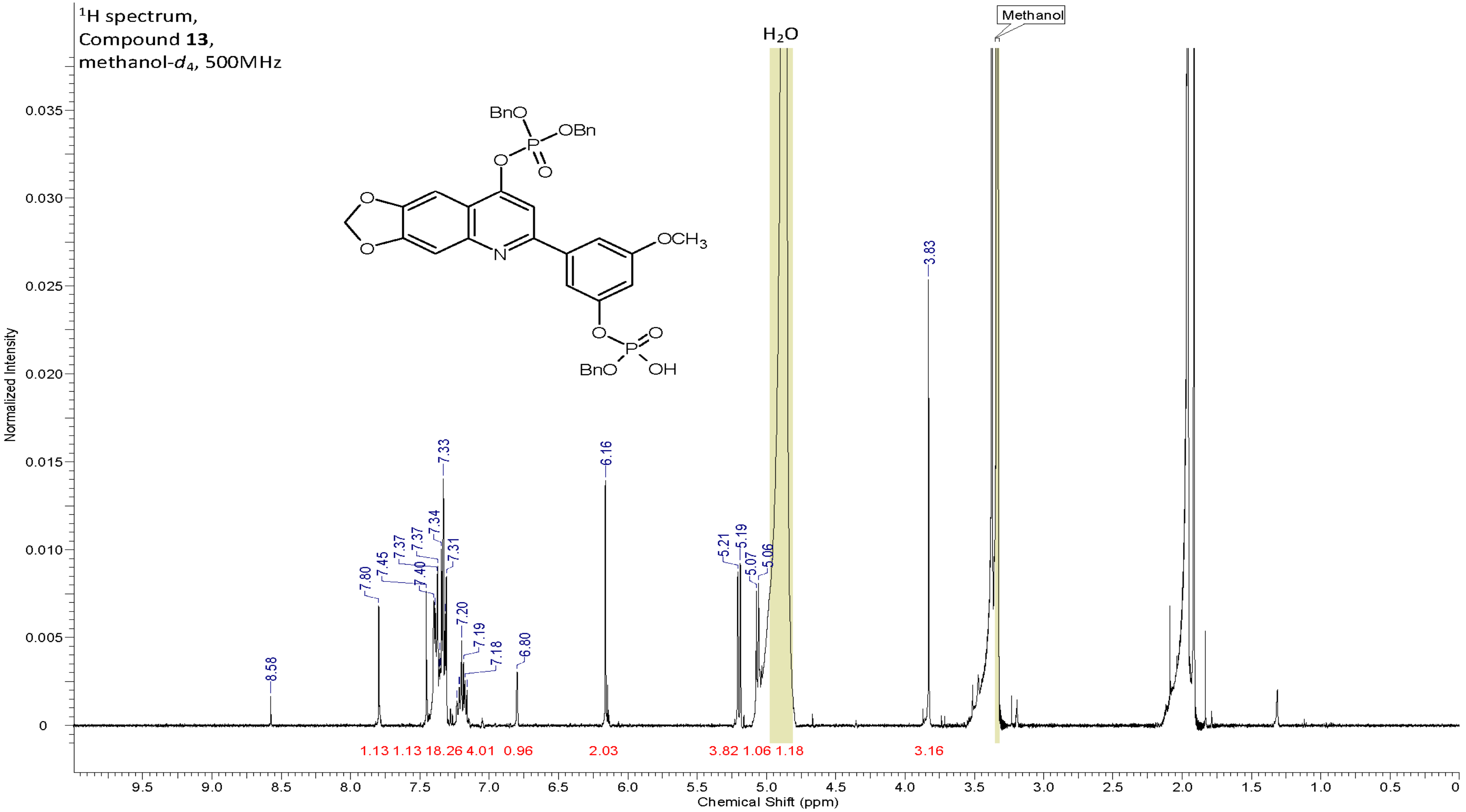

HMQC spectrum,
Compound **13**,
methanol-d_4

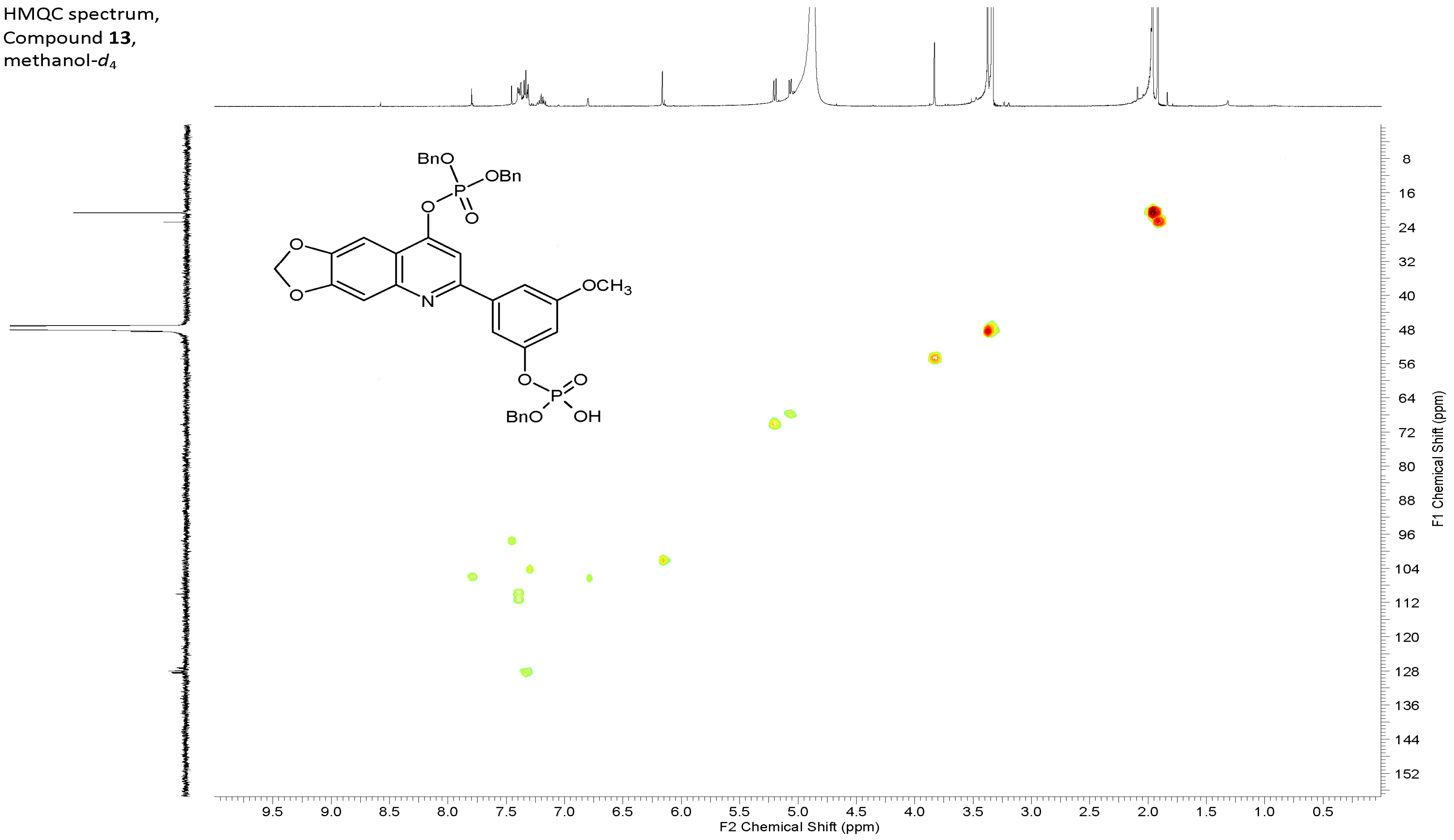

HMBC spectrum,
Compound **13**,
methanol-d_4

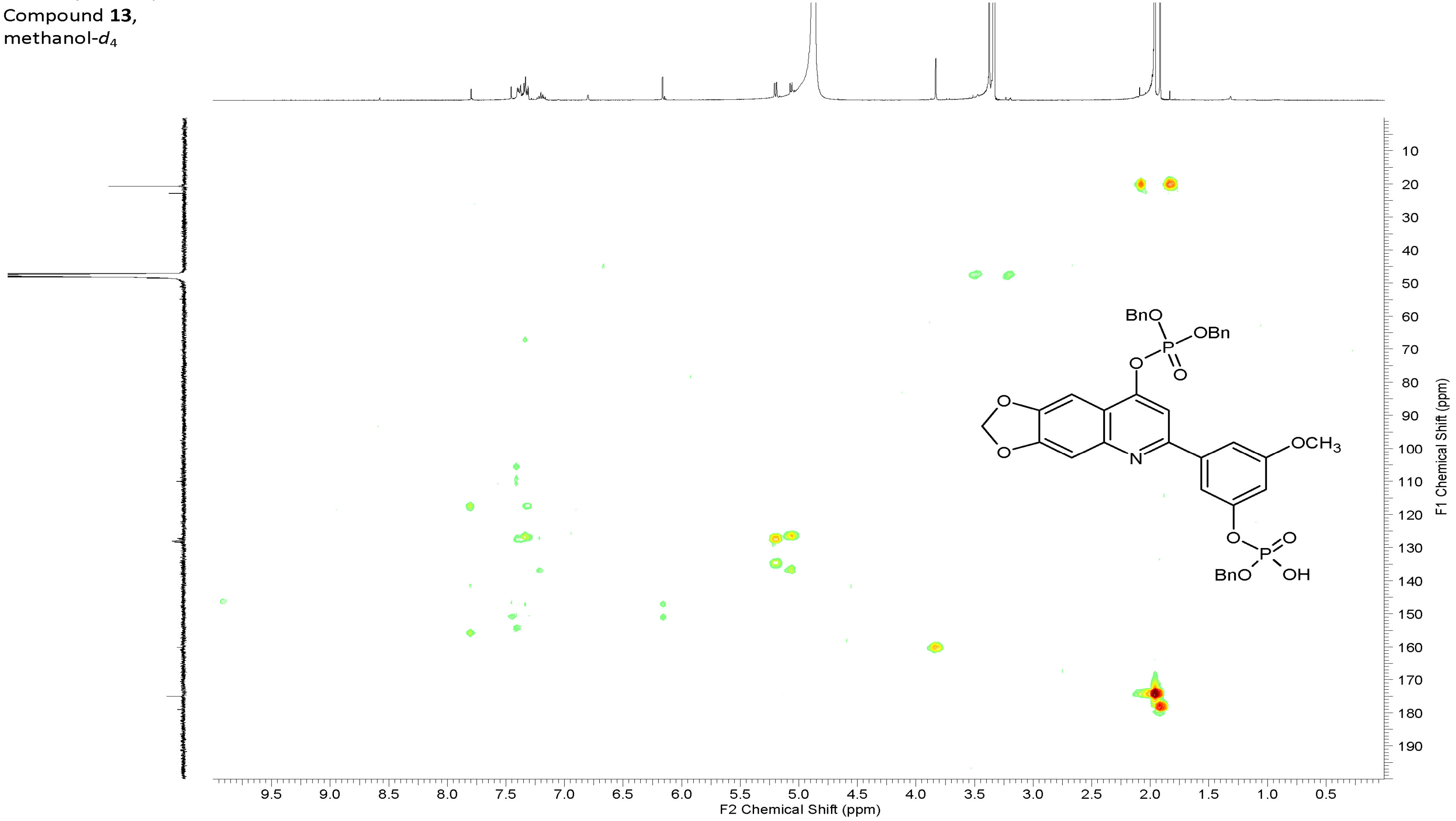

➢ *NMR Spectra of compound **14***

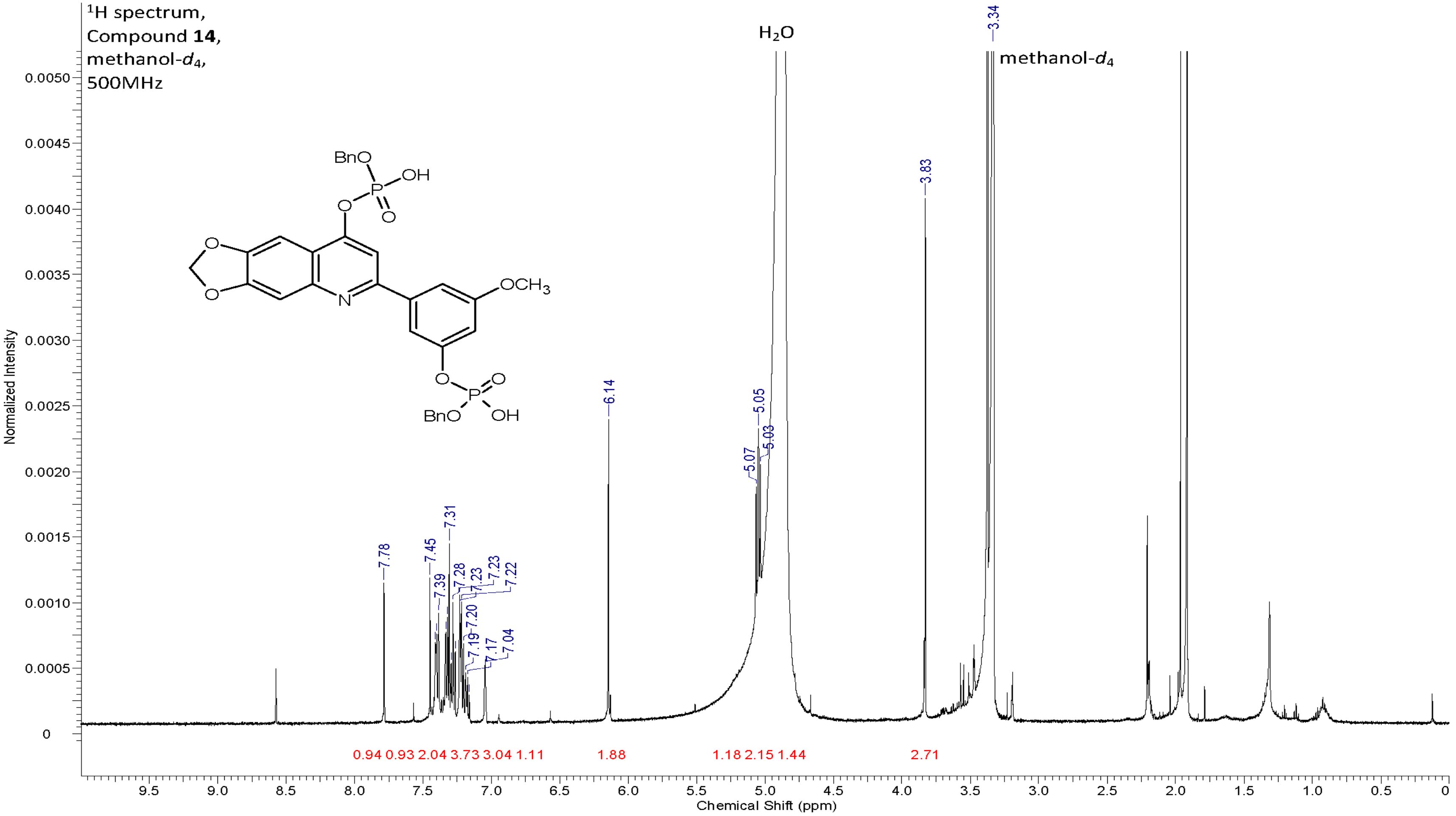

HMQC spectrum,
Compound **14**,
methanol-d_4

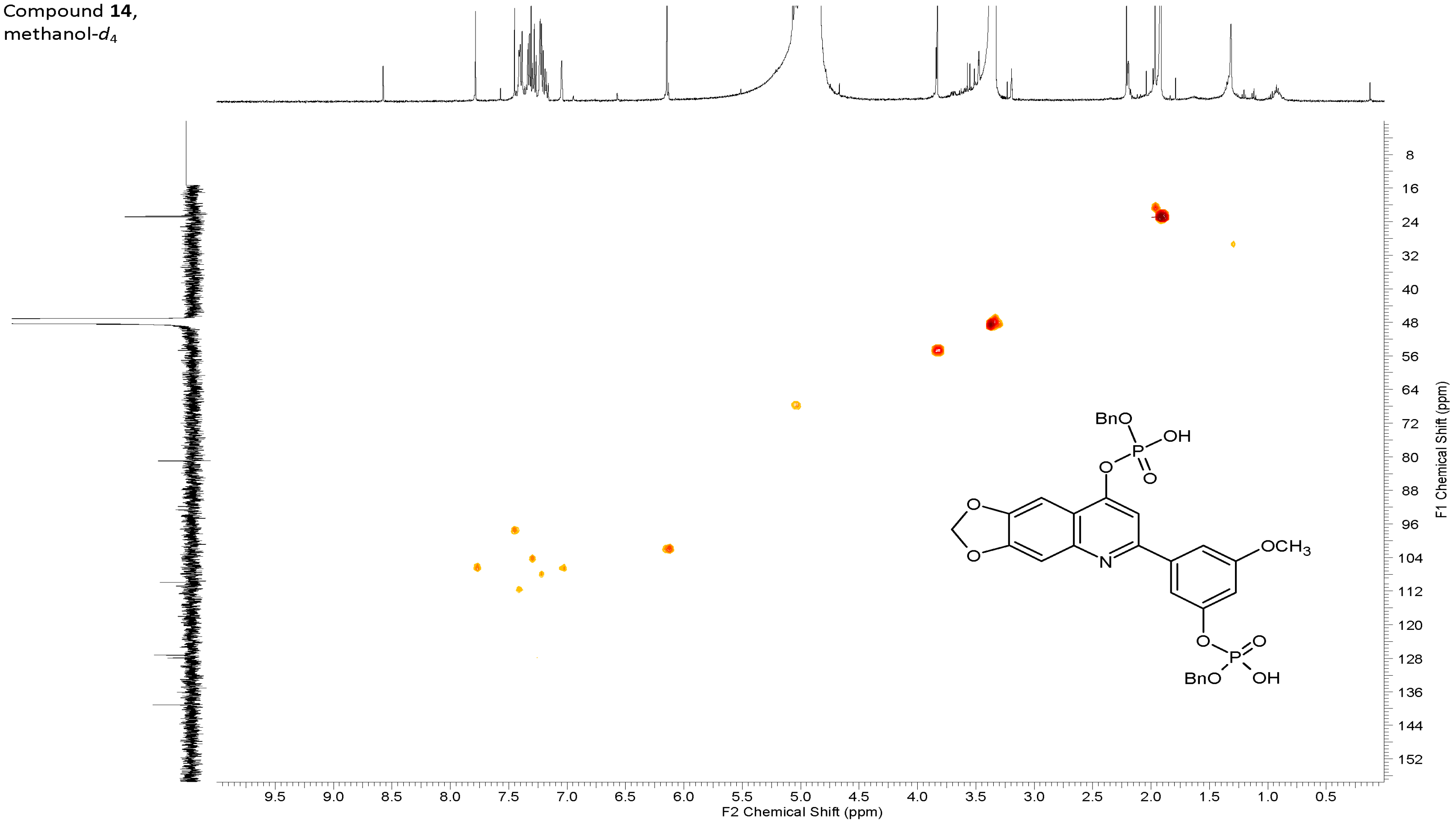

HMBC spectrum,
Compound **14**,
methanol-d_4

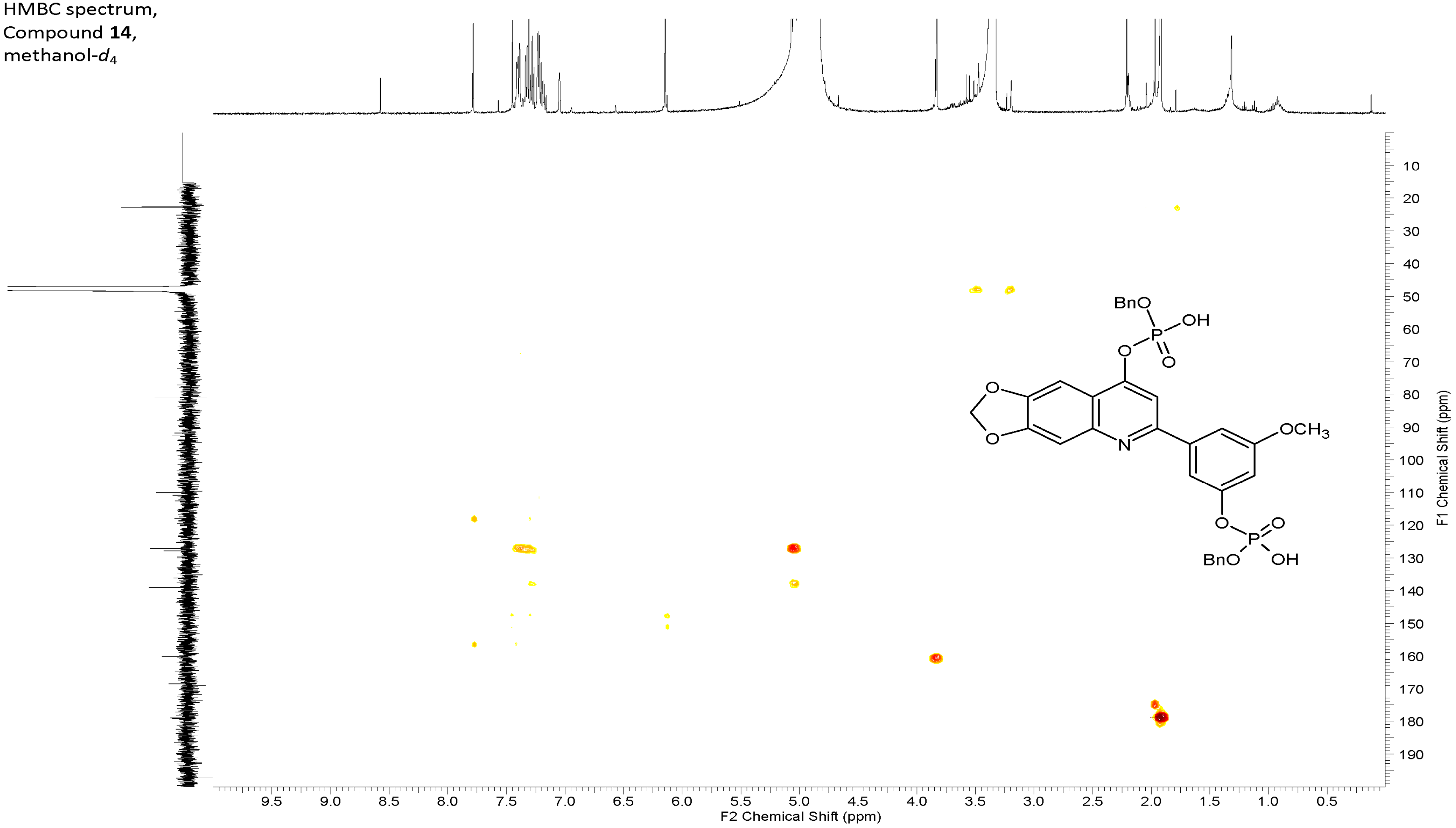

➢ *NMR Spectra of compound **15***

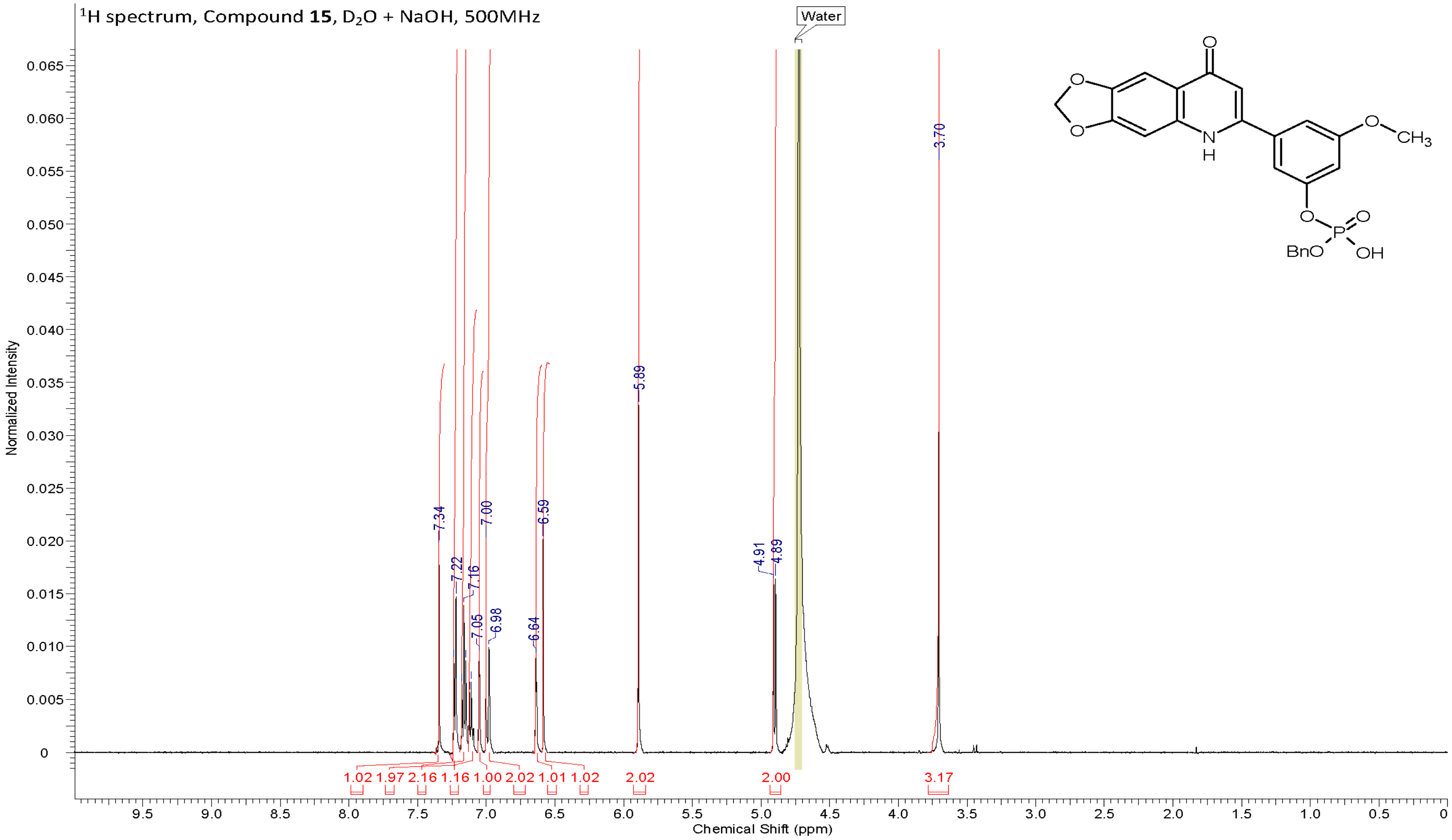

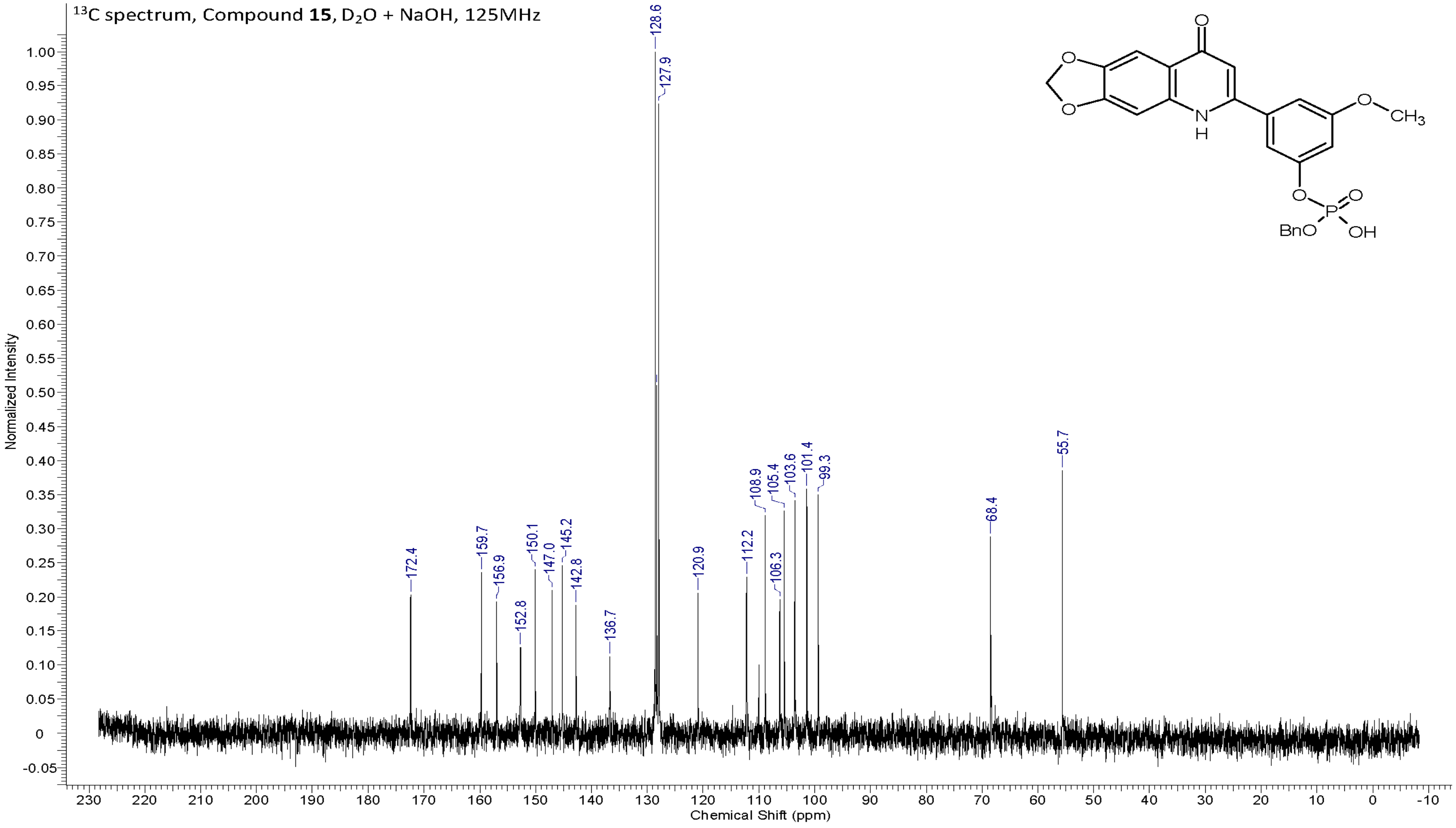

^{13}C spectrum, Compound **15**, D_2O + NaOH, 125MHz
Normalized Intensity
Chemical Shift (ppm)
172.4
159.7
156.9
152.8
150.1
147.0
145.2
142.8
136.7
128.6
127.9
120.9
112.2
108.9
106.3
105.4
103.6
101.4
99.3
68.4
55.7
O
N
H
O
CH3
O
P
O
BnO
OH

HMQC spectrum, Compound **15**, D_2O + NaOH

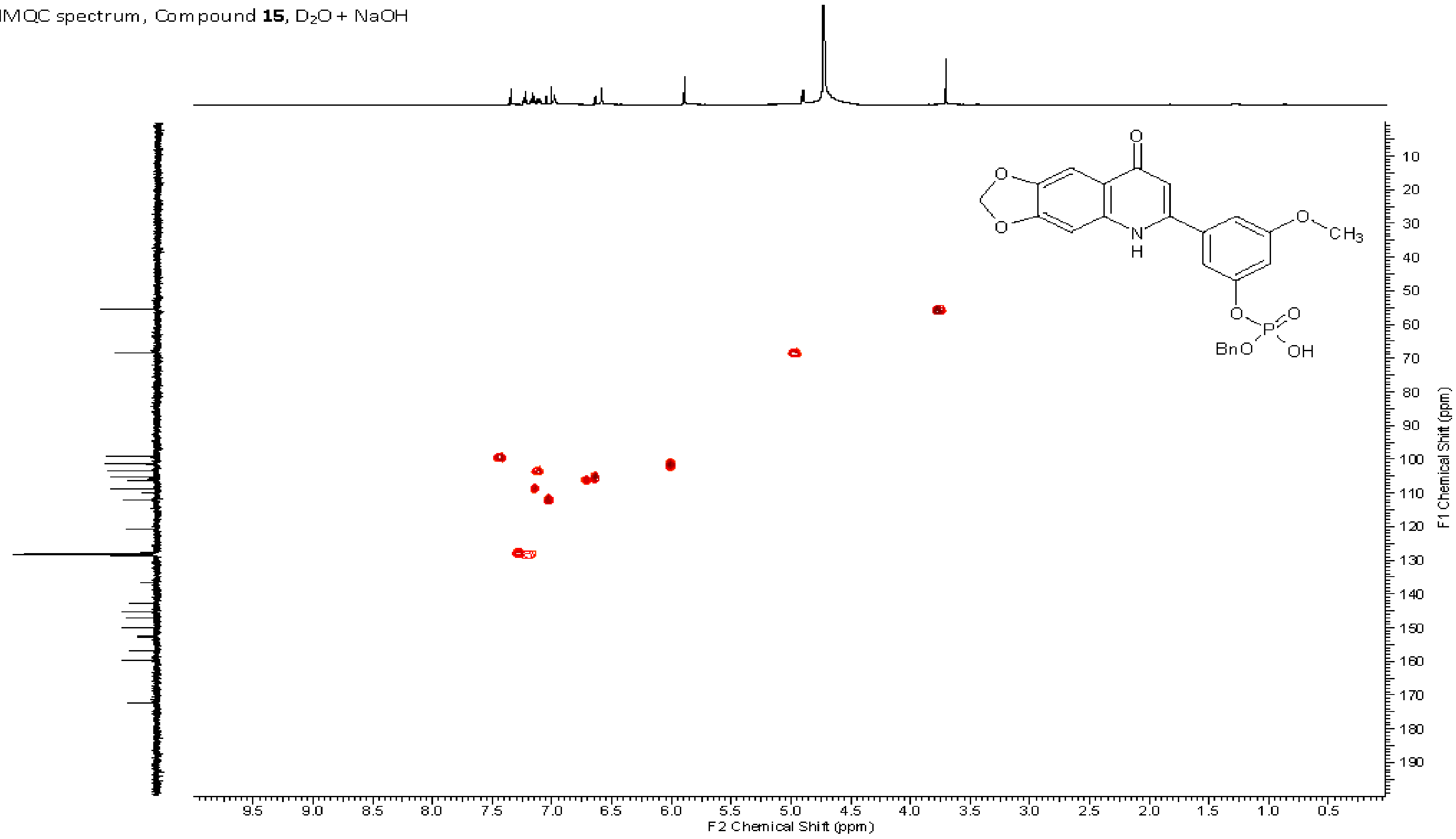

HMBC spectrum, Compound **15**, D_2O + NaOH

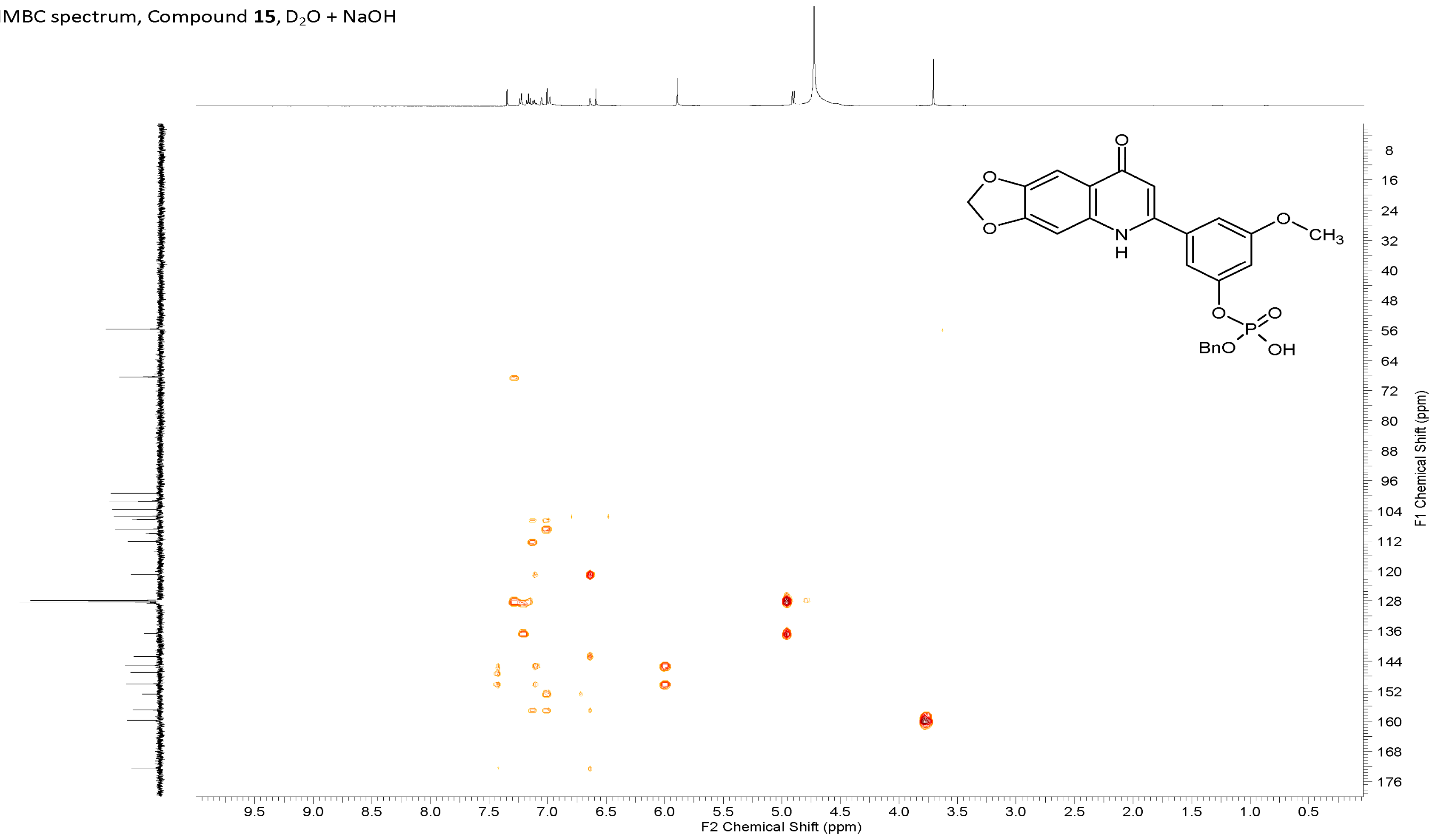

4. The Purity of Compounds 1-4

- ***Compound 1***

Column: Thermo Hypersil ODS column, 5µm, 150 × 4.6 mm, i.d.; Method: Acetonitrile /H_2O (0.02 M $NaHCO_3$), (70/30, v/v); Flow rate: 0.5 mL/min; Detection: 254 nm. The purity analysis was detected by reversed-phase HPLC on Thermo Hypersil ODS column (150 × 4.6 mm i.d) using a acetonitrile/0.02 M $NaHCO_3$ (70/30) mixture of solution as eluent. Flow rate was 0.3 mL/min and UV detector was set at 254 nm. The retention time of **1** was 5.4 min. The purity of **1** was 97.0 %.

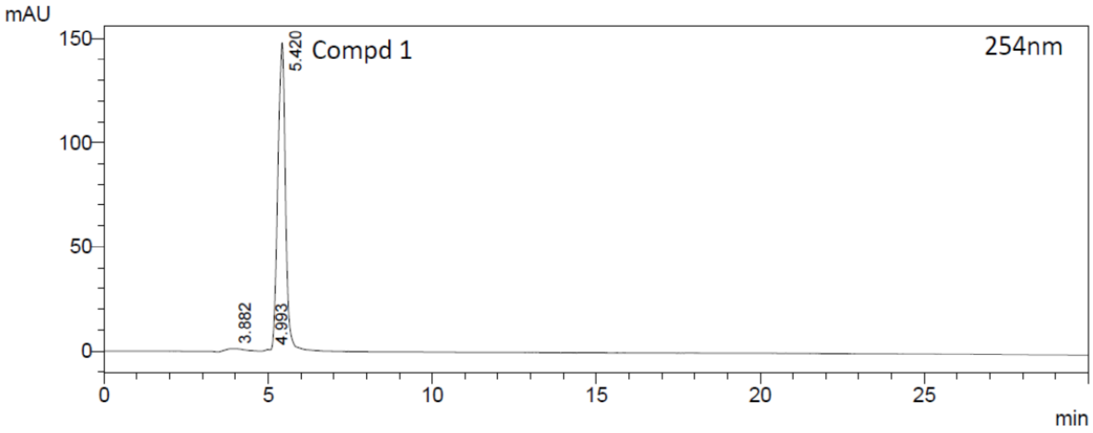

- ***Compound 2***

Column: Nucleodur® C18 HTec, 5µm, 250 × 4.6 mm, i.d.; Method: MeOH/H_2O (0.02 M $NaHCO_3$), (93/7, v/v); Flow rate: 0.5 mL/min; Detection: 254 nm. The purity analysis was detected by reversed-phase HPLC on Nucleodur® C18 HTec (5 µm, 250×4.6 mm i.d) using a MeOH/0.02 M $NaHCO_3$ (93/7) mixture of solution as eluent. The flow rate was 0.5 mL/min and UV detector was set at 254 nm. The retention time of **2** was 11.2 min; the purity of **2** was 99.6 %.

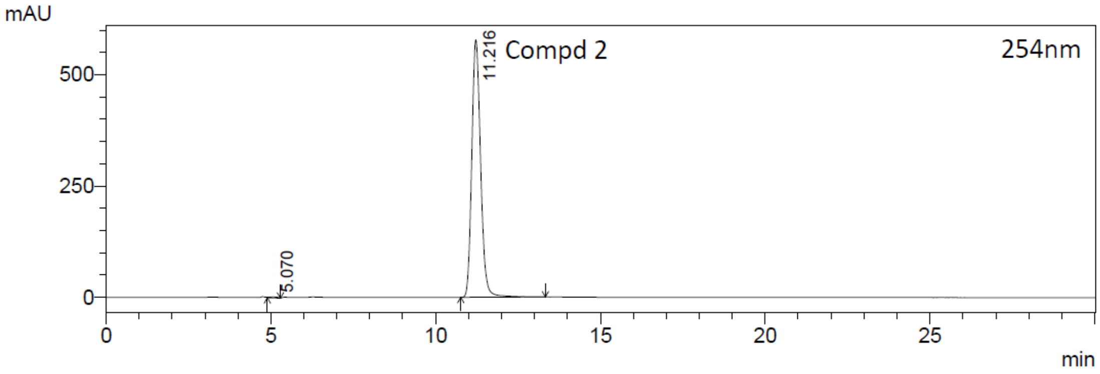

➢ ***Compound 3***

Column: Thermo Hypersil ODS column, 5μm, 150 × 4.6 mm, i.d.; Method: MeOH/H_2O (0.01 M $NaHCO_3$), (95/5, v/v); Flow rate: 0.5 mL/min; Detection: 254 nm. The purity analysis was detected by reversed-phase HPLC on Thermo Hypersil ODS column (150 × 4.6 mm i.d.) using a MeOH/0.01M $NaHCO_3$ (95/5) mixture of solution as eluent. The flow rate was 0.5 mL/min. UV detector was set at 254 nm. The retention time of **3** was 3.9 min; The purity of **3** was 93.1%.

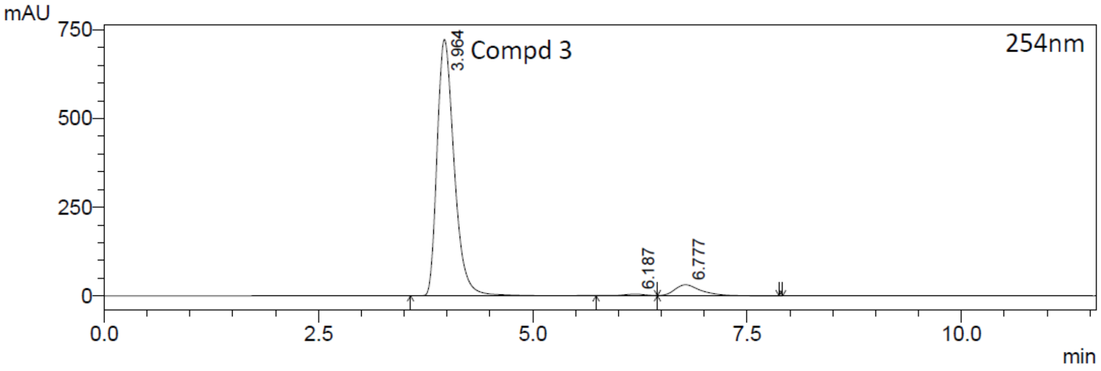

➢ ***Compound 4***

Column: Thermo Hypersil ODS column, 5μm, 150 × 4.6 mm, i.d. Method: MeOH/H_2O (9% $NaHCO_3$), (70/30, v/v); Flow rate: 0.5 mL/min; Detection: 254 nm. The purity analysis was detected by reversed-phase HPLC on Thermo Hypersil ODS column (5 μm, 150 × 4.6 mm i.d) using a MeOH/9% $NaHCO_3$ (70/30) mixture as eluent. The flow rate was 0.5 mL/min and UV detector was set at 254 nm. The retention time of **4** was 2.71 min; the purity of **4** was 99.4%.

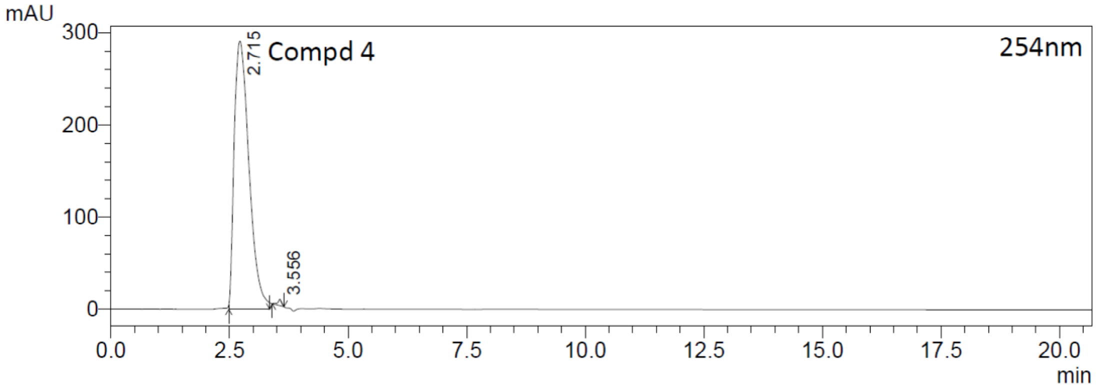

5. Mean Graph Presentation of Differential Data for Compound 1

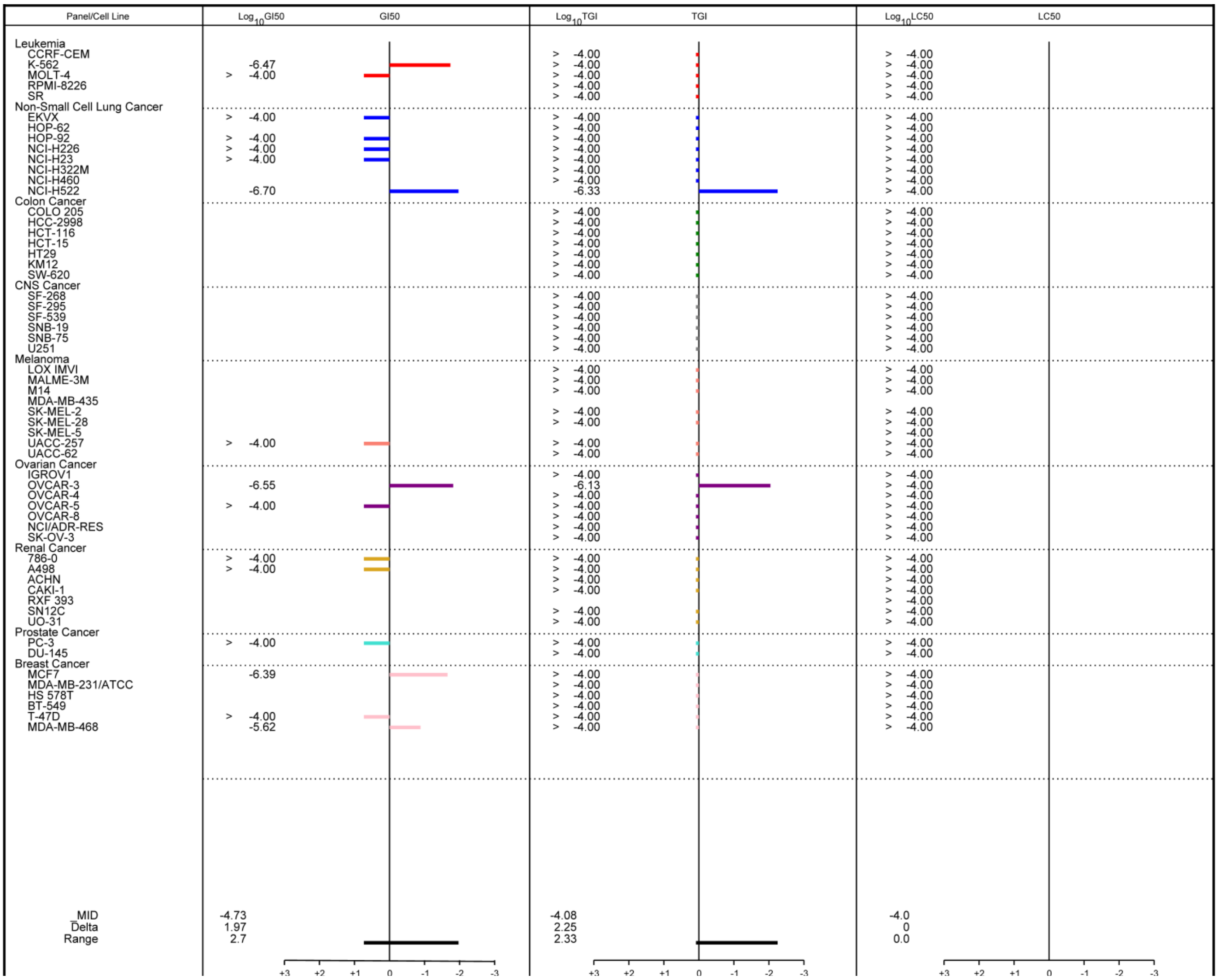

Supplement: Supplementary file 1 [file molecules-18-08028-s001.pdf]
